# Nine new species groups, 15 new species, and one new subspecies of New Guinea diving beetles of the genus *Exocelina* Broun, 1886 (Coleoptera, Dytiscidae, Copelatinae)

**DOI:** 10.3897/zookeys.878.37403

**Published:** 2019-10-07

**Authors:** Helena Shaverdo, Suriani Surbakti, Evie L. Warikar, Katayo Sagata, Michael Balke

**Affiliations:** 1 Naturhistorisches Museum Wien, Burgring 7, 1010 Vienna, Austria; 2 Department of Biology, Universitas Cendrawasih, Jayapura, Papua, Indonesia; 3 University of Papua New Guinea, Port Moresby, Papua New Guinea; 4 SNSB-Zoologische Staatssammlung München, Münchhausenstraße 21, D-81247 Munich, Germany and GeoBioCenter, Ludwig-Maximilians-University, Munich, Germany

**Keywords:** Australasia, distribution, *
Exocelina
*, key, new taxa, species delimitation, systematics

## Abstract

Nine new species groups of *Exocelina* Broun, 1886 from New Guinea are introduced with keys to their representatives. Four groups are monotypic and include three new species: the *E. aipomek* group, the *E. koroba* group: *E. koroba***sp. nov.**, the *E. mekilensis* group: *E. mekilensis***sp. nov.**, and the *E. morobensis* group: *E. morobensis***sp. nov.** The remaining five species groups include 18 species with 12 new species and one new subspecies: the *E. bacchusi* group: *E. akameku***sp. nov.**, *E. oiwa***sp. nov.**, *E. oksibilensis***sp. nov.**, and *E. bacchusi herzogensis***ssp. nov.**; the *E. jaseminae* group: *E. aseki***sp. nov.**, *E. kailaki***sp. nov.**, and *E. pseudojaseminae***sp. nov.**; the *E. larsoni* group: *E. warahulenensis***sp. nov.**; the *E. takime* group: *E. mianminensis***sp. nov.**; and the *E. warasera* group: *E. haia***sp. nov.**, *E. kobau***sp. nov.**, *E. pulchella***sp. nov.**, and *E. warasera***sp. nov.** Diagnoses of five already described species of these groups are provided, as well as comparatives notes on all species. *Exocelina santimontis* (Balke, 1998) **syn. nov.** is a junior synonym of *E. aipomek* (Balke, 1998). Data on the distribution of the species are given, showing that most of the species of these groups occur in the Papua New Guinea.

## Introduction

This paper introduces nine new species groups of *Exocelina* Broun, 1886, completing our assessment of the supraspecific classification of the genus in New Guinea. Four of the species groups here diagnosed are monotypic and include species with distinct morphological characters and which were inferred as separate lineages in our previous molecular phylogenetic analyses (Toussaint et al. 2014, 2015). One of these groups is proposed for the described species, *Exocelina aipomek* (Balke, 1998), and the three remaining for three new species. Five other groups are small and consist of two to five species. Four groups (the *E. bacchusi*, *E. jaseminae*, *E. larsoni* and *E. takime* groups) are proposed for already known species with the addition of eight new species and one new subspecies, and the fourth group, the *E. warasera* group, includes only four new species. We provide a diagnosis for the complex of groups treated herein and notes on their phylogeny, as well as morphological diagnoses for each group separately. All species of the groups are treated, including comparative notes and detailed descriptions for the new species. Identification keys are presented for the groups with more than one species. Including the results of this work, 140 species of *Exocelina* are now described from New Guinea and 195 species worldwide. As in most of our previous papers on the genus (Shaverdo et al. 2012, 2013, 2014, 2016a, b, c, 2017, 2018), all species data will be presented on the species-id.net portal automatically created by ZooKeys with the publication of this paper.

## Materials and methods

The present work is based on material from the following collections:

**BMNH** The Natural History Museum, London, UK

**CGW** Collection of Günther Wewalka, Vienna, Austria

**KSP** Koleksi Serangga Papua, at the Biology Department of Universitas Cenderawasih (UNCEN), Waena, Papua, Indonesia

**MZB** Museum Zoologicum Bogoriense, Cibinong, Indonesia

**NHMW** Naturhistorisches Museum Wien, Vienna, Austria

**ZSM** Zoologische Staatsammlung München, Munich, Germany

Our methods follow those described in detail in our previous articles (Shaverdo et al. 2012, 2014; Shaverdo and Balke 2014). The terminology to denote the orientation of the genitalia (ventral for median lobe and dorsal and external for paramere) follows Miller and Nilsson (2003). All specimen data are quoted as they appear on the labels attached to the specimens. Label text is cited using quotation marks. Comments in square brackets are ours. The following abbreviations were used: TL (total body length), TL-H (total body length without head), MW (maximum body width), and hw (handwritten).

The keys are based mostly on the male characters. In many cases, females cannot be assigned to species due to similarity of their external and internal structures (for female genitalia see Shaverdo et al. 2005: fig. 17a, b). Some species are rather similar in point of external morphology, therefore, in most cases the male genitalia need to be studied for reliable species identification.

## Checklist and distribution of the species

Abbreviations: IN – Indonesia; PNG – Papua New Guinea.

**Table:** 

***Exocelina aipomek* group**		
1.	*Exocelina aipomek* (Balke, 1998)	IN: Papua: Pegunungan Bintang; PNG: Sandaun
***Exocelina koroba* group**		
2.	*Exocelina koroba* sp. nov.	PNG: Hela
***Exocelina mekilensis* group**		
3.	*Exocelina mekilensis* sp. nov.	PNG: Sandaun
***Exocelina morobensis* group**		
4.	*Exocelina morobensis* sp. nov.	PNG: Morobe
***Exocelina bacchusi* group**		
5.	*Exocelina akameku* sp. nov.	PNG: Madang
6.	*Exocelina bacchusi* (Balke, 1998)	PNG: Madang, Simbu, Eastern Highlands, Morobe, Gulf
6a.	*Exocelina bacchusi herzogensis* ssp. nov.	PNG: Morobe, Central
7.	*Exocelina erteldi* (Balke, 1998)	IN: Papua: Pegunungan Bintang
8.	*Exocelina oiwa* sp. nov.	PNG: Morobe
9.	*Exocelina oksibilensis* sp. nov.	IN: Papua: Pegunungan Bintang
***Exocelina jaseminae* group**		
10.	*Exocelina aseki* sp. nov.	PNG: Morobe
11.	*Exocelina jaseminae* (Balke, 1998)	PNG: Morobe, Eastern Highlands
12.	*Exocelina kailaki* sp. nov.	PNG: Central
13.	*Exocelina pseudojaseminae* sp. nov.	PNG: Central
***Exocelina larsoni* group**		
14.	*Exocelina larsoni* (Balke, 1998)	PNG: Madang, Eastern Highlands
15.	*Exocelina nomax* (J. Balfour-Browne, 1939)	PNG: Central, National Capital District
16.	*Exocelina warahulenensis* sp. nov.	PNG: Simbu, Eastern Highlands
***Exocelina takime* group**		
17.	*Exocelina mianminensis* sp. nov.	PNG: Sandaun
18.	*Exocelina takime* (Balke, 1998)	IN: Papua: Pegunungan Bintang
***Exocelina warasera* group**		
19.	*Exocelina haia* sp. nov.	PNG: Simbu
20.	*Exocelina kobau* sp. nov.	PNG: Morobe
21.	*Exocelina pulchella* sp. nov.	PNG: Central
22.	*Exocelina warasera* sp. nov.	PNG: Simbu, Eastern Highlands

## General diagnostic characters of the treated groups and notes on their phylogeny

Here, we provide general diagnostic characters for all representatives of the groups, which can be used to separate them from some of the previously studied groups. To complete diagnoses, special diagnostic characters for each group, mainly based on shape of the median lobe and shape and setation of the parameres, are provided below, before the species treatments.

– beetles small or medium-sized (TL-H 2.85–4.5 mm);

– habitus elongate to oval, in most species oblong-oval; with rounded pronotal and elytral sides, body outline continuous;

– pronotum short, trapezoidal, with posterior angles not drawn backwards;

– pronotum and elytra without striae or strioles;

– antennomeres not modified, simple;

– male protarsomeres 1–3 not expanded laterally;

– male protarsomere 4 cylindrical, narrow, with a large, hook-like to thin, long, slightly curved anterolateral seta;

– male protarsomere 5 not modified, long and narrow, sometimes slightly concave ventrally;

– median lobe of aedeagus with continuous outline in ventral and lateral view;

– ventral sclerite of median lobe more or less deeply divided apically.

All treated species groups (except for the monotypic *E. koroba* and *E. mekilensis* groups) are separate lineages within a monophyletic complex, including the *E. danae* and *E. monae* groups (Toussaint et al. 2014, 2015). Although altogether they do not form a monophyletic complex, all of them (except one species *Exocelina warahulenensis* sp. nov.) have a character that distinguishes them from the representatives of the *E. danae* and *E. monae* groups – absence of the setation on the median lobe of the aedeagus. *Exocelina koroba* sp. nov. and *E. mekilensis* sp. nov. have a very distinct morphology, especially of the male genitalia, and belong to a completely different clade of New Guinea *Exocelina*. They form separate lineages within a monophyletic complex, which also includes some species of the *E. casuarina* group (Toussaint et al. 2014, 2015; Shaverdo et al. 2018).

## Diagnostic characters of the species groups, species descriptions and comparative notes

### Monotypic groups

#### *Exocelina aipomek* group

This group is characterised by extremely fine, inconspicuous dorsal punctation, pronotum with distinct lateral bead; median lobe of aedeagus without setation, simple, with rounded apex in ventral view; apexes of ventral sclerites of median lobe almost equal; paramere with distinct dorsal notch and large, long subdistal part with numerous strong setae, proximal setae more or distinct.

##### 1. Exocelina aipomek

Taxon classificationAnimaliaColeopteraDytiscidae

(Balke, 1998)

E298CAFD-44CB-561F-BDB4-CFB02D697F45

Figs 1 , 5 , 6–7

Copelatus (Papuadytes) aipomek Balke, 1998: 322; Nilsson 2001: 76 (catalogue).
Papuadytes aipomek (Balke, 1998): Nilsson and Fery 2006: 56 (comb. nov.).
Exocelina aipomek (Balke, 1998): Nilsson 2007: 33 (comb. nov.).
Exocelina aipomek MB3726: Toussaint et al. 2014: supplementary figs 1–4, tab. 2; Toussaint et al. 2015: supplementary figs S1, S2, tab. S3, and information S5, S6.
Copelatus (Papuadytes) santimontis Balke, 1998: 335; Nilsson 2001: 77 (catalogue) syn. nov.
Papuadytes santimontis (Balke, 1998): Nilsson and Fery 2006: 56 (comb. nov.).
Exocelina santimontis (Balke, 1998): Nilsson 2007: 34 (comb. nov.).

###### Type locality.

Indonesia: Papua Province: Pegunungan Bintang Regency, Aipomek, 04°27'S, 140°01'E, 1800 m a.s.l.

###### Type material studied.

***Exocelina aipomek***: *Holotype*: male “IRIAN JAYA Aipomek Area 140°01'E 04°27'S”, “Aipomek, 1800m 30./31.8.1992 leg.Balke (30)”, “HOLOTYPUS” [red], “Copelatusaipomek Balke des. 1997” [red] (NHMW). *Paratypes*: 3 males, 1 female with the same label as the holotype and additionally with red labels “Paratypus Copelatusaipomek Balke des. 1997”, one of the males with two additional labels “M.Balke 3272” [green] and “M.Balke 6403 DNA” [green text] (NHMW). ***Exocelina santimontis***: *Holotype*: male “IRIAN JAYA: 1.10.1993 Eme Gebiet Okloma, 1500m”, “ca. 139°55'E 04°14'S leg. M. Balke (28)”, “HOLOTYPUS” [red], “Copelatussantimontis Balke des. 1997” [red] (NHMW). *Paratypes*: 8 males with the same label as the holotype and additionally with red labels “Paratypus Copelatussantimontis Balke des. 1997”, one of the males with two additional labels “M.Balke 3289” [green] and “M.Balke 6412 DNA” [green text], another male with an additional green label “M.Balke 3288” (NHMW). 1 male “IRIAN JAVA: Borme Tarmlu 1500m 6.9.1993”, “ca. 140°25'E 04°24'S leg. M. Balke (4–6)”, “Paratypus Copelatussantimontis Balke des. 1997” (NHMW). 1 male “IRIAN JAYA: 22.9.1993 Bime – Calab Gebiet, Bime, 1400m”, “ca. 140°12'E 04°20'S, leg. M. Balke (16)”, “Paratypus Copelatussantimontis Balke des. 1997” [red] (NHMW)

**Figures 1–4.**
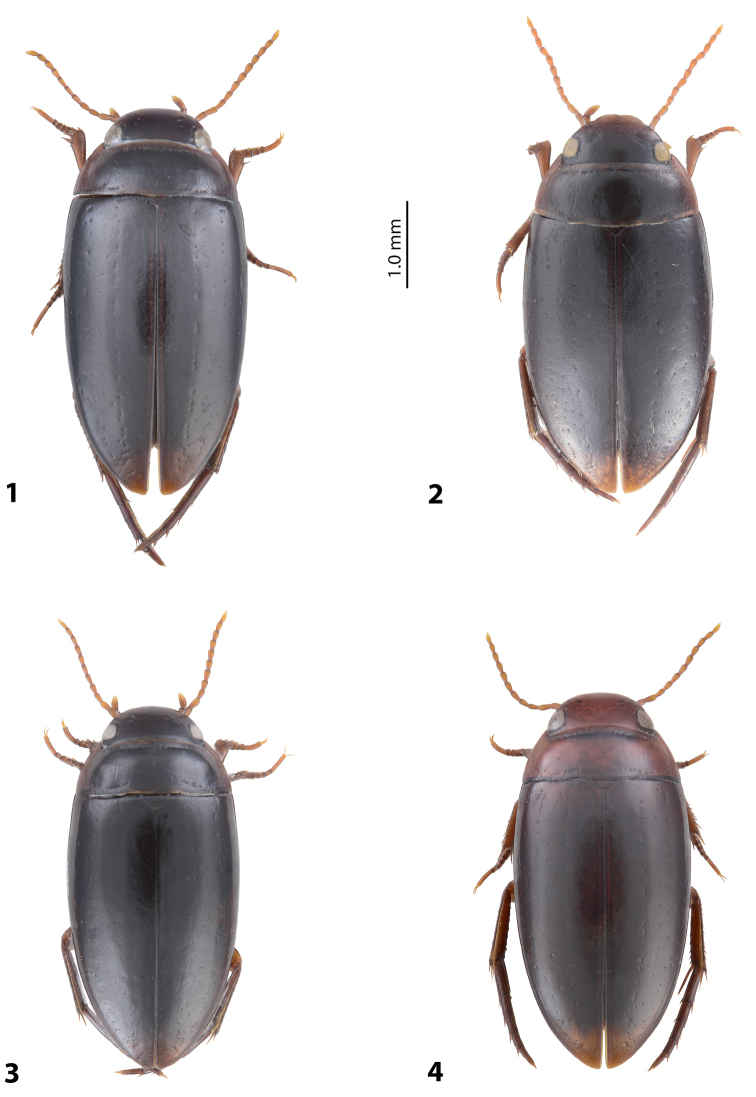
Habitus and colouration **1***Exocelina aipomek* (Balke, 1998) **2***E. mekilensis* sp. nov. **3***E. koroba* sp. nov. **4***E. morobensis* sp. nov.

**Figure 5.**
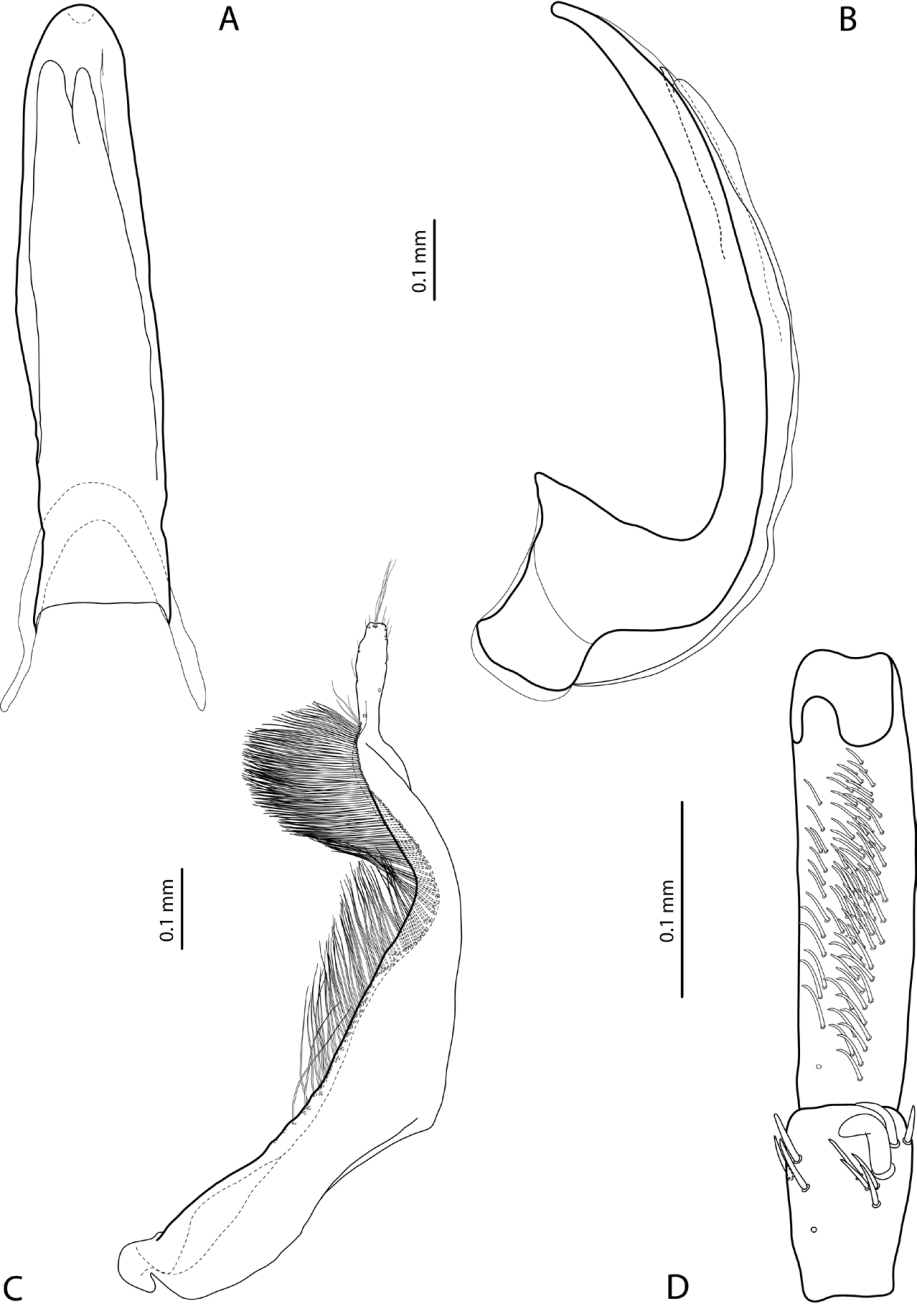
*Exocelina aipomek* (Balke, 1998), paratype **A** median lobe in ventral view **B** median lobe in lateral view **C** paramere in external view **D** male protarsomeres 4–5 in ventral view.

###### Additional material.

**PNG: Sandaun**: 2 males “Papua New Guinea: Sandaun, Ofektaman, 820m, 17.x.2008, 5.04.113S 141.35.841E, Ibalim (PNG 190)”, one with an additional green label “M.Balke 3727” (ZSM).

###### Females of doubtful identity.

**IN: Papua: Pegunungan Bintang**: 20 females “IRIAN JAYA: 22.9.1993 Bime – Calab Gebiet, Bime, 1400m”, “ca. 140°12'E 04°20'S, leg. M. Balke (16)”, “Paratypus Copelatus rivulus sp.n. Balke des. 1997” [red] (NHMW); these females are a mixture of two species: *E. damantiensis* (Balke, 1998) and *E. aipomek*. 13 females “IRIAN JAYA: 22.9.1993 Bime – Calab Gebiet, Bime, 1400m”, “ca. 140°12'E 04°20'S, leg. M. Balke (16)” (NHMW); these females are a mixture of two species: *E. damantiensis* and *E. aipomek*. 1 female “IRIAN JAVA: Borme Tarmlu 1500m 6.9.1993”, “ca. 140°25'E 04°24'S leg. M. Balke (4–6)” (NHMW). 3 females “IRIAN JAVA: Borme Tarmlu 1500m 6.9.1993”, “ca. 140°25'E 04°24'S leg. M. Balke (4)” (NHMW). 2 females “IRIAN JAVA: Borme Tarmlu 1500m 6.9.1993”, “ca. 140°25'E 04°24'S leg. M. Balke (6)” (NHMW). These females are a mixture of four species: *E. damantiensis*, *E. ketembang* (Balke, 1998), *E. aipomek*, and *E. danae* (Balke, 1998). 1 male (no genitals), 27 females “IRIAN JAYA: 1.10.1993 Eme Gebiet Okloma, 1500m”, “ca. 139°55'E 04°14'S, leg. M. Balke (28)” (NHMW); these specimens are a mixture of three species: *E. damantiensis*, *E. ketembang*, and *E. aipomek*. 13 females “IRIAN JAYA: 22.9.1993 Bime – Calab Gebiet, Bime, 1400m”, “ca. 140°12'E 04°20'S, leg. M. Balke (16)” (NHMW). 2 females “IRIAN JAYA, 24.–26.9.1993 Eipomek [sic!] Gebiet Eipomek [sic!] - Diruemna”, “ca. 140°01'E 04°27'S 1800–2600m, leg. M. Balke (21–22)” (NHMW). These females are a mixture of two species: *E. damantiensis* and *E. aipomek*. **PNG: Sandaun**: 7 females “Papua New Guinea: Sandaun, Ofektaman, 820m, 17.x.2008, 5.04.113S 141.35.841E, Ibalim (PNG 190)” (ZSM); these females might belong to three species: *E. sandaunensis* Shaverdo & Balke, 2014, *E. aipomek*, and *E. ketembang* (Balke, 1998).

###### Diagnosis.

For complete description, see Balke (1998: 322). Beetle medium-sized (TL-H 4.0–4.35 mm), oblong-oval; piceous, sometimes with paler pronotal sides; dorsally shiny, with extremely fine, inconspicuous punctation and weakly impressed microreticulation; pronotum with distinct lateral bead (Fig. 1); male protarsomere 4 with large, thick, strongly curved anterolateral hook-like seta; male protarsomere 5 ventrally with anterior band of more than 60 and posterior row of 14 relatively long setae (Fig. 5D); median lobe simple, in lateral view, evenly tapering to broadly pointed, somehow elongate and gently curved downwards apex, in ventral view, apex more or less rounded; paramere with distinct dorsal notch and large, long subdistal part, dorsal setae numerous and strong, subdistal slightly denser and longer than proximal ones, the latter more or less distinct (Fig. 5A–C).

###### Variability.

**Figures 6, 7.**
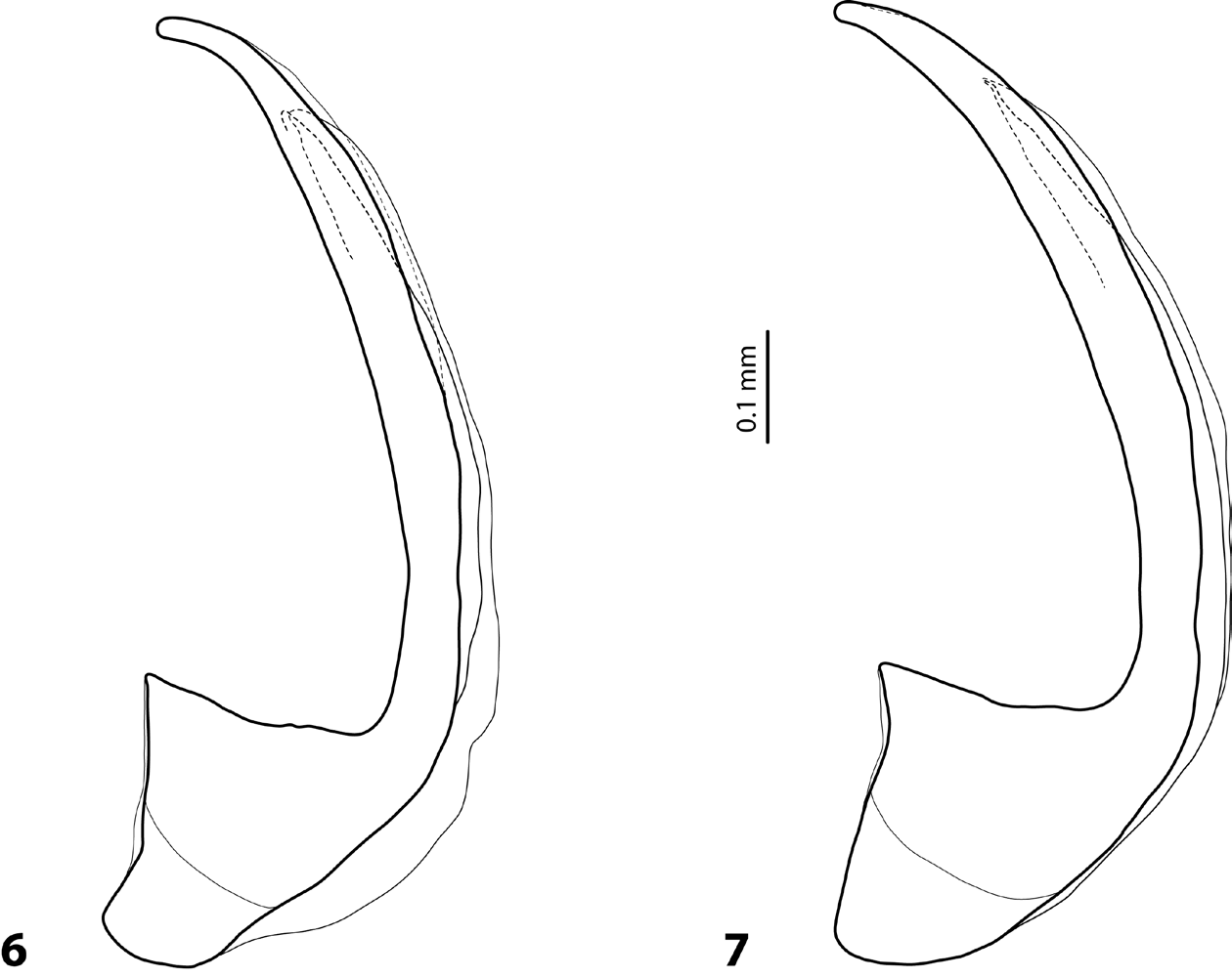
*Exocelina aipomek* (Balke, 1998), median lobe in lateral view **6** specimen from Sandaun, Ofektaman **7** paratype of *E. sanctimontis* (Balke, 1998).

The species shows variability within and between populations in shape of the apex of the median lobe, which can be shorter or more elongate (Figs 6, 7).

###### Affinities.

In the area of its distribution, *E. aipomek* co-occurs with numerous species: *E. ascendens* (Balke, 1998), *E. fume* (Balke, 1998), *E. takime*, species of the *E. bacchusi*, *E. ekari*, *E. danae*, *E. broschii*, *E. okbapensis*, and *E. aipo* groups. The species can be distinguished from them by its body size, form and colouration, inconspicuous dorsal punctation, and weakly impressed microreticulation, presence of the pronotal bead, shape and setation of its median lobe, paramere, and male protarsomere 4.

###### Distribution.

Indonesia: Papua Province: Pegunungan Bintang Regency and Papua New Guinea: Sandaun Province (Fig. 11).

#### *Exocelina koroba* group

This group is characterised by relatively dense and coarse dorsal punctation; pronotum with distinct lateral bead; median lobe of aedeagus without setation, with apex thick, short, pointed and strongly curved downwards in lateral view; apexes of ventral sclerites of median lobe almost equal; paramere with distinct notch on dorsal side, subdistal part relatively large, rounded, with dense and strong setae, proximal setae inconspicuous.

##### 2. Exocelina koroba

Taxon classificationAnimaliaColeopteraDytiscidae

Shaverdo & Balke sp. nov.

341A511B-7DCC-5FFE-9F17-3732B4E29950

http://zoobank.org/B6A930A9-0B56-48D9-8C04-4BD72F56A750

Figs 2 , 8

Exocelina undescribed sp. MB1292: Toussaint et al. 2014: supplementary figs 1–4, tab. 2; Toussaint et al. 2015: supplementary figs S1, S2, tab. S3, and information S5, S6.

###### Type locality.

Papua New Guinea: Hela Province, Hedamali, ca. 05°41.85'S, 142°43.84'E, 1700–1900 m a.s.l.

###### Type material.

*Holotype*: male “PAPUA N.G.: 6.–9.5.1998 Southern Highl. Prov. Tari-Koroba, Hedemari [Hedamali] 1700–1900 m, leg. Riedel” (NHMW). *Paratypes*: 1 male “Papua New Guinea: Southern Highlands, Koroba, 1600 m, 15.v.1994, 05.41.854S 142.43.836E, Balke (PNG 66)”, “DNA M Balke 1292” (ZSM).

###### Description.

*Body size and form*: Beetle medium-sized: TL-H 3.95–4.4 mm, TL 4.4–4.55 mm, MW 2.2–2.35 mm (holotype: TL-H 3.95 mm, TL 4.4 mm, MW 2.2 mm), with oblong-oval habitus.

*Colouration*: Piceous, with paler sides of pronotum. Head piceous, paler anteriorly; pronotum piceous, with brown sides; elytra piceous, with reddish sutural lines; head appendages and legs proximally reddish, legs distally darker, reddish brown (Fig. 2). Teneral specimen paler, brown.

*Surface sculpture*: Submatt dorsally, with relatively dense and coarse punctation and evident microreticulation. Head with relatively dense and coarse punctation (spaces between punctures 1–2 times size of punctures); diameter of punctures almost equal to diameter of cells of microreticulation. Pronotum with finer, sparser punctation, and more evenly distributed punctation than on head. Elytra with coarser punctation than on pronotum. Pronotum and elytra with distinct microreticulation. Head with microreticulation slightly stronger. Metaventrite, metacoxae, and abdominal ventrites distinctly microreticulate. Metacoxal plates with longitudinal strioles and weak transverse wrinkles; abdominal ventrites with strioles. Venter with inconspicuous punctation, more evident on metacoxal plates and two last abdominal ventrites.

*Structures*: Pronotum with distinct lateral bead. Base of prosternum and neck of prosternal process with distinct ridge, slightly rounded and with few transverse strioles anteriorly, without anterolateral extensions. Blade of prosternal process lanceolate, relatively broad, convex, with distinct bead and few setae laterally. Abdominal ventrite 6 slightly truncate.

**Figure 8.**
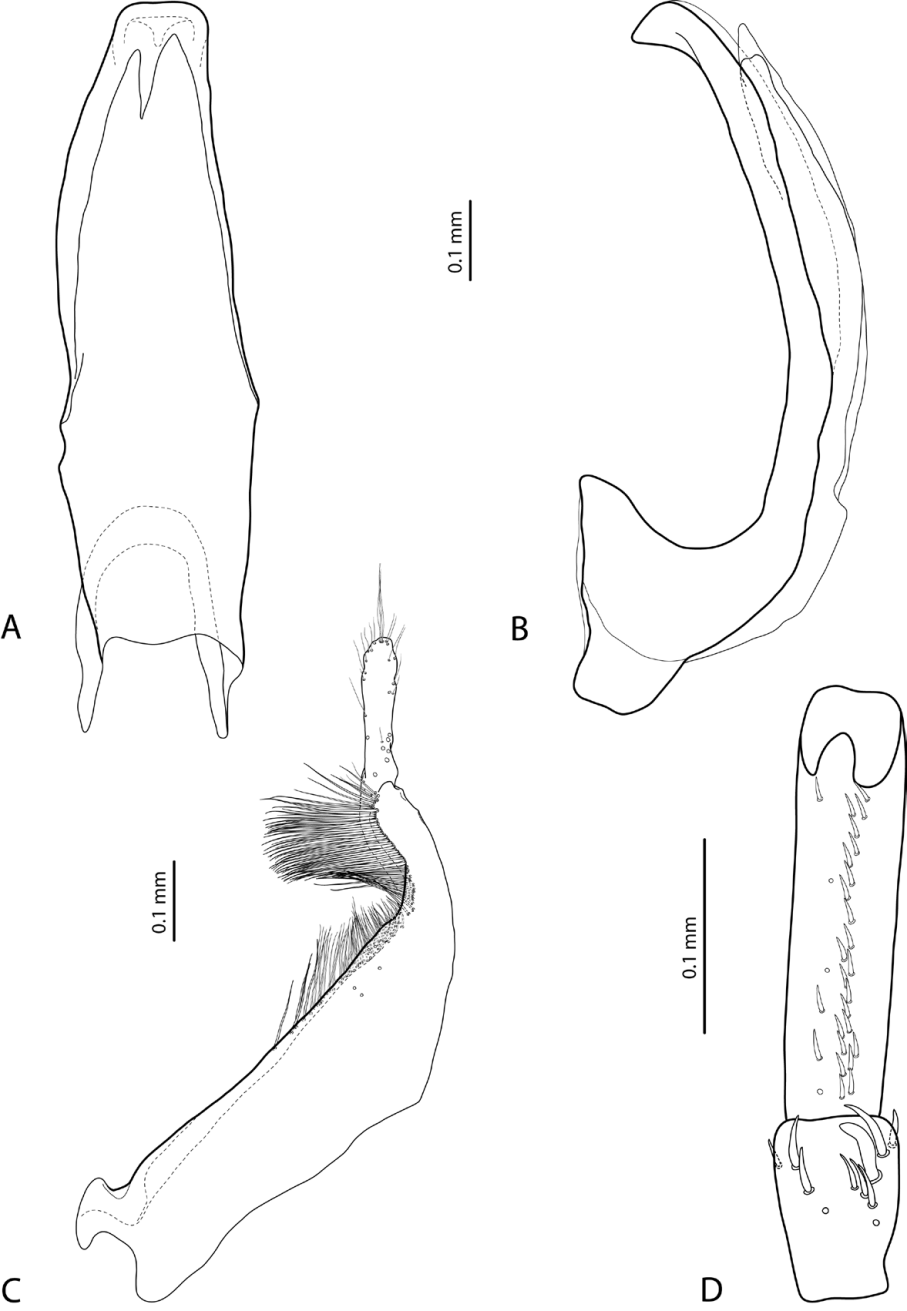
*Exocelina koroba* sp. nov. **A** median lobe in ventral view **B** median lobe in lateral view **C** paramere in external view **D** male protarsomeres 4–5 in ventral view.

*Male*: Protarsomere 4 with rather small, slightly curved anterolateral hook-like seta. Protarsomere 5 ventrally with anterior row of 23 short setae and posterior row of 6 setae (Fig. 8D). Abdominal ventrite 6 with 12–15 lateral striae on each side. Median lobe slightly curved, with apex thick, short, pointed and strongly curved downwards in lateral view (Fig. 8A–B). Paramere with distinct notch on dorsal side, subdistal part relatively large, rounded, with dense and strong setae, proximal setae thin and sparse, inconspicuous. (Fig. 8C).

*Female*: Unknown.

###### Affinities.

The species can be distinguished from the species co-occurring in the same area (*E. pseudoedeltraudae* Shaverdo & Balke, 2014, *E. tariensis* Shaverdo & Balke, 2014, *E. marinae* (Shaverdo, Sagata & Balke, 2005), and *E. pseudomarinae* Shaverdo, Sagata & Balke, 2016) by size, relatively dense and coarse dorsal punctation, not modified male antennae, and the shape and setation of its median lobe and paramere.

###### Distribution.

Papua New Guinea: Hela Province, Koroba area (Fig. 11).

###### Etymology.

The species is named after Koroba Village. The name is a noun in the nominative singular standing in apposition.

#### *Exocelina mekilensis* group

This group is characterised by fine and sparse dorsal punctation; pronotum without lateral bead; median lobe of aedeagus without setation, simple; in lateral view, apex thick, short and slightly curved downwards, its minuscule tip curved upwards; apexes of ventral sclerites of median lobe slightly unequal: left one slightly longer that right one; paramere without dorsal notch, evenly tapering to distal part, with numerous small spines and without long setae.

##### 3. Exocelina mekilensis

Taxon classificationAnimaliaColeopteraDytiscidae

Shaverdo & Balke sp. nov.

A9608F8E-FD8A-5A1D-8717-FBE6E87EDF24

http://zoobank.org/4833A1FB-F7A6-4ED8-ACA2-442719E2A641

Figs 3 , 9

Exocelina undescribed sp. MB0686: Toussaint et al. 2014: supplementary figs 1–4, tab. 2; Toussaint et al. 2015: supplementary figs S1, S2, tab. S3, and information S5, S6.

###### Type locality.

Papua New Guinea: Sandaun Province, Ofektaman, 05°04.11'S, 141°35.84'E, 820 m a.s.l.

###### Type material.

*Holotype*: male “Papua New Guinea: Sandaun, Ofektaman, 820m, 17.x.2008, 5.04.113S 141.35.841E, Ibalim (PNG 190)”, “DNA M.Balke 3723” (ZSM). *Paratypes*: 1 male with the same label as the holotype (NHMW). 1 male “Papua New Guinea: Sandaun, Sokamin4, 1200m, 19.x.2003, 4 50.845S 141 37.865E, K. Sagata (WB102)”, “DNA M. Balke 665” [green text] (ZSM). 1 male, 4 females “Papua New Guinea: Sandaun, MekilW100, 1718m, 14.x.2003, 4 48.637S 141 38.994E, K. Sagata (WB19)” (NHMW, ZSM). 1 male “DNA M. Balke 686” [green text], “Papua New Guinea: Sandaun, Mekil (WB19), 13.x.2003, K. Sagata, DNA M Balke: MB 686” (ZSM).

###### Description.

*Body size and form*: Beetle medium-sized: TL-H 3.85–4.4 mm, TL 3.45–3.95 mm, MW 1.8–2.05 mm (holotype: TL-H 3.8 mm, TL 4.2 mm, MW 2.0 mm), with oblong-oval habitus.

*Colouration*: Dark brown, with paler sides of pronotum and head anteriorly. Head dark brown, piceous posteriorly; pronotum dark brown, with brown sides; elytra uniformly dark brown; head appendages and legs proximally reddish, legs distally darker, reddish brown (Fig. 3). Teneral specimen paler, brown to reddish brown with yellowish pronotal sides.

*Surface sculpture*: Shiny dorsally, with fine, sparse punctation and weakly impressed microreticulation. Head with relatively fine and sparse punctation (spaces between punctures 2–3 times size of punctures); diameter of punctures almost equal to or smaller than diameter of cells of microreticulation. Pronotum and elytra with much finer and sparser punctation than on head, inconspicuous. Pronotum and elytra with weakly impressed microreticulation. Head with microreticulation slightly stronger. Metaventrite, metacoxae, and abdominal ventrites distinctly microreticulate. Metacoxal plates with longitudinal strioles and weak transverse wrinkles; abdominal ventrites with strioles. Venter with extremely inconspicuous punctation, more evident on metacoxal plates and two last abdominal ventrites.

*Structures*: Pronotum without lateral bead. Base of prosternum and neck of prosternal process with distinct ridge, rounded anteriorly. Blade of prosternal process lanceolate, relatively narrow, slightly convex, with distinct bead and few setae laterally. Abdominal ventrite 6 slightly truncate.

**Figure 9.**
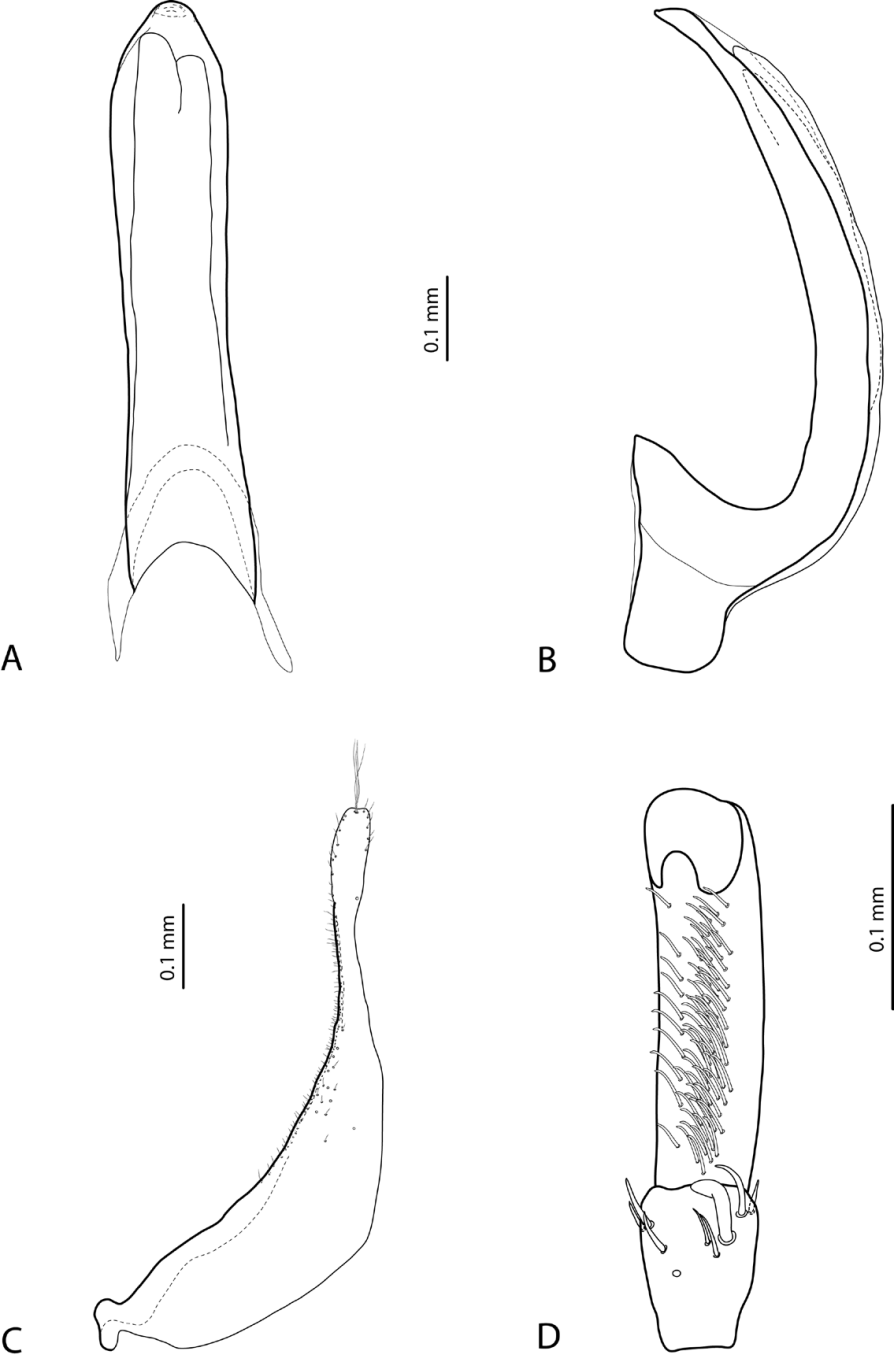
*Exocelina mekilensis* sp. nov. **A** median lobe in ventral view **B** median lobe in lateral view **C** paramere in external view **D** male protarsomeres 4–5 in ventral view.

*Male*: Protarsomere 4 with large, thick, strongly curved anterolateral hook-like seta. Protarsomere 5 ventrally with anterior band of ca. 60 and posterior row of ten relatively long setae (Fig. 9D). Abdominal ventrite 6 with 6–10 lateral striae on each side. Median lobe simple, slightly curved, in lateral view, apex thick, short and slightly curved downwards, its minuscule tip curved upwards (Fig. 9A, B). Paramere without notch on dorsal side, evenly tapering to distal part, with numerous small spines and without long setae (Fig. 9C).

*Female*: Without evident differences in external morphology from males, except for not modified protarsi and abdominal ventrite 6 without striae.

###### Affinities.

From most species co-occurring in the same area (*E. sandaunensis*, *E. tabubilensis* Shaverdo & Balke, 2014, *E. damantiensis*, *E. okbapensis* Shaverdo & Balke, 2017, and *E. may* Shaverdo & Balke, 2017), *E. mekilensis* sp. nov. can be distinguished by its smaller size and absence of the pronotal bead, and simple male antennae. From the species without pronotal bead (*E. pseudobifidae* Shaverdo & Balke, 2014, *E. pseudoeme* Shaverdo & Balke, 2014, and *E. ibalimi* Shaverdo & Balke, 2018), it can be differentiated by the shape and setation of its median lobe and paramere, which are very characteristic and resemble those of the *E. ullrichi* group (Shaverdo and Balke 2014).

###### Distribution.

Papua New Guinea: Sandaun Province (Fig. 11).

###### Etymology.

The species is named after Mekil Village where most specimens of the species were found. The name is an adjective in the nominative singular.

#### *Exocelina morobensis* group

This group is characterised by fine and sparse dorsal punctation; pronotum with narrow lateral bead; median lobe of aedeagus without setation, evenly curved, rather thin, lateral margins thickened proximally; in lateral view, its apex elongate, slightly thickened and rounded, in ventral view, median lobe broad proximally and distinctly narrowed in distal half, its apex bluntly pointed; apexes of ventral sclerites of median lobe almost equal; paramere slightly concave on dorsal side, its subdistal part with dense, strong setae, proximal setae weaker, less distinct.

##### 4. Exocelina morobensis

Taxon classificationAnimaliaColeopteraDytiscidae

Shaverdo & Balke sp. nov.

B4CF6554-1BFD-5C3A-9470-C9317EA6D974

http://zoobank.org/3DA0BA42-2D2B-4B41-A31C-92C74250DDD9

Figs 4 , 10

Exocelina undescribed sp. MB1313: Toussaint et al. 2014: supplementary figs 1–4, tab. 2; Toussaint et al. 2015: supplementary figs S1, S2, tab. S3, and information S5, S6.
Exocelina undescribed sp. MB3840: Toussaint et al. 2014: supplementary figs 1–4, tab. 2; Toussaint et al. 2015: supplementary figs S1, S2, tab. S3, and information S5, S6.

###### Type locality.

Papua New Guinea: Morobe Province, Garaina, 07°51'03"S, 147°07'01"E, 720 m a.s.l.

###### Type material.

*Holotype*: male “Papua New Guinea Garaina, 720m, vi.2008, 07.51.032S 147.07.007E Ibalim & Sosanika PNG216” (ZSM). *Paratypes*: **Morobe**: 72 males, 83 females with the same label as the holotype (NHMW, ZSM). 64 males, 69 females “Papua New Guinea: Morobe, Garaina, 800m, vi.2008, 07.53.091S 147.07.915E Ibalim & Sosanika PNG217” (NHMW, ZSM). 2 males, 4 females “Papua New Guinea Garaina, 800m, vi.2008, 07.53.091S 147.07.915E Ibalim & Sosanika PNG217” (ZSM). 27 males, 18 females “Papua New Guinea: Morobe, Garaina, 770m, vi.2008, 07 52.516S 147.10.427E Ibalim & Sosanika (PNG219)” (NHMW, ZSM). 26 males, 25 females “Papua New Guinea Morobe, Garaina, 800m, 27.vi.2009, (PNG220) 7.52.669S 147.07.196E Ibalim & Sosanika” (NHMW, ZSM). 11 males, 11 females “Papua New Guinea: Morobe, Garaina, 770m, 25.vi.2008, 07 50.859S 147.08.614E Ibalim & Sosanika (PNG222)” (NHMW, ZSM). 2 males, 1 female “Papua New Guinea Morobe, Garaina, 670m, 23vi2008 (PNG223) 7.52.431S 147.10.267E Ibalim & Sosanika (PNG223)” (ZSM). 9 males, 6 females “Papua New Guinea: Morobe, Garaina, 820m, 24.vi.2008, 07.52.287S 147.06.297E Ibalim & Sosanika, (PNG224)” (ZSM). 15 males, 14 females “Papua New Guinea: Morobe, Huon Pen., rd to Kwapsanek, 250m, 31.iii.2006, 06.30.270S (PNG 24) 146.59.581E, Balke & Sagata” (NHMW, ZSM). 4 males, 5 female “Papua New Guinea: Morobe, Huon Pen., rd to Kwapsanek, 250m, 31.iii.2006, 06.30.270S 146.59.581E, Balke & Sagata (PNG 24A)” (ZSM). 2 males, 1 female “Papua New Guinea: Morobe, Huon Pen., rd to Kwapsanek, 460m, 31.iii.2006, 06.32.736S 146.59.616E, Balke & Sagata (PNG 26)”, one male with an additional label “DNA M.Balke 1313” (ZSM). 73 males, 56 females “Papua New Guinea: Morobe, Herzog Mts., Bundun, 700–800m, 2.iv.1994, 06.51.598S 146.37.07E, Balke & Sagata (PNG 27)”, one male with an additional green label “DNA M.Balke 1312” (NHMW, ZSM). 1 male “NEW GUINEA: Morobe Dist., Lae-Bulobo Rd., 28.xii.1964.”, “Stn. No. 123.”, “M.E. Bacchus. B.M. 1965-120” (BMNH). 1 male, 2 females “Papua New Guinea: Morobe, Sattelberg, Zige River, 970m, 20.x.2009, 6 29.233S 147.46.482E, Inaho (11) (PNG211)”, the male with an additional green label “DNA M.Balke 3828” (ZSM). 2 males, 1 female “Papua New Guinea: Morobe, Sattelberg, Zige River, ca 700m, x.2009, 6 29.233S 147.46.482E, Inaho (12a) (PNG212)”, one of the males with an additional green label “DNA M.Balke 3823” (ZSM).

###### Description.

*Body size and form*: Beetle medium-sized, rarely small: TL-H 3.3–4.1 mm, TL 3.6–4.5 mm, MW 1.75–2.1 mm (holotype: TL-H 3.9 mm, TL 4.35 mm, MW 2.05 mm), with oblong-oval habitus.

*Colouration*: Brown to dark brown, usually with reddish pronotum and head. Head reddish to brown, sometimes darker posterior eyes; pronotum reddish to brown, often broader or narrower darker area on disc; elytra brown to dark brown, sometimes with reddish sutural lines; head appendages and legs proximally reddish, legs distally darker, reddish brown to brown (Fig. 4). Teneral specimen paler.

*Surface sculpture*: Shiny dorsally, with fine, sparse punctation and weakly impressed microreticulation. Head with relatively fine and sparse punctation (spaces between punctures 2–3 times size of punctures); diameter of punctures almost equal to or smaller than diameter of cells of microreticulation. Pronotum and elytra with much finer and sparser punctation than on head, often inconspicuous on elytra. Pronotum and elytra with weakly impressed microreticulation. Head with microreticulation slightly stronger. Metaventrite, metacoxae, and abdominal ventrites distinctly microreticulate. Metacoxal plates with longitudinal strioles and weak transverse wrinkles; abdominal ventrites with strioles. Venter with extremely inconspicuous punctation, more evident on metacoxal plates and two last abdominal ventrites.

*Structures*: Pronotum with narrow lateral bead. Base of prosternum and neck of prosternal process with distinct ridge, rounded anteriorly. Blade of prosternal process lanceolate, relatively narrow, slightly convex, with distinct bead and few setae laterally. Abdominal ventrite 6 very slightly truncate.

**Figure 10.**
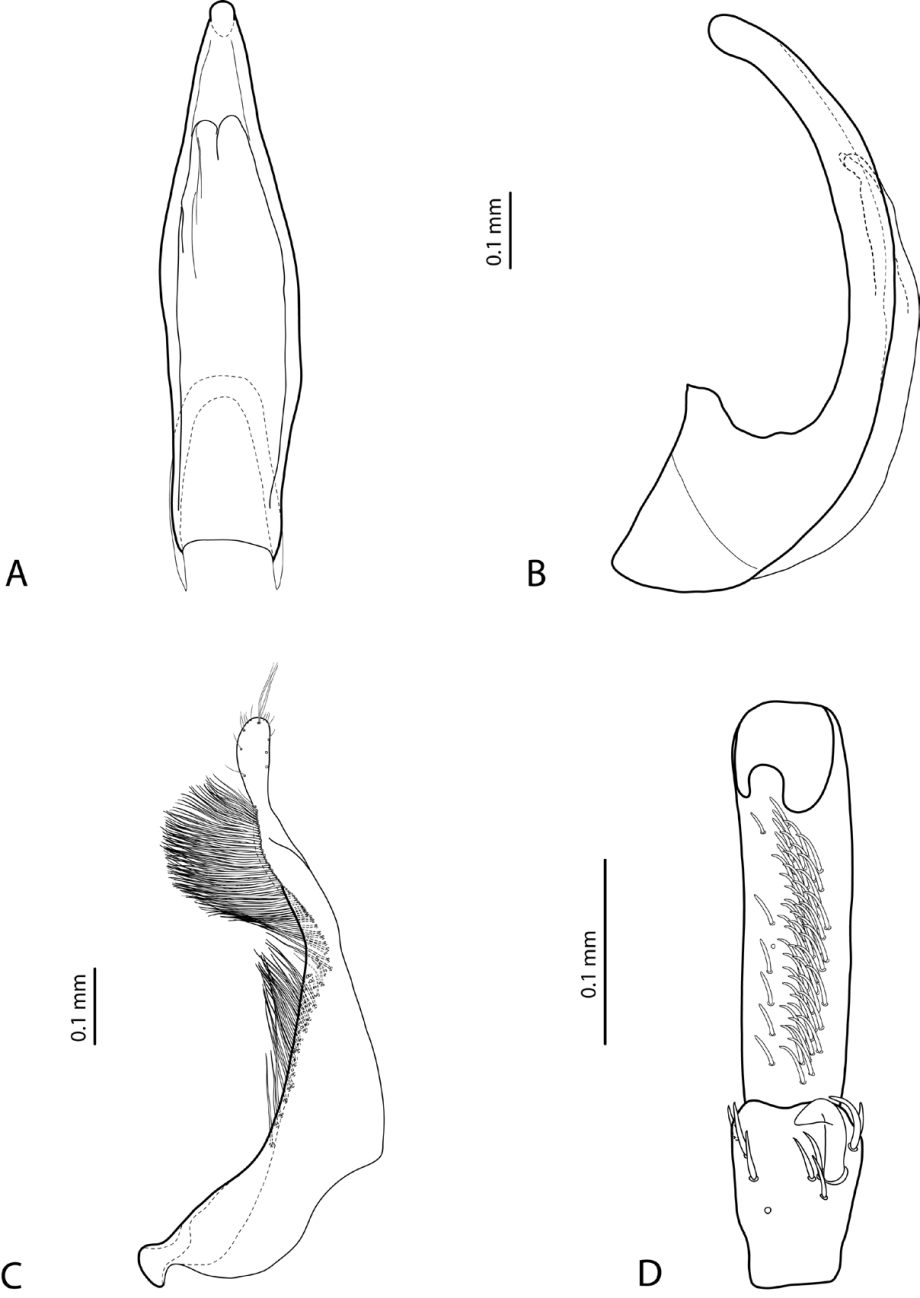
*Exocelina morobensis* sp. nov. **A** median lobe in ventral view **B** median lobe in lateral view **C** paramere in external view **D** male protarsomeres 4–5 in ventral view.

*Male*: Protarsomere 4 with with large, thick, strongly curved anterolateral hook-like seta. Protarsomere 5 ventrally with anterior band of ca. 50 and posterior row of seven relatively long setae (Fig. 10D). Abdominal ventrite 6 with 5–7 lateral striae on each side. Median lobe evenly curved, rather thin, lateral margins thickened proximally; in lateral view, apex elongate, slightly thickened and rounded; in ventral view, median lobe broad proximally and distinctly narrowed in distal half, apex bluntly pointed (Fig. 10A, B). Paramere slightly concave on dorsal side, its subdistal part with dense, strong setae, proximal setae weaker, less distinct (Fig. 10C).

*Female*: Without evident differences in external morphology from males, except for not modified protarsi and abdominal ventrite 6 without striae.

###### Affinities.

From the species co-occurring in the same area (*E. brahminensis* Shaverdo, Hendrich & Balke, 2012, *E. damantiensis*, and *E. garaina* Shaverdo & Balke, 2016), *E. morobensis* sp. nov. can be distinguished by its size, colouration, narrow pronotal bead, and shape and setation of the median lobe and paramere.

###### Distribution.

**Figure 11.**
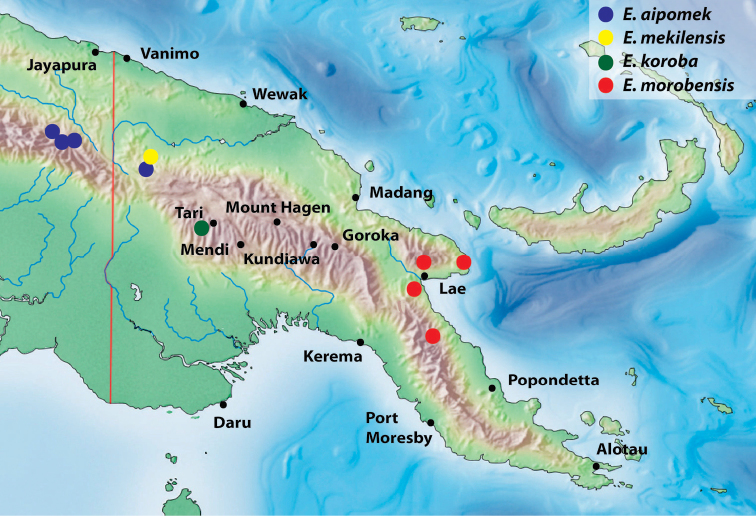
Map of the eastern part of New Guinea showing distribution of the species of the monotypic groups.

Papua New Guinea: Morobe Province (Fig. 11).

###### Etymology.

The species is named after Morobe Province, the only province of PNG where the species has been found. The name is an adjective in the nominative singular.

### Other groups

#### *Exocelina bacchusi* group

The representatives of this group are characterised by fine to coarse dorsal punctation; pronotum with distinct lateral bead; median lobe of aedeagus without setation, simple, broadly pointed; apexes of ventral sclerites of median lobe almost equal; paramere evenly tapering to apex, proximal setae often longer and more distinct that subdistal.

##### 5. Exocelina akameku

Taxon classificationAnimaliaColeopteraDytiscidae

Shaverdo & Balke sp. nov.

941A80EC-FECC-53C9-A730-A5A8FB08B04E

http://zoobank.org/76FBDA94-6B7D-4CCB-8549-88841C285583

Figs 13 , 19

###### Type locality.

Papua New Guinea: Madang Province, Akameku - Brahmin, Bismarck Range, 05°49.89'S, 145°24.49'E, 750 m a.s.l.

###### Type material.

*Holotype*: male “Papua New Guinea: Madang, Akameku - Brahmin, Bismarck Range, 750m, 25.xi.2006, 05.49.892S 145.24.491E, Balke & Kinibel (PNG 113)” (ZSM).

###### Description.

*Body size and form*: Beetle small: TL-H 3.35 mm, TL 3.8 mm, MW 1.8 mm, with oblong-oval habitus.

*Colouration*: Dark brown, with reddish pronotal sides and head anteriorly. Head reddish brown, paler anteriorly; pronotum dark brown on disc, with reddish sides; elytra dark brown, with weakly indicated reddish sutural lines; head appendages and legs proximally yellowish, legs distally darker, reddish brown (Fig. 13).

*Surface sculpture*: Shiny dorsally, with weak and sparse punctation and weakly impressed microreticulation. Head with fine and sparse punctation (spaces between punctures 2–3 times size of punctures); diameter of punctures equal to or smaller than diameter of cells of microreticulation. Pronotum with much finer and sparser punctation than on head, very inconspicuous. Punctation on elytra invisible. Pronotum and elytra with weakly impressed microreticulation; head with microreticulation slightly stronger. Metaventrite, metacoxae, and abdominal ventrites distinctly microreticulate. Metacoxal plates with longitudinal strioles and weak transverse wrinkles; abdominal ventrites with strioles. Punctation on venter invisible; inconspicuous on two last abdominal ventrites.

*Structures*: Pronotum with narrow lateral bead. Base of prosternum and neck of prosternal process with distinct ridge, very slightly rounded anteriorly. Blade of prosternal process lanceolate, relatively narrow, slightly convex, with distinct bead and few setae laterally. Abdominal ventrite 6 slightly truncate.

*Male*: Protarsomere 4 with large, thick, strongly curved anterolateral hook-like seta. Protarsomere 5 ventrally with anterior band of more than 30 and posterior row of 7 relatively long setae (Fig. 19D). Abdominal ventrite 6 with 7–8 lateral striae on each side. Median lobe short, robust, evenly tapering to slightly pointed apex in lateral and ventral views; apex slightly sinuate in lateral view (Fig. 19A, B). Paramere as in Fig. 19C.

*Female*: Unknown.

###### Affinities.

From the species co-occurring in the same area (from *E. danae*, *E. ekari*, *E. broschii*, and *E. ullrichi* groups), *E. akameku* sp. nov. can be distinguished by its size, dorsal punctation, and shape and setation of its median lobe and paramere. For the affinities within the group, see the “Key”.

###### Distribution.

Papua New Guinea: Madang Province, Bismarck Range (Fig. 25).

###### Etymology.

The species is named after Akameku Village. The name is a noun in the nominative singular standing in apposition.

##### 6. Exocelina bacchusi

Taxon classificationAnimaliaColeopteraDytiscidae

(Balke, 1998)

5D8909D7-AF75-5338-AA64-C5DC20DBF7A0

Figs 16 , 22 , 24

Copelatus (Papuadytes) bacchusi Balke, 1998: 326; Nilsson 2001: 76 (catalogue).
Papuadytes bacchusi (Balke, 1998): Nilsson and Fery 2006: 56 (comb. nov.).
Exocelina bacchusi (Balke, 1998): Nilsson 2007: 33 (comb. nov.).
Exocelina bacchusi MB1521: Toussaint et al. 2015: supplementary figs S1, S2, tab. S3, and information S5, S6.
Exocelina undescribed sp. MB0257: Toussaint et al. 2014: supplementary figs 1–4, tab. 2; Toussaint et al. 2015: supplementary figs S1, S2, tab. S3, and information S5, S6.

###### Type locality.

Papua New Guinea: Madang Province, Finisterre Range, Damanti, 05°53'26.5"S, 145°57'50.6"E, 1180 m a.s.l.

###### Type material studied.

*Holotype*: male “Stn. No. 39”, “NEW GUINEA: Madang Dist., Finisterre Mts. Damanti 3,550 ft. 2–11.x.1964.”, “M.E. Bacchus. B.M. 1965-120”, “HOLOTYPUS” [red], “Copelatusbacchusi Balke des. 1997” [red] (BMNH). *Paratypes*: 1 male with the same label as the holotype and additionally with red label “Paratypus Copelatusbacchusi Balke des. 1997” (BMNH). 2 males “Stn. No. 49”, “NEW GUINEA: Madang Dist., Finisterre Mts. Budemu c. 4000 ft. 15–24.x.1964.”, “M.E. Bacchus. B.M. 1965-120” (BMNH, NHMW). 4 females “Stn. No. 74”, “NEW GUINEA: Madang Dist., Finisterre Mts. Budemu c. 4000 ft. 15–24.x.1964.”, “M.E. Bacchus. B.M. 1965-120” (BMNH, NHMW). Note: in the original description (Balke, 1998: 326), the paratypes with the same label as the holotype and from locality “Stn. No. 49” were erroneously indicated as females.

###### Additional material.

**Madang**: 2 males “Papua New Guinea: Madang, Simbai area, 1200m, 11.iii.2007, 05.13.333S 144.37.611E, Kinibel (PNG 153) (ZSM). 15 males, 6 females “Papua New Guinea: Madang, Simbai-Mombeen, 1100m, 11.iii.2007, 05.12.876S 144.41.759E, Kinibel (PNG 154), one male with an additional green label “DNA M.Balke 3318” (NHMW, ZSM). 1 male, 14 females “Papua New Guinea: Madang, Keki-Sewan, Adalbert [sic!] Mts., 700m, 30.xi.2006, nr 04.41.802S 145.25.460E, Binatang Boys (PNG 120)” (ZSM). 4 males, 4 females “Papua New Guinea: Madang, Adalbert [sic!] Mts., creek nr Keki, 790m, 28.xi.1994, 04.42.300S 145.25.089E, Binatang Boys leg. (PNG 53a)” (NHMW, ZSM). 2 males, 5 females “Papua New Guinea: Madang, Adalbert [sic!] Mts., Keki, 850m, 4.v.2006, nr 04.42.300S 145.25.089E, Manaono leg. (PNG 52)” (ZSM). 83 males, 84 females “Papua New Guinea: Eastern Highlands, Akameku - Brahmin, Bismarck Range, 1200m, 24.xi.2006, nr 05.52.754S 145.23.209E, Balke & Kinibel (PNG 110)”, one of them with an additional green label “DNA M.Balke 1521” (NHMW, ZSM). 7 males, 3 femlaes “Papua New Guinea: Eastern Highlands, Akameku - Brahmin, Bismarck Range, 1500m, 24.xi.2006, 05.51.964S 145.23.604E, Balke & Kinibel (PNG 111)” (ZSM). 3 females “Papua New Guinea: Eastern Highlands, Akameku - Brahmin, Bismarck Range, 800m, 24.xi.2006, 05.50.021S 145.24.664E, Balke & Kinibel (PNG 112)” (ZSM). 1 male, 1 female “Papua New Guinea: Madang, Akameku - Brahmin, Bismarck Range, 750m, 25.xi.2006, 05.49.892S 145.24.491E, Balke & Kinibel (PNG 113)” (ZSM). 1 male “Ibisca Niugini, PNG 26–28.x.2012 Mount Wilhelm 1200m”, “-5,720873833 145,2694702 FIT-MW1200-P-1/8-d02 / Plot 16 / P1608 Vial 18767” (ZSM). 1 male “Ibisca Niugini, PNG 26–28.x.2012 Mount Wilhelm 1200m”, “-5,720873833 145,2694702 FIT-MW1200-T-1/8-d02 / Plot 20 / P1640 Vial 18781” (ZSM). 3 females “Ibisca Niugini, PNG 26–28.x.2012 Mount Wilhelm 1200m”, “-5,720873833 145,2694702 FIT-MW1200-P-1/8-d02 / Plot 16 / P1608 Vial 18767” (ZSM). 1 female “Ibisca Niugini, PNG 26–28.x.2012 Mount Wilhelm 1200m”, “-5,720873833 145,2694702 FIT-MW1200-O-1/8-d02 / Plot 15 / P1600 Vial 18763” (ZSM). 1 female “Ibisca Niugini, PNG 26–28.x.2012 Mount Wilhelm 1200m”, “-5,720873833 145,2694702 FIT-MW1200-K-1/8-d02 / Plot 11 / P1568 Vial 17122” (ZSM). 1 female “Ibisca Niugini, PNG 27–29.x.2012 Mount Wilhelm 1200m”, “-5,720873833 145,2694702 FIT-MW1200-A-2/8-d03 / Plot 1 / P1489 Vial 17237” (ZSM). 1 female “Ibisca Niugini, PNG 27–29.x.2012 Mount Wilhelm 1200m”, “-5,720873833 145,2694702 FIT-MW1200-C-2/8-d03 / Plot 3 / P1505 Vial 17179” (ZSM). 6 females “Ibisca Niugini, PNG 27–29.x.2012 Mount Wilhelm 1200m -5,720873833 145,2694702 MW1200 / P1529 Vial 16853” (ZSM). 1 male, 4 females “Ibisca Niugini, PNG 27–29.x.2012 Mount Wilhelm 1200m -5,720873833 145,2694702 MW1200 / P1553 Vial 09007” (ZSM). 1 male, 4 females “Ibisca Niugini, PNG 27–29.x.2012 Mount Wilhelm 1200m -5,720873833 145,2694702 MW1200 / P1545 Vial 16863” (ZSM). 1 male, 7 females “Ibisca Niugini, PNG 27–29.x.2012 Mount Wilhelm 1200m -5,720873833 145,2694702 MW1200 / P1561 Vial 16873” (ZSM). 1 male “Ibisca Niugini, PNG 27–29.x.2012 Mount Wilhelm 1200m”, “-5,720873833 145,2694702 FIT-MW1200-A-2/8-d03 / Plot 1 / P1489 Vial 17237” (ZSM). 7 females “Ibisca Niugini, PNG 27–29.x.2012 Mount Wilhelm 1200m”, “-5,720873833 145,2694702 FIT-MW1200-E-2/8-d03 / Plot 5 / P1521 Vial 17210” (ZSM). 1 female “Ibisca Niugini, PNG 28–30.x.2012 Mount Wilhelm 1200m -5,720873833 145,2694702 MW1200 / P1569 Vial 17302” (ZSM). 3 females “Ibisca Niugini, PNG 28–30.x.2012 Mount Wilhelm 1200m -5,720873833 145,2694702 MW1200 / P1601 Vial 17313” (ZSM). 11 females “Ibisca Niugini, PNG 28–30.x.2012 Mount Wilhelm 1200m -5,720873833 145,2694702 MW1200 / P1595 Vial 18799” (ZSM). 1 female “Ibisca Niugini, PNG 28–30.x.2012 Mount Wilhelm 1200m -5,720873833 145,2694702 MW1200 / P1593 Vial 17462” (ZSM). 2 males “Ibisca Niugini, PNG 28–30.x.2012 Mount Wilhelm 1200m -5,720873833 145,2694702 MW1200 / P1577 Vial 18802” (ZSM). 1 male “Ibisca Niugini, PNG 28–30.x.2012 Mount Wilhelm 1200m -5,720873833 145,2694702 MW1200 / P1625 Vial 18821” (ZSM). 1 female “Ibisca Niugini, PNG 28–30.x.2012 Mount Wilhelm 1200m -5,720873833 145,2694702 MW1200 / P1633 Vial 18848” (ZSM). 1 female “Ibisca Niugini, PNG 28–30.x.2012 Mount Wilhelm 1200m -5,720873833 145,2694702 MW1200 / P1617 Vial 18813” (ZSM). 1 female “Ibisca Niugini, PNG 28–30.x.2012 Mount Wilhelm 1200m -5,720873833 145,2694702 MW1200 / P1601 Vial 17313” (ZSM). 2 females “Ibisca Niugini, PNG 28–30.x.2012 Mount Wilhelm 1200m -5,720873833 145,2694702 MW1200 / P1585 Vial 18825” (ZSM). 1 male, 13 females “Ibisca Niugini, PNG 28–30.x.2012 Mount Wilhelm 1200m -5,720873833 145,2694702 MW1200 / P1609 Vial 18855” (ZSM). 1 female “Ibisca Niugini, PNG 29–31.x.2012 Mount Wilhelm 1200m”, “-5,720873833 145,2694702 FIT-MW1200-D-3/8-d05 / Plot 4 / P1514 Vial 16946” (ZSM). 1 female “Ibisca Niugini, PNG 29–31.x.2012 Mount Wilhelm 1200m”, “-5,720873833 145,2694702 FIT-MW1200-F-3/8-d05 / Plot 6 / P1530 Vial 16936” (ZSM). 1 female “Ibisca Niugini, PNG 29–31.x.2012 Mount Wilhelm 1200m”, “-5,720873833 145,2694702 FIT-MW1200-A-3/8-d05 / Plot 1 / P1490 Vial 16931” (ZSM). 2 females “Ibisca Niugini, PNG 29–31.x.2012 Mount Wilhelm 1200m”, “-5,720873833 145,2694702 FIT-MW1200-H-3/8-d05 / Plot 8 / P1546 Vial 17249” (ZSM). 1 female “Ibisca Niugini, PNG 29–31.x.2012 Mount Wilhelm 1200m”, “-5,720873833 145,2694702 FIT-MW1200-C-3/8-d05 / Plot 3 / P1506 Vial 17235” (ZSM). 1 female “Ibisca Niugini, PNG 29–31.x.2012 Mount Wilhelm 1200m”, “-5,720873833 145,2694702 FIT-MW1200-J-3/8-d05 / Plot 10 / P1562 Vial 16881” (ZSM). 2 females “Ibisca Niugini, PNG 30.x.–1.xi.2012 Mount Wilhelm 1200m -5,720873833 145,2694702 MW1200 / P1578 Vial 17625” (ZSM). 4 females “Ibisca Niugini, PNG 30.x.–1.xi.2012 Mount Wilhelm 1200m -5,720873833 145,2694702 MW1200 / P1570 Vial 17585” (ZSM). 1 female “Ibisca Niugini, PNG 30.x.–1.xi.2012 Mount Wilhelm 1200m -5,720873833 145,2694702 MW1200 / P1634 Vial 17605” (ZSM). 1 female “Ibisca Niugini, PNG 31.x.–2.xi.2012 Mount Wilhelm 1200m -5,720873833 145,2694702 MW1200 / P1547 Vial 17303” (ZSM). 1 female “Ibisca Niugini, PNG 31.x.–2.xi.2012 Mount Wilhelm 1200m -5,720873833 145,2694702 MW1200 / P1515 Vial 17326” (ZSM). 1 female “Ibisca Niugini, PNG 31.x.–2.xi.2012 Mount Wilhelm 1200m -5,720873833 145,2694702 MW1200 / P1491 Vial 17331” (ZSM). 1 female “Ibisca Niugini, PNG 31.x.–2.xi.2012 Mount Wilhelm 1200m -5,720873833 145,2694702 MW1200 / P1531 Vial 17347” (ZSM). 1 female “Ibisca Niugini, PNG 31.x.–2.xi.2012 Mount Wilhelm 1200m”, “-5,720873833 145,2694702 FIT-MW1200-E-4/8-d07 / Plot 5 / P1523 Vial 17348” (ZSM). 2 females “Ibisca Niugini, PNG 1–3.xi.2012 Mount Wilhelm 1200m -5,720873833 145,2694702 MW1200 / P1579 Vial 18787” (ZSM). 8 females “Ibisca Niugini, PNG 1–3.xi.2012 Mount Wilhelm 1200m -5,720873833 145,2694702 MW1200 / P1643 Vial 18794” (ZSM). 1 female “Ibisca Niugini, PNG 1–3.xi.2012 Mount Wilhelm 1200m -5,720873833 145,2694702 MW1200 / P1595 Vial 18799” (ZSM). 3 females “Ibisca Niugini, PNG 1–3.xi.2012 Mount Wilhelm 1200m -5,720873833 145,2694702 MW1200 / P1571 Vial 16947” (ZSM). 1 female “Ibisca Niugini, PNG 1–3.xi.2012 Mount Wilhelm 1200m -5,720873833 145,2694702 MW1200 / P1635 Vial 16968” (ZSM). 1 female “Ibisca Niugini, PNG 1–3.xi.2012 Mount Wilhelm 1200m”, “-5,720873833 145,2694702 FIT-MW1200-S-4/8-d08 / Plot 19 / P1635 Vial 16968-CODYTI” (ZSM). 1 male, 10 females “Ibisca Niugini, PNG 1–3.xi.2012 Mount Wilhelm 1200m -5,720873833 145,2694702 MW1200 / P1611 Vial 16950” (ZSM). 2 females “Ibisca Niugini, PNG 1–3.xi.2012 Mount Wilhelm 1200m -5,720873833 145,2694702 MW1200 / P1611 Vial 16950” (ZSM). 2 females “Ibisca Niugini, PNG 1–3.xi.2012 Mount Wilhelm 1200m -5,720873833 145,2694702 MW1200 / P1635 Vial 16968” (ZSM). 1 female “Ibisca Niugini, PNG 2–4.xi.2012 Mount Wilhelm 1200m -5,720873833 145,2694702 MW1200 / P1516 Vial 17287” (ZSM). 2 females “Ibisca Niugini, PNG 2–4.xi.2012 Mount Wilhelm 1200m -5,720873833 145,2694702 MW1200 / P1548 Vial 17297” (ZSM). 2 females “Ibisca Niugini, PNG 2–4.xi.2012 Mount Wilhelm 1200m -5,720873833 145,2694702 MW1200 / P1532 Vial 17314” (ZSM). 1 female “Ibisca Niugini, PNG 2–4.xi.2012 Mount Wilhelm 1200m -5,720873833 145,2694702 MW1200 / P1492 Vial 17355” (ZSM). 1 male, 2 females “Ibisca Niugini, PNG 2–4.xi.2012 Mount Wilhelm 1200m -5,720873833 145,2694702 MW1200 / P1564 Vial 16857” (ZSM). 1 female “Ibisca Niugini, PNG 2–4.xi.2012 Mount Wilhelm 1200m”, “-5,72090292 145,2714691 FIT-MW1200-C-5/8-d09 / Plot 3 / P1508 Vial 14052-CODYTI” (ZSM). 1 female “Ibisca Niugini, PNG 2–4.xi.2012 Mount Wilhelm 1200m”, “-5,72090292 145,2714691 FIT-MW1200-I-5/8-d09 / Plot 9 / P1556 Vial 17374” (ZSM). 1 female “Ibisca Niugini, PNG 2–4.xi.2012 Mount Wilhelm 1200m -5,720873833 145,2694702”, “FIT-MW1200-E-5/8-d09 / Plot 5 / P1524 Vial 16861-CODYTI” (ZSM). 1 female “Ibisca Niugini, PNG 3–5.xi.2012 Mount Wilhelm 1200m -5,720873833 145,2694702 MW1200 / P1636 Vial 17324” (ZSM). 2 males “Ibisca Niugini, PNG 3–5.xi.2012 Mount Wilhelm 1200m -5,720873833 145,2694702 MW1200 / P1612 Vial 17292” (ZSM). 1 male “Ibisca Niugini, PNG 3–5.xi.2012 Mount Wilhelm 1200m -5,720873833 145,2694702 MW1200 / P1572 Vial 18837” (ZSM). 1 female “Ibisca Niugini, PNG 3–5.xi.2012 Mount Wilhelm 1200m”, “-5,720873833 145,2694702 FIT-MW1200-O-5/8-d10 / Plot 15 / P1604 Vial 17325” (ZSM). 1 male “Ibisca Niugini, PNG 4–6.xi.2012 Mount Wilhelm 1200m”, “-5,720873833 145,2694702 FIT-MW1200-F-6/8-d11 / Plot 6 / P1533 Vial 17257” (ZSM). 1 male “Ibisca Niugini, PNG 5–7.xi.2012 Mount Wilhelm 1200m”, “-5,720873833 145,2694702 FIT-MW1200-K-6/8-d12 / Plot 11 / P1573 Vial 17082” (ZSM). 1 female “Ibisca Niugini, PNG 8–10.xi.2012 Mount Wilhelm 1200m -5,721022129 145,2703094 MW1200 / P1503 Vial 16995” (ZSM). 1 female “Ibisca Niugini, PNG 8–10.xi.2012 Mount Wilhelm 1200m -5,720873833 145,2694702 MW1200 / P1527 Vial 16879” (ZSM). 1 female “Ibisca Niugini, PNG 8–10.xi.2012 Mount Wilhelm 1200m -5,720873833 145,2694702 MW1200 / P1503 Vial 16995” (ZSM). 1 male, 1 female “Ibisca Niugini, PNG 9–11.xi.2012 Mount Wilhelm 1200m,” “-5,720873833 145,2694702 FIT-MW1200-T-8/8-d16 / Plot 20 / P1647 Vial 17039-CODYTI” (ZSM). 4 females “Ibisca Niugini, PNG 9–11.xi.2012 Mount Wilhelm 1200m,” “-5,720873833 145,2694702 FIT-MW1200-P-8/8-d16 / Plot 16 / P1615 Vial 17012-CODYTI” (ZSM). 1 female “Ibisca Niugini, PNG 9–11.xi.2012 Mount Wilhelm 1200m,” “-5,720873833 145,2694702 FIT-MW1200-M-8/8-d16 / Plot 13 / P1591 Vial 17035-CODYTI” (ZSM). 1 female “Ibisca Niugini, PNG 1–3.xi.2012 Mount Wilhelm 1700m -5,79269238 145,235611 MW1700 / P1961 Vial 06629” (ZSM). **Eastern Highlands**: 3 male, 3 females “Papua New Guinea: Eastern Highlands, Bena Bridge, 1400m, 8.xii.2007, 06.10.781S 145.26.034E, Balke & Sagata (PNG 164)” (ZSM). **Simbu/Eastern Highlands**: 2 males “Papua New Guinea: Simbu/EHPr. Crater Mountain, Sera - Herowana, Wara Pima, 900 m, 15IX2002, Balke & Sagata (PNG 011)” (ZSM). 16 males “Papua New Guinea: Crater Mountain, Sera - Herowana, upper Oh River, 1200 m, 15IX2002, Balke & Sagata (PNG 012)” (NHMW, ZSM). 2 males, 4 females “Papua New Guinea: Crater Mountain, Sera - Herowana, Jau river, 1100m, 15IX2002, Balke & Sagata (PNG 013)” (ZSM). 12 males “Papua New Guinea: Simbu/EHPr. Crater Mountain, Sera - Herowana, Jau river, 1000 m, 15IX2002, Balke & Sagata (PNG 015)” (NHMW, ZSM). 1 male, 3 females “Papua New Guinea: Simbu / EHP, Crater Mountain, Sera - Herowana, Sima river, 1250m, 15IX2002, Balke & Sagata (PNG 016)” (ZSM). **Simbu**: 5 males “Papua New Guinea: Supa Haia, 1023m, 10.ix.2002, K.Sagata (WB1)” (NHMW, ZSM). 1 male “Papua New Guinea: Crater Mountain, trek Haia - Wara Sera, 600m, 12IX2002, Balke & Sagata, (PNG 003)” (ZSM). 3 males “Papua New Guinea: Crater Mountain, trek Haia - Wara Sera, 500m, 12IX2002, Balke & Sagata, (PNG 005)” (ZSM). 1 male “Papua New Guinea: Crater Mountain, trek Haia - Wara Sera, 500m, 12IX2002, Balke & Sagata, (PNG 006)” (ZSM). 11 males, 14 females “Papua New Guinea: Simbu/EHPr. Crater Mountain, Wara Sera Station, 800 m, 14IX2002, Balke & Sagata (PNG 009)” (NHMW, ZSM). 12 males, 7 females “Papua New Guinea: Simbu/EHPr. Crater Mountain, Wara Sera Station, 800 m, 14IX2002, Balke & Sagata (PNG 10)”, one male with additional labels “257 DNA M Balke” [green], “sp.17 SEM 19” (ZSM). 27 males, 31 females “Papua New Guinea: Crater Mountain, Wara Sera Station, 800 m, 14IX2002, Balke & Sagata (PNG 010)” (NHMW, ZSM). **Morobe**: 62 males, 27 females “PAPUA N.G.: Morobe Prov. E Pindiu, Kobau 24.4.1998, 1400 m, leg. A. Riedel” (NHMW, ZSM). 1 male “Papua New Guinea: Morobe, Pindiu, Sulemana, 850 m, 15.x.2009, 06.25.169S 147.32.11E, Inaho (08) (PNG 208)”, “DNA M.Balke 3825” [green] (ZSM). 2 males “PNG: Huon Peninsula, Morobe Prov., Yus conservation area [5°53'54"S, 146°48'15"E], 1398m, 24.viii.2010, Bega”, “DNA M. Balke 6531” [green text], “DNA M. Balke 6532” [green text] (ZSM). 6 females “PNG: Huon Peninsula, Morobe Prov., Yus conservation area (Y7), 1398m, 24.viii.2010, Huon, Bega” (ZSM). **Gulf**: 9 males, 8 females “Papua New Guinea: Gulf, Marawaka, nr Ande, 1000m, 10.xi.2006, 07.03.598S 145.44.375E, Balke & Kinibel (PNG 89)” (NHMW, ZSM).

###### Females of doubtful identity.

**Simbu**: 27 females “Papua New Guinea: Supa Haia, 1023m, 10.ix.2002, K.Sagata (WB1)” (ZSM); these females are a mixture of three species: *E. bacchusi*, *E. warasera*, and *E. haia*. 5 females “Papua New Guinea: Crater Mountain, trek Haia - Wara Sera, 600m, 12IX2002, Balke & Sagata, (PNG 003)” (ZSM); these females are a mixture of two species: *E. bacchusi* and *E. warasera*. 3 females “Papua New Guinea: Crater Mountain, trek Haia - Wara Sera, 500m, 12IX2002, Balke & Sagata, (PNG 005)” (ZSM); these females are a mixture of three species: *E. bacchusi*, *E. warasera*, and *E. haia*. **Simbu/Eastern Highlands**: 3 females “Papua New Guinea: Simbu/EHPr. Crater Mountain, Sera – Herowana, Wara Pima, 900 m, 15IX2002, Balke & Sagata (PNG 011)” (ZSM). 20 females “Papua New Guinea: Crater Mountain, Sera - Herowana, upper Oh River, 1200 m, 15IX2002, Balke & Sagata (PNG 012)” (ZSM). 4 females “Papua New Guinea: Simbu/EHPr. Crater Mountain, Sera - Herowana, Jau river, 1000 m, 15IX2002, Balke & Sagata (PNG 015)” (ZSM). These females are a mixture of two species: *E. bacchusi* and *E. warasera*.

###### Diagnosis.

For complete description, see Balke (1998: 326). Beetle small to medium-sized: TL-H 3.05–3.9 mm, oblong-oval; dorsally uniformly reddish to dark brown or with paler head and sides of pronotum; shiny, with very fine to distinct punctation and usually weakly impressed microreticulation; pronotum with distinct lateral bead (Fig. 16); male protarsomere 4 with anterolateral seta very long and thin, evenly curved, smaller than more laterally situated large seta; male protarsomere 5 ventrally with anterior band of more than 50 and posterior row of 8 relatively long setae (Fig. 22D); median lobe simple, evenly attenuated to broadly pointed apex in lateral and ventral views; paramere very slightly concave on dorsal side and with long, dense, thin setae, situated along dorsal margin; proximal setae longer that subdistal, more distinct (Fig. 22A–C).

###### Variability.

The species shows variability in size, colouration, how strongly impressed dorsal punctation and, more seldom, microreticulation, and slightly in shape of the apex of the median lobe (Fig. 24).

###### Affinities.

From the species co-occurring in the same area (*E. craterensis* Shaverdo & Balke, 2014, *E. damantiensis* (Balke, 1998), *E. hintelmannae* (Shaverdo, Sagata & Balke, 2005), *E. sima*, *E. kobau* sp. nov. and two species of the *E. larsoni* group), *E. bacchusi* can be distinguished by its reddish dorsal colouration and shape and setation of the median lobe and paramere. The most similar (in body size and form and colouration) to *E. bacchusi* are *E. warasera* sp. nov. and *E. haia* sp. nov., which occur with it. Only males of these species can be clearly separated by shape and setation of the median lobe and paramere; and therefore, dorsal setae of the paramere are important: in *E. bacchusi*, proximal setae longer that subdistal, more distinct. For the affinities within the group, see the “Key”.

###### Distribution.

Papua New Guinea: Madang, Simbu, Eastern Highlands, Morobe and Gulf Provinces (Fig. 25). This is one of the most abundant species in the region.

##### 6a. Exocelina bacchusi herzogensis

Taxon classificationAnimaliaColeopteraDytiscidae

Shaverdo & Balke ssp. nov.

BB967394-B81D-56C7-A135-25136A9E8C64

http://zoobank.org/7E252D3A-EF0B-4BDA-BC3E-C90D754BDB18

Figs 17 , 23

Exocelina undescribed sp. MB1383: Toussaint et al. 2014: supplementary figs 1–4, tab. 2; Toussaint et al. 2015: supplementary figs S1, S2, tab. S3, and information S5, S6.

###### Type locality.

Papua New Guinea: Central Province, Woitape, 08°33.17'S, 147°15.48'E, 1500 m a.s.l.

###### Type material.

*Holotype*: male “Papua New Guinea: Central, Woitape, 1500m, i.2008, [08°] 33.178S 147.15.481E, Posman (PNG 167)”, “DNA M.Balke 3401” [green] (ZSM). *Paratypes*: 1 male, 2 females with the same labels as the holotype (NHMW, ZSM). 1 male “Papua New Guinea: Morobe, Wagau, Herzog Mts., 1150m, 19.xi.2006, 06.51.067S 146.48.068E, Balke & Kinibel (PNG 102)”, “DNA M.Balke 1383” [green] (ZSM).

###### Description.

*Body size and form*: Beetle small: TL-H 3.4–3.6 mm, TL 3.8–4.0 mm, MW 1.85–2.0 mm (holotype: TL-H 3.6 mm, TL 4.0 mm, MW 2.0 mm), with oblong-oval habitus.

*Colouration*: Yellow reddish to brown. Head reddish brown to brown, dark brown posterior to eyes. Pronotum yellowish reddish, with small dark area on disc or brown, with paler sides. Elytra yellow reddish to brown. Head appendages and legs proximally yellowish, legs distally darker, reddish brown (Fig. 17). Teneral specimen yellowish.

*Surface sculpture*: Shiny dorsally, with very fine punctation and weakly impressed microreticulation. Elytral punctation and microreticulation finer then in nominotypical subspecies. Elytral punctation usually invisible.

*Structures*: Pronotum with lateral bead. Base of prosternum and neck of prosternal process with distinct ridge, slightly rounded anteriorly. Blade of prosternal process lanceolate, relatively broad, slightly convex, with distinct bead and few setae laterally. Abdominal ventrite 6 slightly truncate.

*Male*: Protarsomere 4 with anterolateral seta rather long and thing, evenly curved, smaller than more laterally situated large seta. Protarsomere 5 ventrally with anterior band of more than 60 and posterior row of ten relatively long setae (Fig. 23D). Abdominal ventrite 6 with 6–8 lateral striae on each side. Median lobe simple, evenly tapering towards apex in lateral and ventral views; in lateral view, apex elongate, thin, with slightly enlarged, rounded tip (Fig. 23A, B). Paramere as in Fig. 23C.

*Female*: Without evident differences in external morphology from males, except for not modified protarsi and abdominal ventrite 6 without striae.

###### Variability.

Colouration of the specimens from Woitape distinctly paler, yellowish; the specimen form Wagau much darker, brown.

###### Affinities.

From the nominotypical subspecies, it can be distinguished by shinier dorsal surface, shorter setae of male protarsomere 4, and by apex of the median lone elongate, thinner, with slightly enlarged tip. The further study is necessary to confirm the status of this taxon, which seems to replace the nominotypical subspecies in the Papuan Peninsula.

###### Distribution.

Papua New Guinea: Morobe and Central Provinces (Fig. 25).

###### Etymology.

The subspecies is named after Herzog Mts., where the subspecies was the first time discovered. The name is an adjective in the nominative singular.

##### 7. Exocelina erteldi

Taxon classificationAnimaliaColeopteraDytiscidae

(Balke, 1998)

807019FE-D654-5375-94C7-6E6B28173514

Figs 14 , 20

Copelatus (Papuadytes) erteldi Balke, 1998: 330; Nilsson 2001: 76 (catalogue).
Papuadytes erteldi (Balke, 1998): Nilsson and Fery 2006: 56 (comb. nov.).
Exocelina erteldi (Balke, 1998): Nilsson 2007: 33 (comb. nov.).

###### Type locality.

Indonesia: Papua Province: Pegunungan Bintang Regency, Borme, ca. 04°24'S, 140°25'E, 1200 m a.s.l.

###### Type material studied.

*Holotype*: male “IRIAN JAYA Zentralmassive 140°25'E 04°24'S”, “14./17.8.1992 Borme, 1900m leg. Balke (11)”, “Copelatuserteldi Balke des. 1997” [red], “HOLOTYPUS” [red] (NHMW). *Paratypes*: 1 female with the same label as the holotype and additionally with a red label “Paratypus Copelatuserteldi Balke des. 1997” (NHMW). 54 males, 35 females “IRIAN JAYA Zentralmassive 140°25'E 04°24'S”, “Borme, 1800m 16.8.1992 leg. Balke (12, 12 A)”, one of the males with two additional labels “M.Balke 3273” [green] and “M.Balke 6404 DNA” [green text], another male with an additional green label “M.Balke 3273” (CGW, NHMW). Note: in the original description (Balke 1998), number of specimens of the locality (12, 12 A) is erroneously given as “43 males, 46 females”. 1 female “12./18.8.1992 Borme, 100m leg. Balke (7)”, “Paratypus Copelatuserteldi Balke des. 1997”, this paratyte does not belong to species of *E. erteldi* but to *E. bifida*Shaverdo et al. 2012.

###### Additional material.

1 male “IRIAN JAYA Zentralmassive 140°25'E 04°24'S”, “Borme, 1800m 16.8.1992 leg. Balke (12, 12 A)”, “Paratypus Copelatusfume Balke des. 1997” [red] (NHMW).

###### Diagnosis.

For complete description, see Balke (1998: 330). Beetle small (TL-H 3.45–3.75 mm), oblong-oval; brown to piceous, usually with paler pronotal sides; dorsally more or less shiny, with fine but conspicuous punctation and weakly impressed microreticulation; pronotum with distinct lateral bead (Fig. 14); male protarsomere 4 with anterolateral seta thin, weakly curved, smaller than more laterally situated large seta; male protarsomere 5 ventrally with anterior band of ca. 70 and posterior row of 6 relatively long setae (Fig. 20D); median lobe in lateral view evenly attenuated to elongate, thin apex, which slightly pointed in ventral view; paramere slightly concave on dorsal side and with distinct, long, dense, uniform setae, situated along dorsal margin (Fig. 20A–C).

###### Affinities.

The species can be distinguished from the species co-occurring in the same area (*E. ascendens*, *E. aipomek*, *E. takime*, the *E. ekari* group: *E. eme* Shaverdo and *E. bifida*, the *E. danae* group: *E. damantiensis* and *E. danae*, the *E. okbapensis* group: *E. ketembang*, *E. talaki*, and *E. okbapensis*, and all species of the *E. aipo* group) by body size and colouration, presence of pronotal bead, fine but conspicuous dorsal punctation, and shape and setation of its median lobe, paramere, and male protarsomere 4. For the affinities within the group, see the “Key”.

###### Distribution.

Indonesia: Papua Province: Pegunungan Bintang Regency, Borme (Fig. 25). The species is known only from the type material.

##### 8. Exocelina oiwa

Taxon classificationAnimaliaColeopteraDytiscidae

Shaverdo & Balke sp. nov.

EEE0CC5E-6EB0-541A-BDE7-DE47B43DD574

http://zoobank.org/DE65A0BD-5EAE-457F-99EA-1AA47E8B1916

Figs 12 , 18

###### Type locality.

Papua New Guinea: Morobe Province, Aseki, Oiwa (a village about 100 km to the west of Bulolo), 7°18'00.0"S, 146°14'00.0"E, 1600–1700 m a.s.l.

###### Type material.

*Holotype*: male “PAPUA N. G.: Morobe Prov. Aseki, Oiwa, 1600–1700 m, 11.–12.3.1998 leg. A. Riedel” (NHMW). *Paratypes*: 4 males, 2 females with the same labels as the holotype, one male and one female additionally with labels “SEM 19” (NHMW, ZSM).

###### Description.

*Body size and form*: Beetle small: TL-H 3.3–3.5 mm, TL 3.7–3.95 mm, MW 1.85–1.95 mm (holotype: TL-H 3.5 mm, TL 3.95 mm, MW 1.95 mm), with oblong-oval habitus.

**Figures 12–17.**
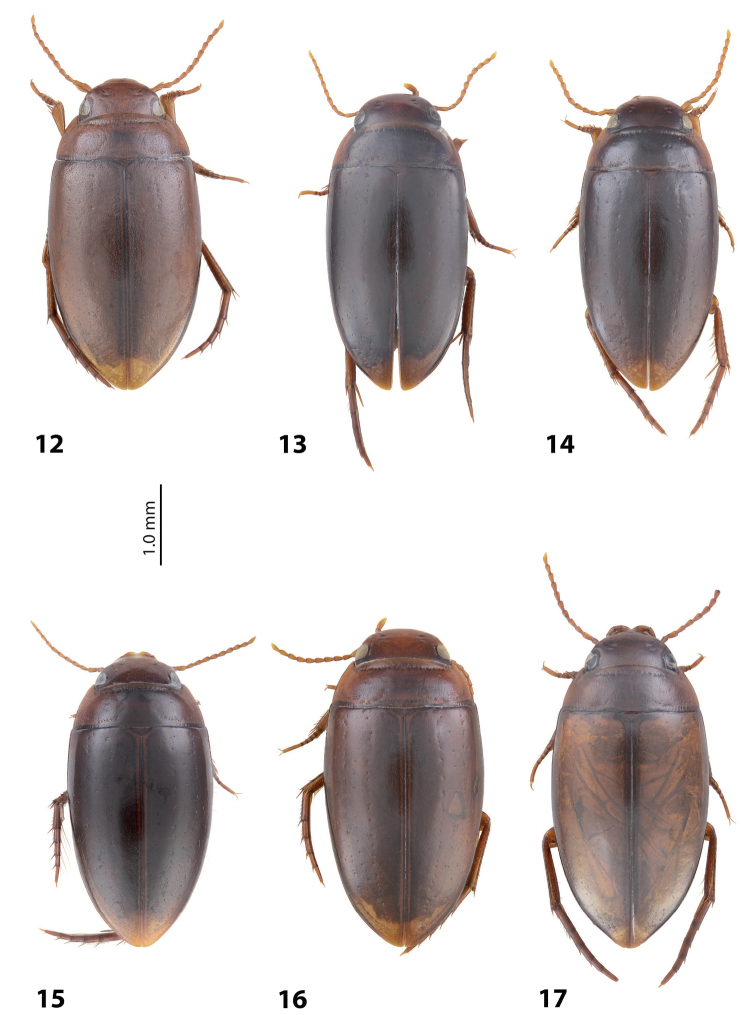
Habitus and colouration **12***Exocelina oiwa* sp. nov. **13***E. akameku* sp. nov. **14***E. erteldi* (Balke, 1998) **15***E. oksibilensis* sp. nov. **16***E. bacchusi* (Balke, 1998) **17***E. bacchusi herzogensis* sp. nov.

*Colouration*: Fast uniformly reddish brown. Head reddish brown, darker posterior eyes. Pronotum reddish brown, slightly darker on disc. Elytra reddish brown, sometimes slightly darker than pronotum. Head appendages and legs proximally yellowish, legs distally darker, reddish (Fig. 12).

*Surface sculpture*: Submatt dorsally, with strong and dense punctation and strongly impressed microreticulation. Head with dense and coarse punctation (spaces between punctures 0–1 times size of punctures); diameter of punctures equal to or larger than diameter of cells of microreticulation. Pronotum and elytra with finer and sparser punctation than on head, very distinct, more even on elytra. Pronotum and elytra with strongly impressed microreticulation; head with microreticulation stronger. Metaventrite, metacoxae, and abdominal ventrites distinctly microreticulate, but shiny. Metacoxal plates with longitudinal strioles and weak transverse wrinkles; abdominal ventrites with strioles. Punctation on venter weak; more distinct on two last abdominal ventrites.

*Structures*: Pronotum with lateral bead. Base of prosternum and neck of prosternal process with distinct ridge, slightly rounded anteriorly. Blade of prosternal process lanceolate, relatively broad, slightly convex, with distinct bead and few setae laterally. Abdominal ventrite 6 broadly rounded or slightly truncate.

**Figures 18, 19.**
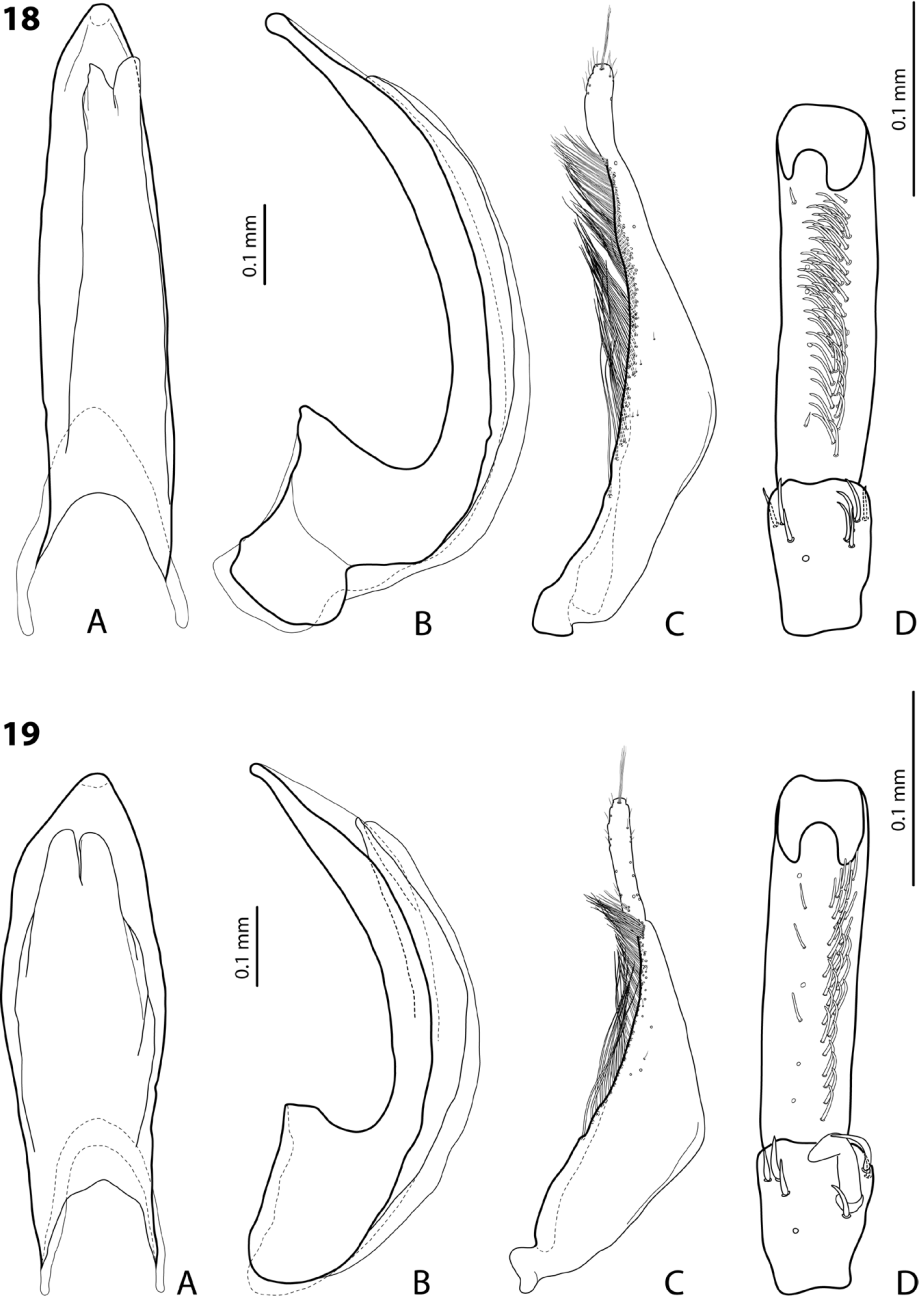
**18***Exocelina oiwa* sp. nov. **19***E. akameku* sp. nov. **A** median lobe in ventral view **B** median lobe in lateral view **C** paramere in external view **D** male protarsomeres 4–5 in ventral view.

*Male*: Protarsomere 4 with anterolateral seta rather long and thin, evenly curved, equal to laterally situated large seta. Protarsomere 5 ventrally with anterior band of ca. 60 and posterior row of five relatively long setae (Fig. 18D). Abdominal ventrite 6 with 8–9 lateral striae on each side. Median lobe short, evenly tapering to apex in lateral and ventral views, very tip of apex thickened dorsally (Fig. 18A, B). Paramere as in Fig. 18D.

*Female*: Without evident differences in external morphology from males, except for not modified protarsi and abdominal ventrite 6 without striae.

###### Affinities.

From the species co-occurring in the same area (from *E. danae*, *E. ekari*, *E. broschii*, and *E. ullrichi* groups), *E. oiwa* sp. nov. can be distinguished by its size, dorsal punctation and colouration, shape and setation of its median lobe and paramere, and thin, evenly curved anterolateral seta of the protarsomere 4. The species is especially similar to *E. aseki* sp. nov., from which it can be distinguished by shape of its median lobe. For the affinities within the group, see the “Key”.

###### Distribution.

Papua New Guinea: Morobe Province (Fig. 25).

###### Etymology.

The species is named after Oiwa Village. The name is a noun in the nominative singular standing in apposition.

##### 9. Exocelina oksibilensis

Taxon classificationAnimaliaColeopteraDytiscidae

Shaverdo, Surbakti, Warikar & Balke sp. nov.

6731F430-FEC6-5C59-8809-46C59359CEE5

http://zoobank.org/26C2CDCD-463E-4E23-9D11-225EE70A292C

Figs 15 , 21

###### Type locality.

Indonesia: Papua Province, Pegunungan Bintang Regency, south from Ok Sibil, tributary Digul River 05°03'25.9"S, 140°43'21.1"E, 359 m a.s.l.

###### Type material.

*Holotype*: male “Indonesia: Papua, S Ok Sibil, tributary Digul Riv, 359m, 9.vi.2018, -5,05718389 140,722535848617, Sumoked (Pap051)” (MZB). *Paratypes*: 4 males, 13 females with the same label as the holotype, 2 males with additional labels “6996” [green text], “7001” [green text] (KSP, MZB, ZSM).

###### Description.

*Body size and form*: Beetle small: TL-H 3.05–3.35 mm, TL 3.5–3.7 mm, MW 1.7–1.85 mm (holotype: TL-H 3.35 mm, TL 3.7 mm, MW 1.85 mm), usually with oval, egg-shaped habitus.

*Colouration*: Reddish brown to brown. Head reddish brown to dark brown, paler anteriorly. Pronotum dark brown on disc and narrower or broader reddish on sides. Elytra reddish brown to dark brown, with reddish sutural lines. Head appendages and legs proximally yellowish, legs distally darker, reddish brown (Fig. 15).

*Surface sculpture*: Shiny dorsally, with fine punctation and weakly impressed microreticulation. Head with fine and sparse punctation (spaces between punctures 2–3 times size of punctures); diameter of punctures equal to or smaller than diameter of cells of microreticulation. Pronotum and elytra with much finer and sparser punctation than on head, sometimes inconspicuous. Pronotum and elytra with weakly impressed microreticulation; head with microreticulation slightly stronger. Metaventrite, metacoxae, and abdominal ventrites distinctly microreticulate. Metacoxal plates with longitudinal strioles and weak transverse wrinkles; abdominal ventrites with strioles. Punctation on venter invisible; inconspicuous on two last abdominal ventrites.

*Structures*: Pronotum with narrow lateral bead. Base of prosternum and neck of prosternal process with distinct ridge, slightly rounded anteriorly. Blade of prosternal process lanceolate, relatively broad, slightly convex, with distinct bead and few setae laterally. Abdominal ventrite 6 slightly truncate.

*Male*: Protarsomere 4 with anterolateral seta rather long and thing, evenly curved, smaller than more laterally situated large seta. Protarsomere 5 ventrally with anterior band of more than 60 and posterior row of 4 relatively long setae (Fig. 21D). Abdominal ventrite 6 with 3–6 lateral striae on each side. Median lobe simple, evenly tapering to broadly pointed apex in lateral and ventral views (Fig. 21A, B). Paramere as in Fig. 21C.

*Female*: Without evident differences in external morphology from males, except for not modified protarsi and abdominal ventrite 6 without striae.

###### Affinities.

The species is very similar to *E. bacchusi* in shape of the median lobe but can be distinguished from it by smaller size and egg-shaped habitus and shorter setae of male protarsomere 4. From the other species co-occurring in the same province (*E. ascendens*, *E. aipomek*, *E. takime*, the *E. ekari* group: *E. eme* Shaverdo and *E. bifida*, the *E. danae* group: *E. damantiensis* and *E. danae*, the *E. okbapensis* group: *E. ketembang*, *E. talaki* and *E. okbapensis*, and all species of the *E. aipo* group), it can be separated by body size and form, presence of pronotal bead, and the shape and setation of its median lobe, paramere, and male protarsomere 4. For the affinities within the group, see the “Key”.

###### Distribution.

Indonesia: Papua Province, Pegunungan Bintang Regency, Ok Sibil area (Fig. 25).

###### Etymology.

The species is named after Ok Sibil River. The name is an adjective in the nominative singular.

##### Key to the species of *Exocelina bacchusi* group

**Table:** 

1	Beetle dorsally submatt, with strong and dense punctation and strongly impressed microreticulation (Fig. 12)	***oiwa* sp. nov.**
–	Beetle dorsally shiny, often with very weak punctation, invisible on elytra, and weakly impressed microreticulation	**2**
2	Anterolateral seta of male protarsomere 4 hook-like, large, strongly curved (Fig. 19D)	***akameku* sp. nov.**
–	Anterolateral seta of male protarsomere 4 thin, long, slightly curved, equal to or smaller than more laterally situated large setae	**3**
3	Median lobe with subparallel sides and short apex in ventral view; in lateral view, apex elongate. Setae of paramere uniform, distinct (Fig. 20)	***erteldi* (Balke, 1998)**
–	Median lobe more or less evenly tapering to apex in ventral and lateral views. Proximal setae of paramere usually longer, sometimes also much stronger, than subdistal	**4**
4	Beetle usually smaller, TL-H 3.05–3.35 mm (Fig. 15), oval, egg-shaped. Anterolateral seta of male protarsomere 4 shorter and thicker (Fig. 21D). Median lobe and paramere as in Fig. 21A–C	***oksibilensis* sp. nov.**
–	Beetle usually larger, TL-H 3.05–3.9 mm (Figs 16, 17), elongate. Anterolateral seta of male protarsomere 4 very long and thing (Fig. 22D). Median lobe and paramere as in Fig. 22A–C	**5**
5	Beetle with stronger elytral microreticulation and more distinct punctation, duller (Fig. 16). Apex of median lobe shorter, thicker (Fig. 22A, B)	***bacchusi* (Balke, 1998)**
–	Beetle with weaker elytral microreticulation and usually invisible elytral punctation, shinier (Fig. 17). Apex of median lobe more elongate, thinner (Fig. 23A, B)	***bacchusiherzogensis* ssp. nov.**

**Figures 20, 21.**
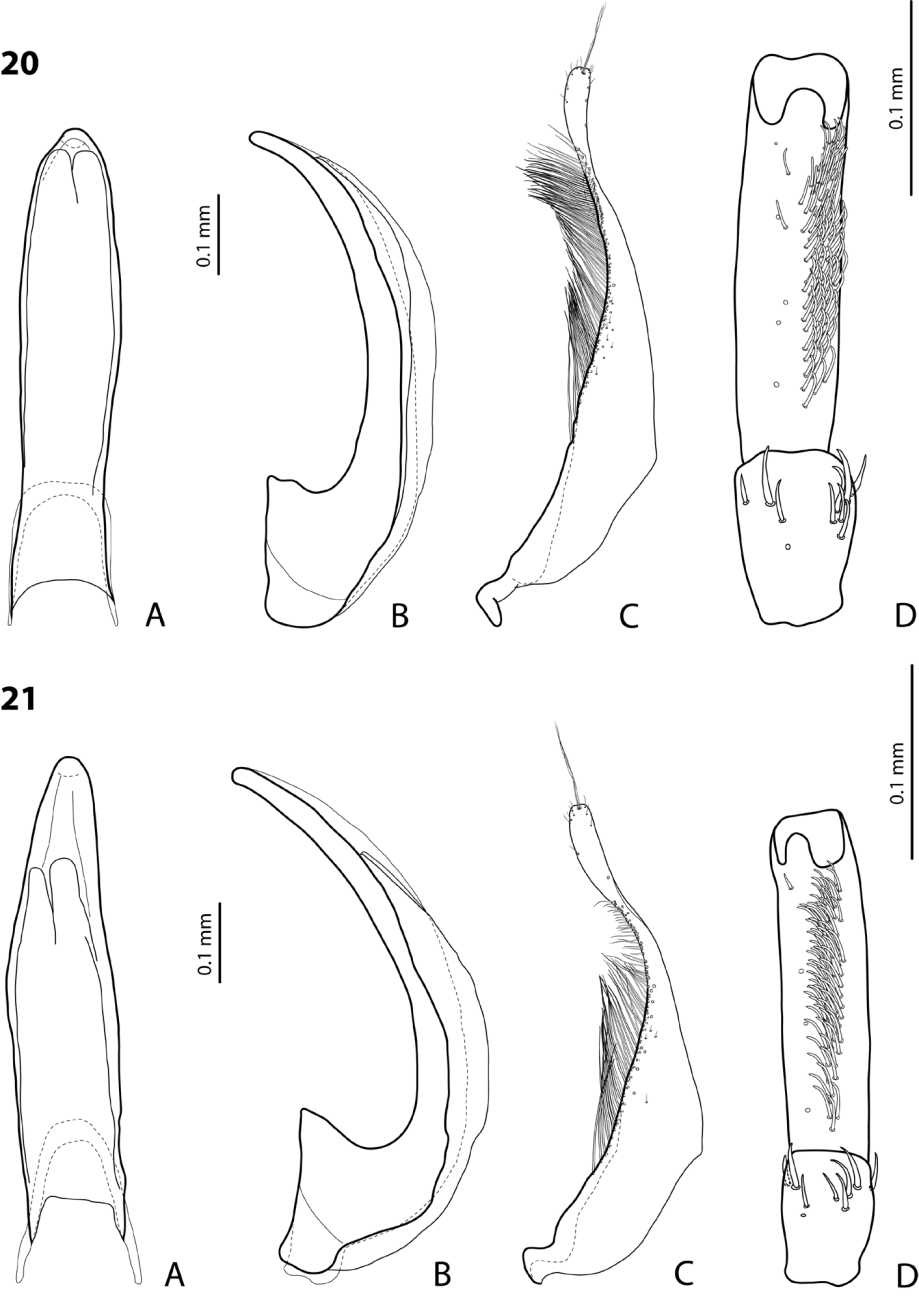
**20***Exocelina erteldi* (Balke, 1998) **21***E. oksibilensis* sp. nov. **A** median lobe in ventral view **B** median lobe in lateral view **C** paramere in external view **D** male protarsomeres 4–5 in ventral view.

**Figures 22, 23.**
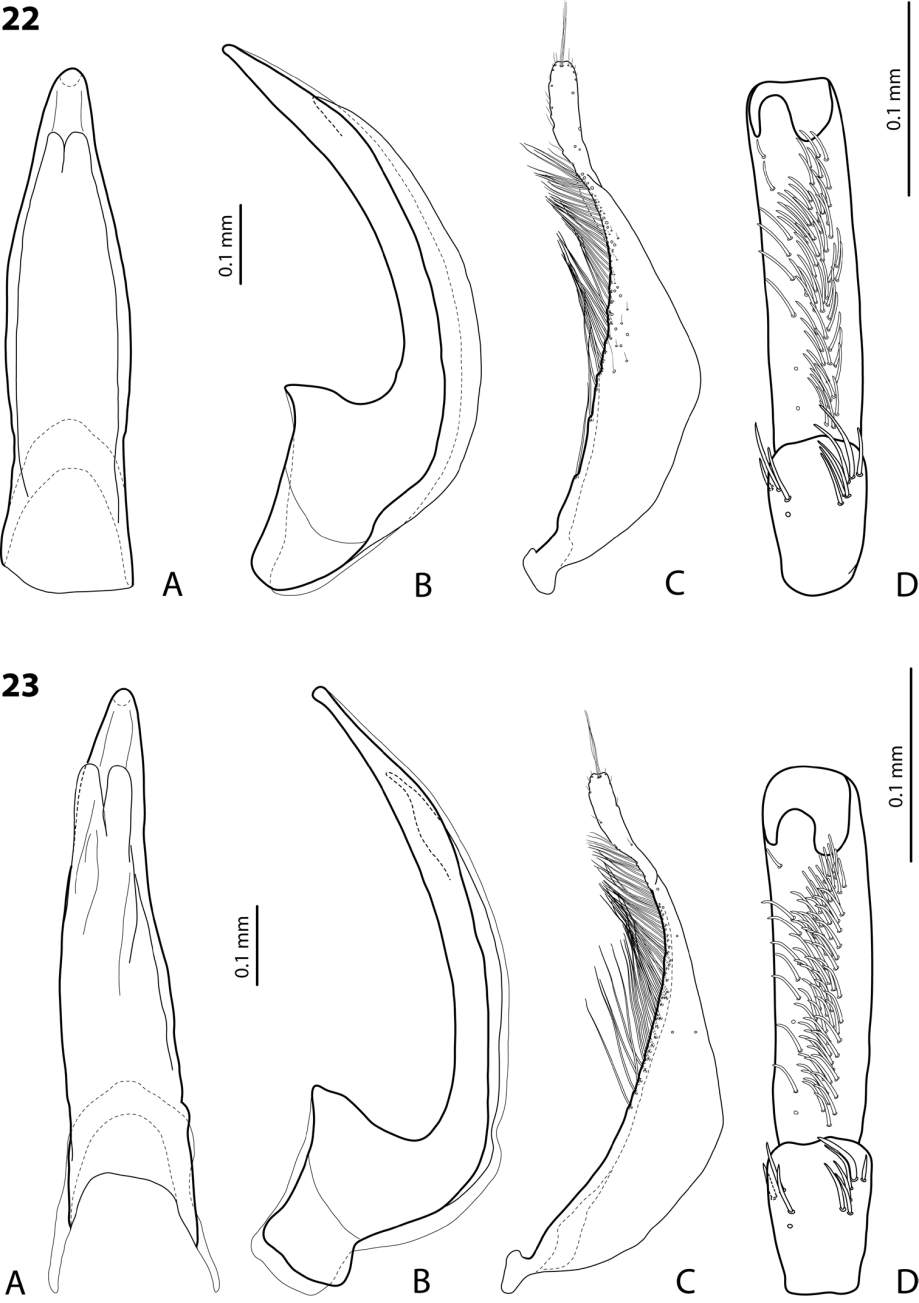
**22***Exocelina bacchusi* (Balke, 1998), paratype (Madang, Damanti) **23***E. bacchusi herzogensis* ssp. nov. **A** median lobe in ventral view **B** median lobe in lateral view **C** paramere in external view **D** male protarsomeres 4–5 in ventral view.

**Figure 24.**
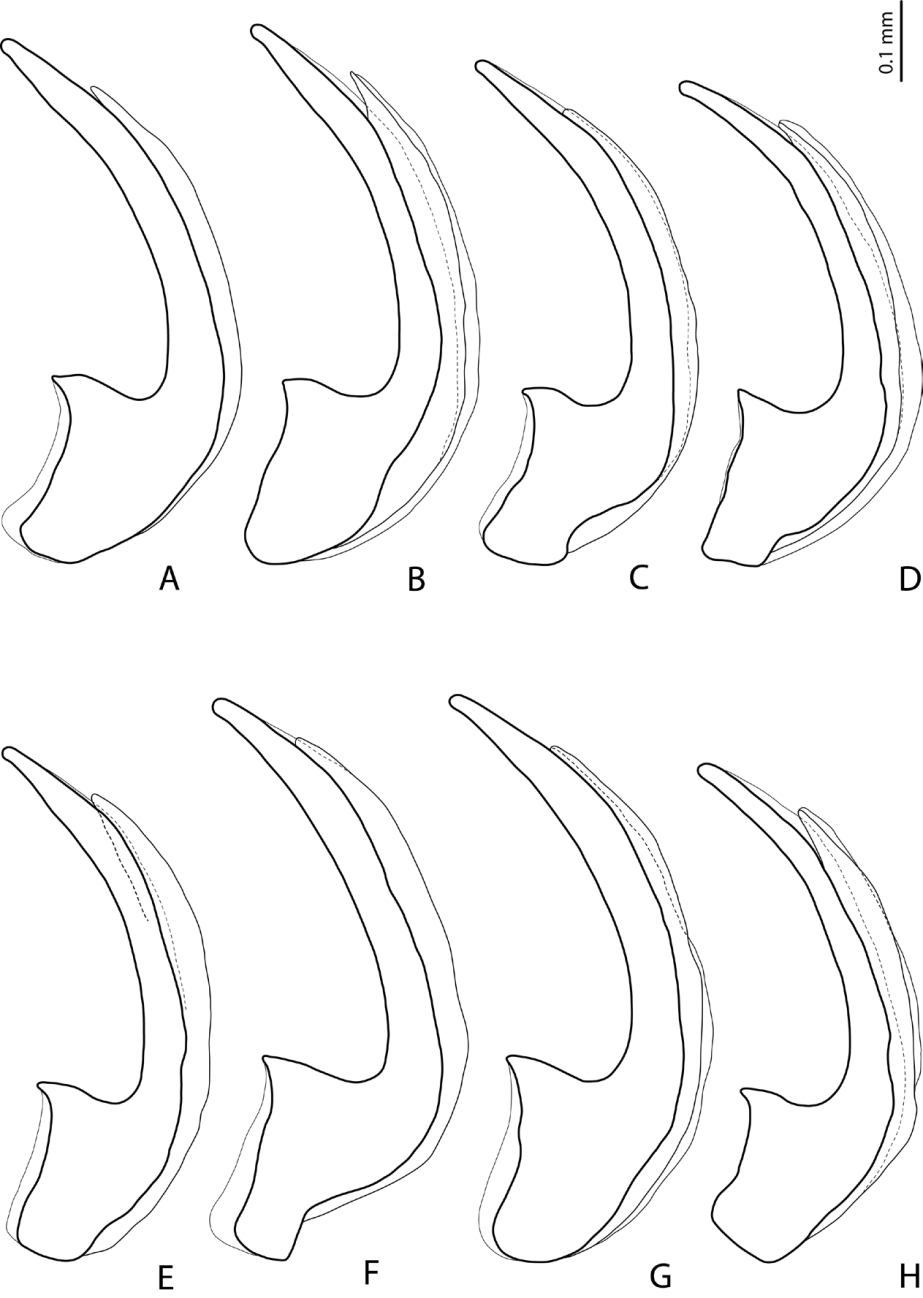
*Exocelina bacchusi* (Balke, 1998), median lobe in lateral view **A** Madang, Adelbet Mt **B** Madang, Bismarck Range **C** Madang, Wilhelm Mt **D** Simbu, Crater Mt **E** EHL, Bena **F** Morobe, Kobau **G** Morobe, Yus **H** Gulf, Marawaka.

**Figure 25.**
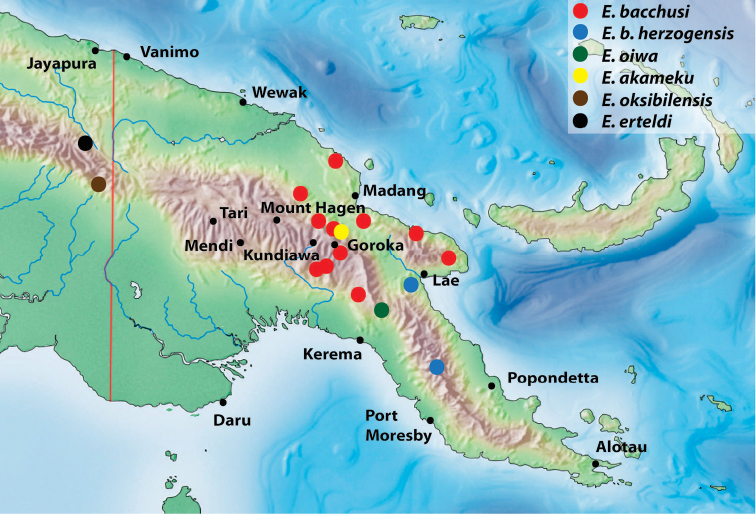
Map of the eastern part of New Guinea showing distribution of the species of the *E. bacchusi* group.

#### *Exocelina jaseminae* group

This group is characterised by fine to coarse dorsal punctation; pronotum with distinct lateral bead; median lobe of aedeagus without setation; in ventral view, with distinctly concave apex forming two apical lobes; in lateral view, apex tip prolongated into characteristic “nose”; apexes of ventral sclerites of median lobe almost equal or slightly unequal; paramere without distinct notch but slightly concave on dorsal side, its subdistal part with dense, strong setae, proximal setae inconspicuous.

##### 10. Exocelina aseki

Taxon classificationAnimaliaColeopteraDytiscidae

Shaverdo & Balke sp. nov.

D998CB1A-1571-59F8-9BA0-272DABEE75AF

http://zoobank.org/6253C250-9E5C-454C-86FD-ED376372D449

Figs 21 , 22–23 , 49

###### Type locality.

Papua New Guinea: Morobe Province, Aseki, Oiwa, ca. 07°21'01.5"S, 146°11'38.4"E, 1600–1700 m a.s.l.

###### Type material.

*Holotype*: male “PAPUA N. G.: Morobe Prov. Aseki, Oiwa, 1600–1700 m, 11.–12.3.1998 leg. A. Riedel”, “SEM 19” (NHMW).

###### Description.

*Body size and form*: Beetle small: TL-H 3.4 mm, TL 3.8 mm, MW 1.8 mm, with oblong-oval habitus.

**Figures 26–29.**
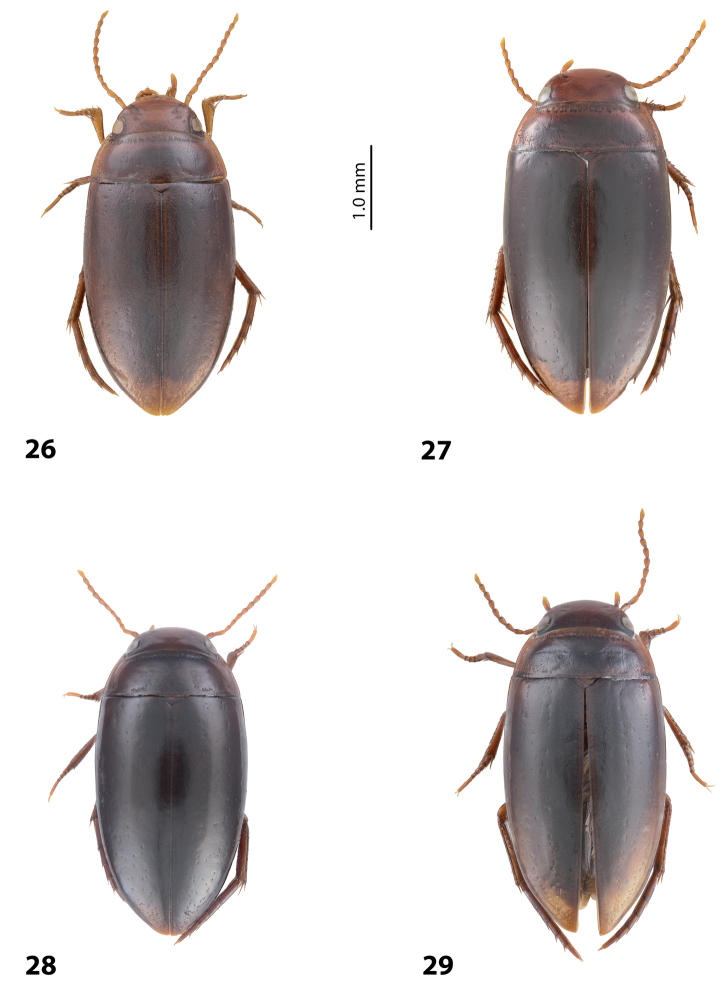
Habitus and colouration **26***Exocelina aseki* sp. nov. **27***E. jaseminae* (Balke, 1998) **28***E. kailaki* sp. nov. **29***E. pseudojaseminae* sp. nov.

*Colouration*: Reddish brown. Head reddish brown. Pronotum reddish brown, dark brown on disc and almost yellowish on lateral sides. Elytra brown, with reddish sutural lines. Head appendages and legs proximally yellowish, legs distally darker, reddish (Fig. 26).

*Surface sculpture*: Submatt dorsally, with strong and dense punctation and strongly impressed microreticulation. Head with dense and coarse punctation (spaces between punctures 0–1 times size of punctures); diameter of punctures equal to or larger than diameter of cells of microreticulation. Pronotum and elytra with finer and sparser punctation than on head, very distinct, more even on elytra. Pronotum and elytra with strongly impressed microreticulation; head with microreticulation stronger. Metaventrite, metacoxae, and abdominal ventrites distinctly microreticulate, but shiny. Metacoxal plates with longitudinal strioles and weak transverse wrinkles; abdominal ventrites with strioles. Punctation on venter weak; more distinct on two last abdominal ventrites.

*Structures*: Pronotum with lateral bead. Base of prosternum and neck of prosternal process with distinct ridge, slightly rounded anteriorly. Blade of prosternal process lanceolate, relatively broad, slightly convex, with distinct bead and few setae laterally. Abdominal ventrite 6 broadly rounded.

**Figures 30, 31.**
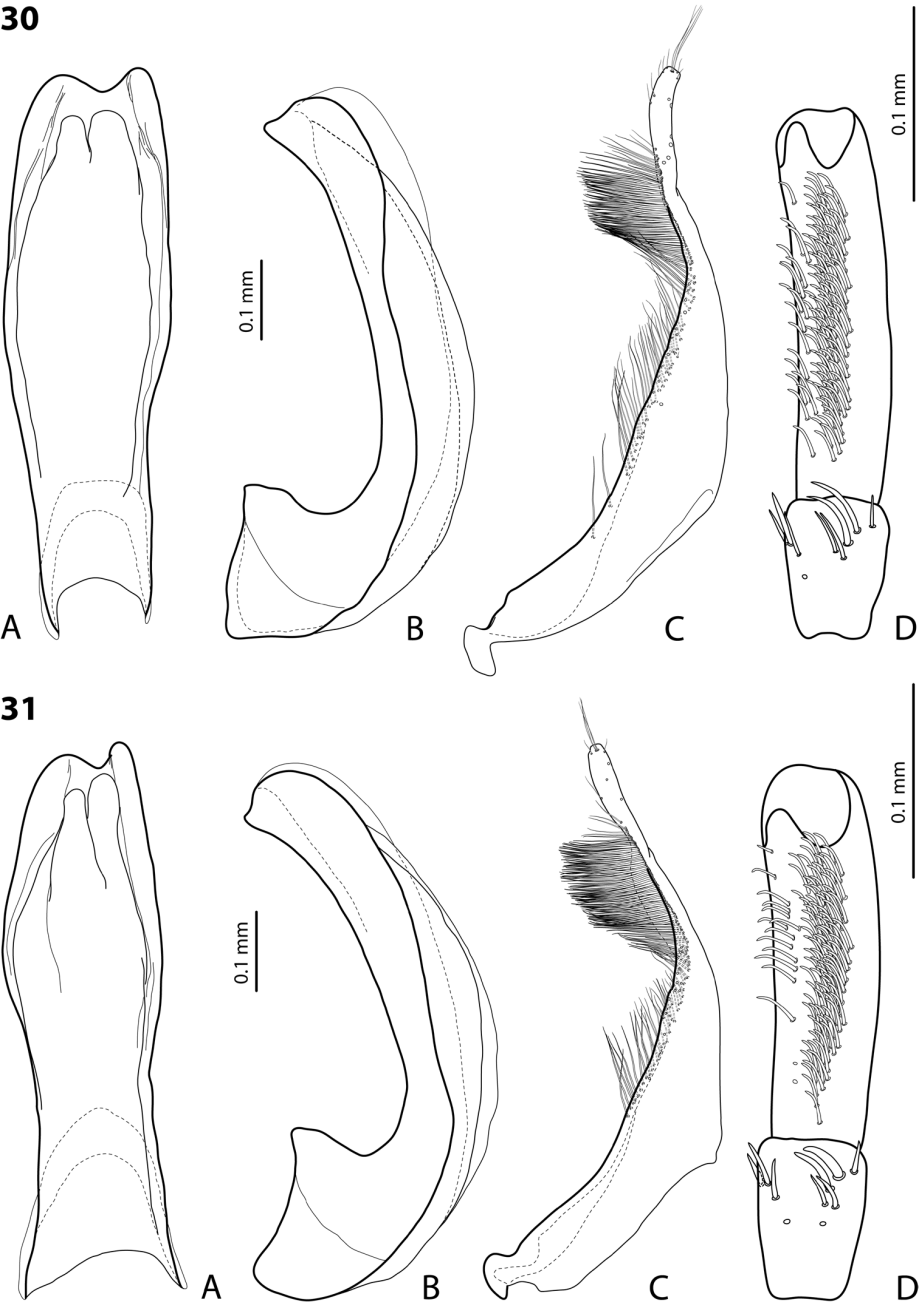
**30***Exocelina aseki* sp. nov. **31***E. jaseminae* (Balke, 1998) **A** median lobe in ventral view **B** median lobe in lateral view **C** paramere in external view **D** male protarsomeres 4–5 in ventral view.

*Male*: Protarsomere 4 with anterolateral seta very long and thin, evenly curved, in size equal to more laterally situated large seta. Protarsomere 5 ventrally with anterior band of ca. 80 and posterior row of ca. 16 relatively long setae, which mixed up medially (Fig. 30D). Abdominal ventrite 6 with 4–6 lateral striae on each side. Median lobe in lateral view with apical lobes distinct but shallow, slightly rounded, “nose” elongate, large (Fig. 30A, B). Paramere as in Fig. 30C.

*Female*: Unknown.

###### Affinities.

From the species co-occurring in the same area (from *E. danae*, *E. ekari*, *E. broschii*, and *E. ullrichi* groups), *E. aseki* sp. nov. can be distinguished by its size, dorsal punctation and colouration, shape and setation of its median lobe and paramere, and thin anterolateral seta of the male protarsomere 4. The species is especially similar to *E. oiwa* sp. nov., from which it can be distinguished by shape of its median lobe. For the affinities within the group, see the “Key”.

###### Distribution.

Papua New Guinea: Morobe Province (Fig. 34).

###### Etymology.

The species is named after Aseki Village. The name is a noun in the nominative singular standing in apposition.

##### 11. Exocelina jaseminae

Taxon classificationAnimaliaColeopteraDytiscidae

(Balke, 1998)

757E69DB-AB55-5E01-AA5E-DD3AA356177C

Figs 27 , 31

Copelatus (Papuadytes) jaseminae Balke, 1998: 331; Nilsson 2001: 77 (catalogue).
Papuadytes jaseminae (Balke, 1998): Nilsson and Fery 2006: 56 (comb. nov.).
Exocelina jaseminae (Balke, 1998): Nilsson 2007: 33 (comb. nov.).
Exocelina jaseminae MB1382: Toussaint et al. 2014: supplementary figs 1–4, tab. 2; Toussaint et al. 2015: supplementary figs S1, S2, tab. S3, and information S5, S6.

###### Type locality.

Papua New Guinea: Morobe Province, Herzog Range, Wagau (Vagau), ca. 06°48'S, 146°48'E, ca. 1300 m a.s.l.

###### Type material studied.

*Holotype*: male “Stn. No. 150”, “NEW GUINEA: Morobe Dist., Herzog Mts., Vagau, C.4,000ft. 4–17.i.1965”, “M. E. Bacchus. B. M. 1965-120”, “HOLOTYPUS” [red], “Copelatusjaseminae sp. nov. Balke des. 1997” [red] (BMNH). *Paratypes*: 2 males, 3 females with the same label as the holotype and additionally with a red label “Paratypus Copelatusjaseminae sp.n. Balke des. 1997”, one of the males with an additional label “measured J. Parkin 85” (BMNH, NHMW). 1 male with the same label as the holotype, the red paratype label missing (BMNH).

###### Additional material.

**Morobe**: 1 male “Stn. No. 149A”, “NEW GUINEA: Morobe Dist., Herzog Mts., Vagau, C.4,000ft. 4–17.i.1965”, “M. E. Bacchus. B. M. 1965-120”, “Paratypus Copelatusmonae sp.n. Balke des. 1997” [red], “Exocelina jaseminae (Balke) det. H.Shaverdo 2014” (BMNH). 2 males “Papua New Guinea: Morobe, Wagau, Herzog Mts., 1150m, 19.xi.2006, 06.51.067S 146.48.068E, Balke & Kinibel (PNG 102)”, one male with an additional green label “DNA M.Balke 1382” (ZSM). 1 male, 2 females “Papua New Guinea: Morobe, Wagau, Herzog Mts., 1150m, 19.xi.2006, 06.51.067S 146.48.068E, Balke & Kinibel (PNG 103)” (ZSM). 2 males “Papua New Guinea: Gulf [sic!], Menyamya, Mt Inji 1700m, 14.xi.2006 nr 07.14.813S 146.01.330E Balke & Kinibel (PNG 96)” (ZSM). 9 males “PAPUA N. G.: Morobe Prov. Aseki, Oiwa, 1600–1700 m, 11.–12.3.1998 leg. A. Riedel” (NHMW). 6 males, 2 females “Papua New Guinea: Gulf [Morobe], Marawaka, Andakombe towards Morobe, 1100m, 12.xi.200, 07.09.766S, 145.46.333E, Balke & Kinibel (PNG 92)” (NHMW, ZSM). **Eastern Highlands**: 38 males, 38 females “Papua New Guinea: Eastern Highlands, Marawaka, Ande, 1700m, 8.xi.2005, 07.01.697S 145.49.807E, Balke & Kinibel (PNG 86)”, one male with an additional green label “DNA M.Balke 1365” (NHMW, ZSM). 49 males, 40 females “Papua New Guinea: Eastern Highlands, Marawaka, Ande, 1700–1800m, 9.xi.2006, 07.01.697S 145.49.807E, Balke & Kinibel (PNG 87)” (NHMW, ZSM).

###### Diagnosis.

For complete description, see Balke (1998: 331). Beetle medium-sized: TL-H 3.55–4.1 mm; oblong-oval; brown to dark brown, with reddish to reddish brown pronotal sides or pronotum and often head; shiny, with very fine, on elytra often almost invisible punctation and weakly impressed microreticulation; pronotum with lateral bead (Fig. 27); male protarsomere 4 with anterolateral seta thin, evenly curved, smaller than or equal to more laterally situated large seta; male protarsomere 5 ventrally with anterior band of more than 60 and posterior row of 13–15 relatively long setae (Fig. 31D); median lobe in lateral view with apical lobes distinctly developed and rounded, “nose” small but distinct; paramere slightly concave on dorsal side and with long, dense, thin setae, situated along dorsal margin distinctly divided to dense and strong subdistal setae and sparser proximal ones, setae in middle short and fine (Fig. 31A–C).

###### Affinities.

In the area of its distribution, *E. jaseminae* co-occurs with numerous species of the *E. ekari*, *E. ullrichi*, *E. broschii*, and *E. danae* groups. From them, this species can be distinguished by its size, dorsal colouration, surface sculpture, simple male antennae, presence of pronotal bead, and mainly by the shape of its median lobe. In its external appearance, *E. jaseminae* is especially similar to *E. monae* (Balke, 1998), from which can be distinguished by the shape of its median lobe. For the affinities within the group, see the “Key”.

###### Distribution.

Papua New Guinea: Eastern Highlands and Morobe Provinces (Fig. 34).

##### 12. Exocelina kailaki

Taxon classificationAnimaliaColeopteraDytiscidae

Shaverdo & Balke sp. nov.

208EA224-2D04-579F-99A6-A6530E9A407F

http://zoobank.org/1C4730A9-D83B-4A8F-B4AB-99591F7E99DD

Figs 28 , 32

Exocelina undescribed sp. MB3409: Toussaint et al. 2014: supplementary figs 1–4, tab. 2; Toussaint et al. 2015: supplementary figs S1, S2, tab. S3, and information S5, S6.

###### Type locality.

Papua New Guinea: Central Province, Kailaki, 09°24.134'S, 147°33.521'E, 827 m a.s.l.

###### Type material.

*Holotype*: male “Papua New Guinea: Central, Moroka area, Kailaki, 827 m, 26.x.2009, 9.24.134S 147.33.521E, Sagata (PNG225)” (ZSM). *Paratypes*: 14 males, 27 females with the same label as the holotype (NHMW, ZSM). 3 males, 5 females “Papua New Guinea Central, Moroka, Kailaki Wareaga, 760m, 27x2009 9.25.424S 147.31.068E Sagata (PNG227)” (ZSM). 1 male “Stn. No. 200B”, “PAPUA: Musgrave River, Sogeri Plateau, Nr. Pt. Moresby 16.iii.1965”, “M.E. Bacchus. B.M. 1965-120” (BMNH). 15 males, 16 females “Papua New Guinea: Central, Myola, 1110m, i.2008, 09 12.630S 147.31.880E, Posman (PNG 177)”, one male with an additional green label “DNA M.Balke 3409” (NHMW, ZSM). 7 males, 6 females “Papua New Guinea: Central, Kokoda Trek, 1390m, i.2008, [09°] 00.338S 147.44.252E, Posman (PNG 173)” (NHMW, ZSM). 1 male “Papua New Guinea: Central, 755m, 28.x.2009 S9.25.47.5 E147.32 59.1, Sagata (PNG229)” (ZSM).

###### Description.

*Body size and form*: Beetle small: TL-H 3.1–3.85 mm, TL 3.45–4.35 mm, MW 1.7–2.05 mm (holotype: TL-H 3.4 mm, TL 3.75 mm, MW 1.85 mm), with oblong-oval habitus.

*Colouration*: Piceous, with paler sides of pronotum and head anteriorly. Head reddish brown to dark brown, paler anteriorly. Pronotum dark brown, to piceous on disc and to reddish on sides. Elytra uniformly dark brown to piceous. Head appendages and legs proximally yellowish to reddish, legs distally darker, reddish brown (Fig. 28). Teneral specimen paler, brown to reddish brown with to yellowish pronotum and head.

*Surface sculpture*: Shiny dorsally, with extremely fine, sparse punctation and weakly impressed microreticulation. Head with fine and sparse punctation (spaces between punctures 2–3 times size of punctures); diameter of punctures smaller than diameter of cells of microreticulation. Pronotum with much finer and sparser punctation than on head, very inconspicuous. Punctation on elytra invisible. Pronotum and elytra with weakly impressed microreticulation; head with microreticulation slightly stronger. Metaventrite, metacoxae, and abdominal ventrites distinctly microreticulate. Metacoxal plates with longitudinal strioles and weak transverse wrinkles. Punctation on venter invisible; inconspicuous on two last abdominal ventrites.

*Structures*: Pronotum with narrow lateral bead. Base of prosternum and neck of prosternal process with distinct ridge, slightly rounded anteriorly. Blade of prosternal process lanceolate, relatively broad, slightly convex, with distinct bead and few setae laterally. Abdominal ventrite 6 truncate.

**Figures 32, 33.**
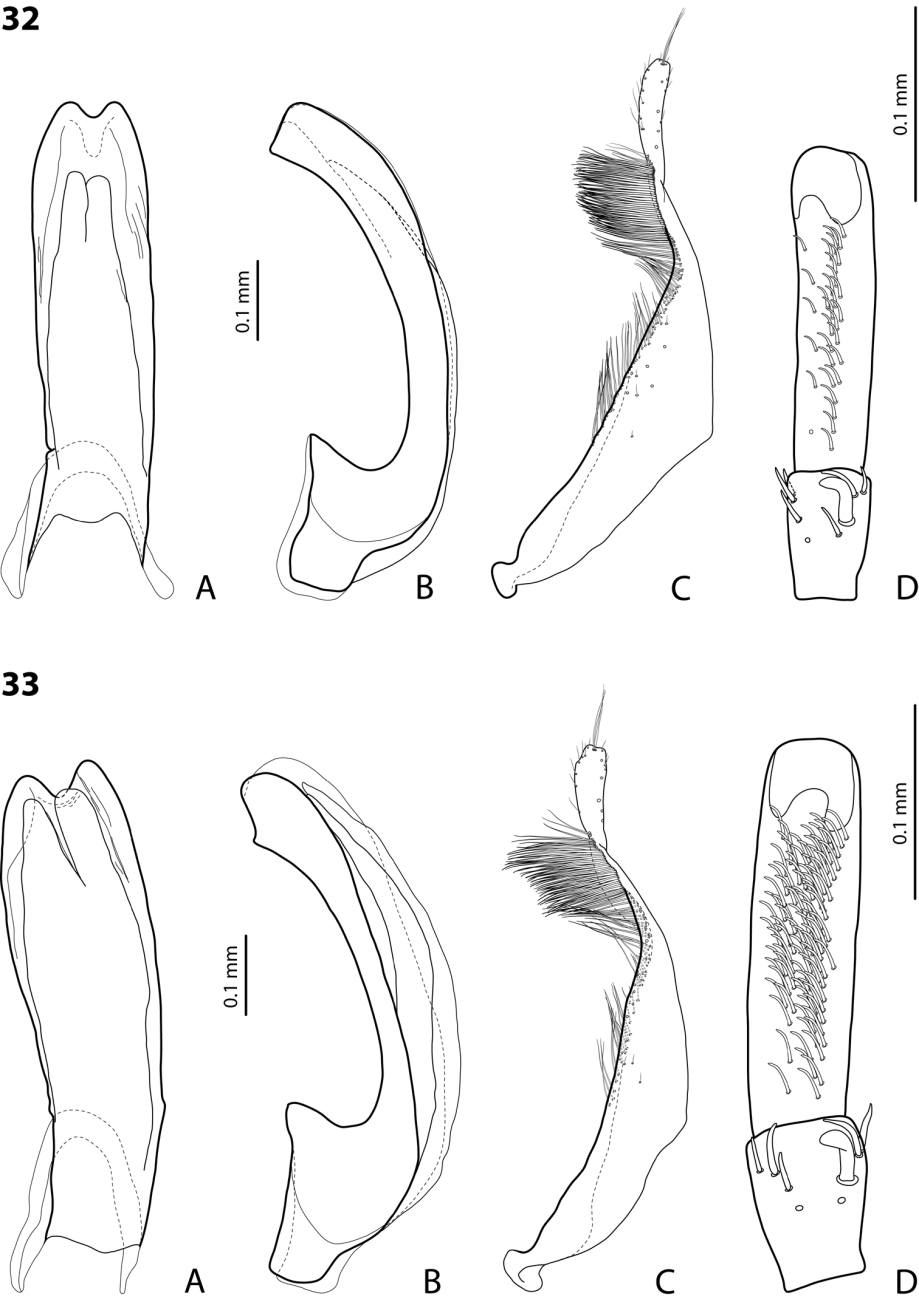
**32***Exocelina kailaki* sp. nov. **33***E. pseudojaseminae* sp. nov. **A** median lobe in ventral view **B** median lobe in lateral view **C** paramere in external view **D** male protarsomeres 4–5 in ventral view.

*Male*: Protarsomere 4 with large, thick, strongly curved anterolateral hook-like seta. Protarsomere 5 ventrally with anterior narrow band of 26 setae and posterior row of six relatively long setae (Fig. 32D). Abdominal ventrites 1–3 with long strioles, abdominal ventrites 4–6 without strioles or with 1–2 small lateral strioles on each side. Median lobe with apical lobes weakly developed, not rounded, truncate in lateral view, “nose” usually indistinct (Fig. 32A, B). Paramere as in Fig. 32C.

*Female*: Without evident differences in external morphology from males, except for not modified protarsi. Abdominal ventrites 1–2 with strioles, abdominal ventrites 3–6 without strioles.

###### Variability.

Shape of apex of the medial lobe varies. In some specimens, especially from Myola, it is not clearly truncate in lateral view but very slightly concave and, due to that, the “nose” is more distinct.

###### Affinities.

*Exocellina kailaki* sp. nov. can be distinguished from the species of the *E. danae* group, *E. nomax* and *E. pulchella* sp. nov., co-occurring in the same area by its size, dorsal colouration and punctation, and shape and setation of its median lobe and paramere. For the affinities within the group, see the “Key”.

###### Distribution.

**Figure 34.**
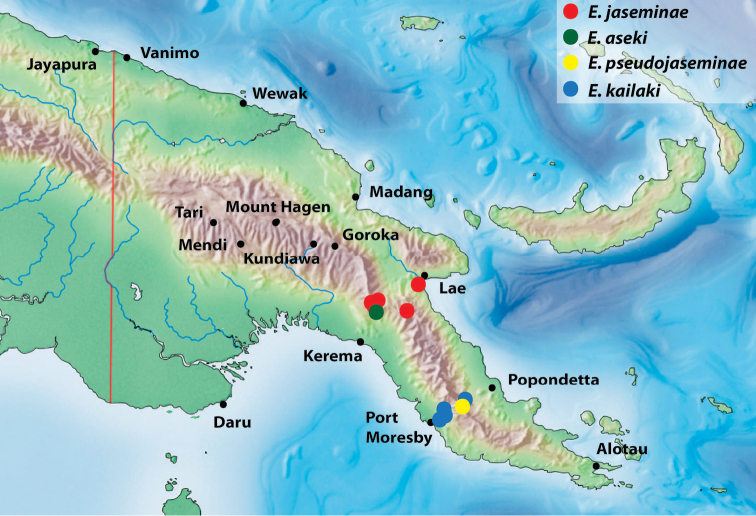
Map of the eastern part of New Guinea showing distribution of the species of the *E. jaseminae* group.

Papua New Guinea: Central Province (Fig. 34).

###### Etymology.

The species is named after Kailaki Village. The name is a noun in the nominative singular standing in apposition.

##### 13. Exocelina pseudojaseminae

Taxon classificationAnimaliaColeopteraDytiscidae

Shaverdo & Balke sp. nov.

E9FA166B-F0D2-5934-B1D3-6D25D0D8C619

http://zoobank.org/EE9EC355-5082-49BA-9A0B-563702855B0F

Figs 29 , 33

###### Type locality.

Papua New Guinea: Central Province, Kokoda Track, 09°14.34'S, 147°40.54'E, 1400 m a.s.l.

###### Type material.

*Holotype*: male “Papua New Guinea: Central, Kokoda Trek, 1400m, i.2008, [09°] 14.339S 147 40.538E, Posman (PNG 171)” (ZSM). *Paratypes*: 3 males, 2 females with the same label as the holotype (NHMW, ZSM).

###### Description.

*Body size and form*: Beetle medium-sized: TL-H 3.4–3.85 mm, TL 3.8–4.25 mm, MW 1.8–2.1 mm (holotype: TL-H 3.65 mm, TL 4.0 mm, MW 1.95 mm), with oblong-oval habitus.

*Colouration*: Brown to dark brown, with paler sides of pronotum and head. Head reddish brown, dark brown posterior to eyes. Pronotum reddish brown to brown, with reddish sides. Elytra brown to dark brown, sometimes with weak reddish sutural lines. Head appendages and legs proximally yellowish to reddish, legs distally darker, reddish brown (Fig. 29).

*Surface sculpture*: More or less shiny dorsally, with fine but distinct punctation and distinctly impressed microreticulation. Head with coarse and dense punctation (no spaces between punctures or spaces 1–2 times size of punctures); diameter of punctures equal to diameter of cells of microreticulation. Pronotum with much finer and sparser punctation than on head. Elytra with distinct punctation, slightly finer and sparser than on pronotum. Pronotum and elytra with weakly or more strongly impressed microreticulation; head with microreticulation stronger. Metaventrite, metacoxae, and abdominal ventrites distinctly microreticulate. Metacoxal plates with longitudinal strioles and weak transverse wrinkles. Abdominal ventrites with strioles and very fine, sparse punctation.

*Structures*: Pronotum with lateral bead. Base of prosternum and neck of prosternal process with distinct ridge, slightly rounded anteriorly. Blade of prosternal process lanceolate, relatively narrow, slightly convex, with distinct bead and few setae laterally. Abdominal ventrite 6 slightly truncate.

*Male*: Protarsomere 4 with large, thick, strongly curved anterolateral hook-like seta. Protarsomere 5 ventrally with anterior band of more than 60 and posterior band of ca. 30 relatively long setae, which connected approximately in middle (Fig. 33D). Abdominal ventrite 6 with 1–4 lateral strioles on each side. Median lobe with apical lobes very strongly developed, rounded in lateral view, “nose” small but distinct (Fig. 33A, B). Paramere as in Fig. 33C.

*Female*: Without evident differences in external morphology from males, except for not modified protarsi and abdominal ventrites 5 and 6 without strioles.

###### Affinities.

*Exocellina pseudojaseminae* sp. nov. can be distinguished by its size, dorsal colouration and punctation, shape and setation of its median lobe and paramere from the species of the *E. danae* group (*E. nomax* and *E. pulchella* sp. nov.) co-occurring in the same area. In its external appearance and shape of the median lobe, *E. pseudojaseminae* is very similar to *E. jaseminae* but it has more strongly developed apical lobes of the median lobe and much larger, hook-like anterolateral seta of the male protarsomere 4. For further affinities within the group, see the “Key”.

###### Distribution.

Papua New Guinea: Central Province (Fig. 34).

###### Etymology.

The species was mistaken for *E. jaseminae* due to their similarity in general appearance and shape of the median lobe. The name is a noun in the nominative singular standing in apposition.

##### Key to the species of *Exocelina jaseminae* group

**Table:** 

1	Beetle dorsally submatt, with strong and dense punctation and strongly impressed microreticulation (Fig. 26)	***aseki* sp. nov.**
–	Beetle dorsally shiny, with very weak punctation, often invisible on elytra, and weakly impressed microreticulation (e.g., Fig. 27)	**2**
2	Anterolateral seta of male protarsomere 4 thin, slightly curved, equal to or smaller than more laterally situated large setae (Fig. 31D). Median lobe and paramere as in Fig. 31A, C	***jaseminae* (Balke, 1998)**
–	Anterolateral seta of male protarsomere 4 hook-like, large, strongly curved	**3**
3	Apical lobes of median lobe weak, truncate in lateral view, “nose” indistinct (Fig. 32B). Paramere as in Fig. 32C. Beetle smaller, TL-H 3.1–3.85 mm (Fig. 28)	***kailaki* sp. nov.**
–	Apical lobes of median lobe strong, rounded in lateral view, “nose” distinct (Fig. 33B). Paramere as in Fig. 33C. Beetle larger, TL-H 3.4–3.85 mm (Fig. 29)	***pseudojaseminae* sp. nov.**

#### *Exocelina larsoni* group

This group is characterised by fine and sparse dorsal punctation; pronotum with very narrow lateral bead; median lobe of aedeagus with or without setation, very broad, robust, with sides strongly thickened; in ventral view, almost parallel-sided, with slight median constriction; apexes of ventral sclerites of median lobe very unequal: right one much longer than left one; paramere slightly concave on dorsal side and with long and dense subdistal and inconspicuous proximal setae.

##### 14. Exocelina larsoni

Taxon classificationAnimaliaColeopteraDytiscidae

(Balke, 1998)

92803D30-F939-5308-812E-99856A8F6091

Figs 35 , 40

Copelatus (Papuadytes) larsoni Balke, 1998: 332; Nilsson 2001: 77 (catalogue).
Papuadytes larsoni (Balke, 1998): Nilsson and Fery 2006: 56 (comb. nov.).
Exocelina larsoni (Balke, 1998): Nilsson 2007: 34 (comb. nov.).
Exocelina larsoni MB1299: Toussaint et al. 2014: supplementary figs 1–4, tab. 2; Toussaint et al. 2015: supplementary figs S1, S2, tab. S3, and information S5, S6.

###### Type locality.

Papua New Guinea: Madang Province, Baiteta, 05°01'00"S, 145°45'00"E, ca. 700 m a.s.l.

###### Type material studied.

*Paratypes*: 2 males “PAPUA NEW GUINEA Baiteta March 13, 1991 D. J. Larson” (NHMW). Note: According to the original description (Balke 1998), the holotype is deposited in the collection of D. Larson and is in the Australian National Insect Collection now. The holotype was not studied since the species is very characteristic and two paratypes from the same locality were examined.

###### Additional material.

**Madang**: 7 males, 2 females “Papua New Guinea: Madang Province, Wanang village, ca 110 m, 20.ix.2013, 05.15.458S 145.02.389E, David Boukal (PNG2013-13)” (NHMW, ZSM). 4 males, 5 females “Papua New Guinea: Madang Province, Wanang village env., Wanang conservation area, 250 m, 21.ix.2013, 05.15.458S 145.02.389E, David Boukal (PNG2013-20)” (NHMW, ZSM). 1 male “Papua New Guinea: Madang Province, Wanang village env., Wanang conservation area, 250 m, 22.ix.2013, 05.15.458S 145.02.389E, David Boukal (PNG2013-24)” (NHMW). 1 female “Papua New Guinea: Madang Province, Wanang village env., Wanang conservation area, 250 m, 22.ix.2013, 05.15.458S 145.02.389E, David Boukal (PNG2013-31)” (NHMW). 16 males, 4 females “Papua New Guinea: Madang Province, Wanang village env., Wanang conservation area, 250 m, 23.ix.2013, 05.15.458S 145.02.389E, David Boukal (PNG2013-33)” (NHMW, ZSM). 8 males, 11 females “Papua New Guinea: Madang Province, Wanang village env., Wanang conservation area, 250 m, 23.ix.2013, 05.15.458S 145.02.389E, David Boukal (PNG2013-37)” (NHMW, ZSM). 2 males, 1 female “Papua New Guinea: Madang Province, Wanang village env., Wanang conservation area, 250 m, 24.ix.2013, 05.15.458S 145.02.389E, David Boukal (PNG2013-39)” (NHMW). 33 males, 3 females “Papua New Guinea: Madang Province, Wanang village env., Wanang conservation area, 250 m, 25.ix.2013, 05.15.458S 145.02.389E, David Boukal (PNG2013-44)” (NHMW, ZSM). 4 males, 1 female “V.Kolář Lgt. Papua New Guinea Wanang III 4–20.7.2013” (NHMW). 1 female “Ibisca Niugini, PNG 18–20.xi.2012 Wanang -5,227670193 145,0797424”, “FIT-WAN-G-1/8-d01 / Plot 7 / P0596 Vial 22305-CODYTI” (ZSM). 1 male “Ibisca Niugini, PNG 20–22.xi.2012 Wanang -5,227670193 145,0797424”, “FIT-WAN-P-2/8-d04 / Plot 16 / P0669 Vial 22273-CODYTI” (ZSM). 1 female “Ibisca Niugini, PNG 24–26.xi.2012 Wanang -5,227670193 145,0797424”, “FIT-WAN-H-4/8-d07 / Plot 8 / P0607 Vial 17640-CODYTI” (ZSM). 1 male “Ibisca Niugini, PNG 30.x.–2.xii.2012 Wanang -5,227670193 145,0797424”, “FIT-WAN-L-7/8-d14 / Plot 12 / P0642 Vial 17744-CODYTI” (ZSM). 1 female “Ibisca Niugini, PNG 2–4.xii.2012 Wanang FIT-WAN-D-8/8-d15 / Plot 4 / P0579 Vial 17568-CODYTI” (ZSM). 3 males “Papua New Guinea: Madang, below Bundi, 500 m, 26.IX.2002 Balke & Sagata (PNG 023)”, one of them with an additional green label “269 DNA M Balke” and “sp 24 SEM 19” (ZSM). 5 males, 3 females “Papua New Guinea: Madang, Brahmin, 150 m, 26.IX.2002 Balke & Sagata (PNG 024)”, two of males with additional green labels “274 DNA M Balke”, “275 DNA M Balke” (NHMW, ZSM). 1 male “Papua New Guinea: Madang, Adalbert [sic!] Mts., Keki, 850m, 4.v.2006, nr 04.42.300S 145.25.089E, Manaono leg. (PNG 52)” (ZSM). 2 males, 5 females “Papua New Guinea: Madang, Adalbert [sic!] Mts., creek nr Keki, 790m, 28.xi.1994, 04.42.300S 145.25.089E, Binatang Boys leg. (PNG 53a)” (ZSM). 1 male, 1 female “Papua New Guinea: Madang, Adalbert Mts., Keki to Sewan, 650m, 7.v.1994, 04.41.802S 145.25.460E, Balke (PNG 54)”, male with an additional green label “DNA M.Balke 1299” (ZSM). 16 males, 8 females “Papua New Guinea: Madang, Usino, 260m, 15.iii.2007, 05.31.125S 145.25.316E, Kinibel (PNG 158)”, one male with an additional green label “DNA M. Balke 3307” (NHMW, ZSM). 1 male, 1 female “Papua New Guinea: Madang, Mt. Tapo, 180 m, ii.2008 5 24.11.00 S 145 36 17 16 E, BRC leg. (PNG 178)” (ZSM). 4 males, 8 females “Papua New Guinea: Madang, Wannang, 270m 31.x.2008, 05.15.458S 145.02.389E, Posman, (PNG187)” (ZSM). 17 males, 25 females “Papua New Guinea: Madang, Wannang, 230m 3.x.2008, 05.17.235S 145.06.160E, Posman (PNG188)”, three males additionally with green labels “DNA M.Balke 3763”, “DNA M.Balke 3767”, “DNA M.Balke 3768” (NHMW, ZSM). 2 males “Papua New Guinea: Madang, Akameku - Brahmin, Bismarck Range, 250–500m, 25.xi.2006, nr 05.47.026S 145.24.131E, Balke & Kinibel (PNG 115)”, one of them with an additional green abel “DNA M.Balke 1363” (ZSM). 13 males, 22 females “Papua New Guinea: Madang, Keki, Adalbert Mts., 500m, 29.xi.1994, 04.43.058S 145.24.437E, Balke & Kinibel (PNG 118)” (NHMW, ZSM). 38 males, 56 females “Papua New Guinea: Madang, Keki, Adalbert Mts., 400m, 29.xi.1994, 04.43.058S 145.24.437E, Binatang Boys, (PNG 119)” (NHMW, ZSM). 60 males, 97 females “Papua New Guinea: Madang, Keki-Sewan, Adalbert Mts., 700m, 30.xi.1994 nr 04.41.802S 145.25.460E Binatang Boys (PNG 120)” (NHMW, ZSM). 7 males, 8 females “Papua New Guinea: Madang, Keki-Sewan, Adalbert Mts., 300m 30.xi.1994, 04.40.558S 145.27.187E, Binatang Boys, (PNG 121)” (ZSM). 1 female “Ibisca Niugini, PNG 25–27.x.2012 Mount Wilhelm 700m -5,731960773 145,2521667”, “FIT-MW700-C-1/8-d01 / Plot 3 / P1114 Vial 16039-CODYTI” (ZSM). 1 male “Ibisca Niugini, PNG 26–28.x.2012 Mount Wilhelm 700m FIT-MW700-O-1/8-d02 / Plot 1 / P1210 Vial 16172-CODYTI” (ZSM). 4 females “Ibisca Niugini, PNG 26–28.x.2012 Mount Wilhelm 700m -5,731960773 145,2521667 MW0700 / P1210 Vial 16172” (ZSM). 1 male, 1 female “Ibisca Niugini, PNG 26–28.x.2012 Mount Wilhelm 700m”, “-5,731960773 145,2521667 FIT-MW700-S-1/8-d02 / Plot 19 / P1242 Vial 16118-CODYTI” (ZSM). 2 males, 3 females “Ibisca Niugini, PNG 26–28.x.2012 Mount Wilhelm 700m -5,731960773 145,2521667 MW700 / P1234 Vial 16270” (ZSM). 1 female “Ibisca Niugini, PNG 27–29.x.2012 Mount Wilhelm 700m”, “-5,731960773 145,2521667 FIT-MW700-A-2/8-d03 / Plot 1 / P1099 Vial 15960-CODYTI” (ZSM). 2 females “Ibisca Niugini, PNG 27–29.x.2012 Mount Wilhelm 700m”, “-5,731960773 145,2521667 FIT-MW700-C-2/8-d03 / Plot 3 / P1115 Vial 15923-CODYTI” (ZSM). 1 male, 2 females “Ibisca Niugini, PNG 27–29.x.2012 Mount Wilhelm 700m -5,731960773 145,2521667”, “FIT-MW700-A-2/8-d03 / Plot 1 / P1099 Vial 15958-CODYTI” (ZSM). 1 female “Ibisca Niugini, PNG 27–29.x.2012 Mount Wilhelm 700m -5,731960773 145,2521667”, “FIT-MW700-G-2/8-d03 / Plot 7 / P1147 Vial 16042-CODYTI” (ZSM). 1 female “Ibisca Niugini, PNG 27–29.x.2012 Mount Wilhelm 700m -5,731960773 145,2521667”, “FIT-MW700-H-2/8-d03 / Plot 8 / P1155 Vial 15976-CODYTI” (ZSM). 2 females “Ibisca Niugini, PNG 27–29.x.2012 Mount Wilhelm 700m -5,731960773 145,2521667”, “FIT-MW700-E-2/8-d03 / Plot 5 / P1131 Vial 15937-CODYTI” (ZSM). 2 females “Ibisca Niugini, PNG 28–30.x.2012 Mount Wilhelm 700m -5,731960773 145,2521667 MW0700 / P1243 Vial 16156” (ZSM). 2 males, 5 females “Ibisca Niugini, PNG 28–30.x.2012 Mount Wilhelm 700m -5,731960773 145,2521667 MW700 / P1235 Vial 16164” (ZSM). 3 males, 5 females “Ibisca Niugini, PNG 28–30.x.2012 Mount Wilhelm 700m FIT-MW700-O-2/8-d0 / Plot 15 / P1211 Vial 16189-CODYTI” (ZSM). 1 female “Ibisca Niugini, PNG 30.x.–1.xi.2012 Mount Wilhelm 700m -5,731960773 145,2521667 MW0700 / P1252 Vial 15993” (ZSM). 1 female “Ibisca Niugini, PNG 1–3.xi.2012 Mount Wilhelm 700m -5,731960773 145,2521667 MW0700 / P1213 Vial 16236” (ZSM). 1 female “Ibisca Niugini, PNG 1–3.xi.2012 Mount Wilhelm 700m FIT-MW700-M-4/8-d08 / Plot 13 / P1197 Vial 15762-CODYTI” (ZSM). 1 female “Ibisca Niugini, PNG 2–4.xi.2012 Mount Wilhelm 700m -5,731960773 145,2521667”, “FIT-MW700-A-5/8-d09 / Plot 1 / P1102 Vial 16027-CODYTI” (ZSM). 2 females “Ibisca Niugini, PNG 2–4.xi.2012 Mount Wilhelm 700m -5,731960773 145,2521667”, “FIT-MW700-E-5/8-d09 / Plot 5 / P1134 Vial P1134-CODYTI” (ZSM). 1 female “Ibisca Niugini, PNG 3–5.xi.2012 Mount Wilhelm 700m -5,731960773 145,2521667 MW0700 / P1222 Vial 16098” (ZSM). 1 female “Ibisca Niugini, PNG 3–5.xi.2012 Mount Wilhelm 700m -5,731960773 145,2521667 MW0700 / P1254 Vial 16105” (ZSM). 1 female “Ibisca Niugini, PNG 3–5.xi.2012 Mount Wilhelm 700m FIT-MW700-M-5/8-d10 / Plot 13 / P1198 Vial 16087-CODYTI” (ZSM). 1 female “Ibisca Niugini, PNG 3–5.xi.2012 Mount Wilhelm 700m FIT-MW700-O-5/8-d10 / Plot 15 / P1214 Vial 16088-CODYTI” (ZSM). 1 male, 2 females “Ibisca Niugini, PNG 3–5.xi.2012 Mount Wilhelm 700m”, “-5,731960773 145,2521667 FIT-MW700-R-5/8-d10 / Plot 18 / P1238 Vial 15969-CODYTI” (ZSM). 1 female “Ibisca Niugini, PNG 3–5.xi.2012 Mount Wilhelm 700m”, “-5,731960773 145,2521667 FIT-MW700-K-5/8-d10 / Plot 11 / P1182 Vial 16083-CODYTI” (ZSM). 4 females “Ibisca Niugini, PNG 4–6.xi.2012 Mount Wilhelm 700m -5,731960773 145,2521667”, “FIT-MW700-E-6/8-d11 / Plot 5 / P1135 Vial 07232-CODYTI” (ZSM). 2 males, 1 female “Ibisca Niugini, PNG 4–6.xi.2012 Mount Wilhelm 700m”, “-5,731960773 145,2521667 FIT-MW700-A-6/8-d11 / Plot 1 / P1103 Vial 07195-CODYTI” (ZSM). 4 females “Ibisca Niugini, PNG 4–6.xi.2012 Mount Wilhelm 700m”, “-5,731960773 145,2521667 FIT-MW700-C-6/8-d11 / Plot 3 / P1119 Vial 07081-CODYTI” (ZSM). 1 female “Ibisca Niugini, PNG 4–6.xi.2012 Mount Wilhelm 700m FIT-MW700-D-6/8-d11 / Plot 4 / P1127 Vial 07061” (ZSM). 1 male, 1 female “Ibisca Niugini, PNG 5–7.xi.2012 Mount Wilhelm 700m -5,731960773 145,2521667 MW0700 / P1239 Vial 16263” (ZSM). 1 male, 1 female “Ibisca Niugini, PNG 6–8.xi.2012 Mount Wilhelm 700m -5,731960773 145,2521667”, “FIT-MW700-E-7/8-d13 / Plot 5 / P1136 Vial15886-CODYTI” (ZSM). 2 females “Ibisca Niugini, PNG 9–11.xi.2012 Mount Wilhelm 700m -5,731960773 145,2521667 MW0700 / P1217 Vial 16106” (ZSM). 1 male, 3 females “Ibisca Niugini, PNG 3–5.xi.2012 Mount Wilhelm 200m -5,739897251 145,329742 MW0200 / P0856 Vial 09605” (ZSM). 1 female “Ibisca Niugini, PNG 3–5.xi.2012 Mount Wilhelm 200m -5,739897251 145,329742 MW0200 / P0865 Vial 09576” (ZSM). **Eastern Highlands**: 6 males, 7 females “Papua New Guinea: Eastern Highlands, Bena Bridge, 1400m, 8.xii.2007, 06.10.781S 145.26.034E, Balke & Sagata (PNG 164)” (NHMW, ZSM).

###### Diagnosis.

**Figures 35–39.**
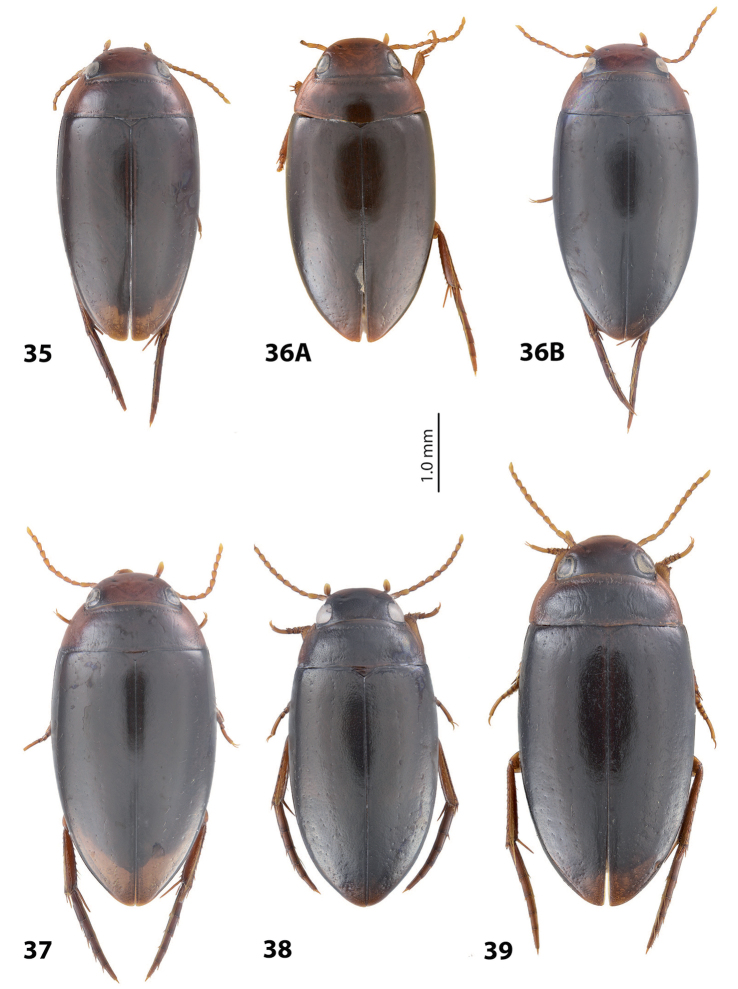
Habitus and colouration **35***Exocelina larsoni* (Balke, 1998) **36***E. nomax* (J. Balfour-Browne, 1939) **A** holotype **B** specimen from Tapini **37***E. warahulenensis* sp. nov. **38***E. mianminensis* sp. nov. **39***E. takime* (Balke, 1998).

**Figure 40.**
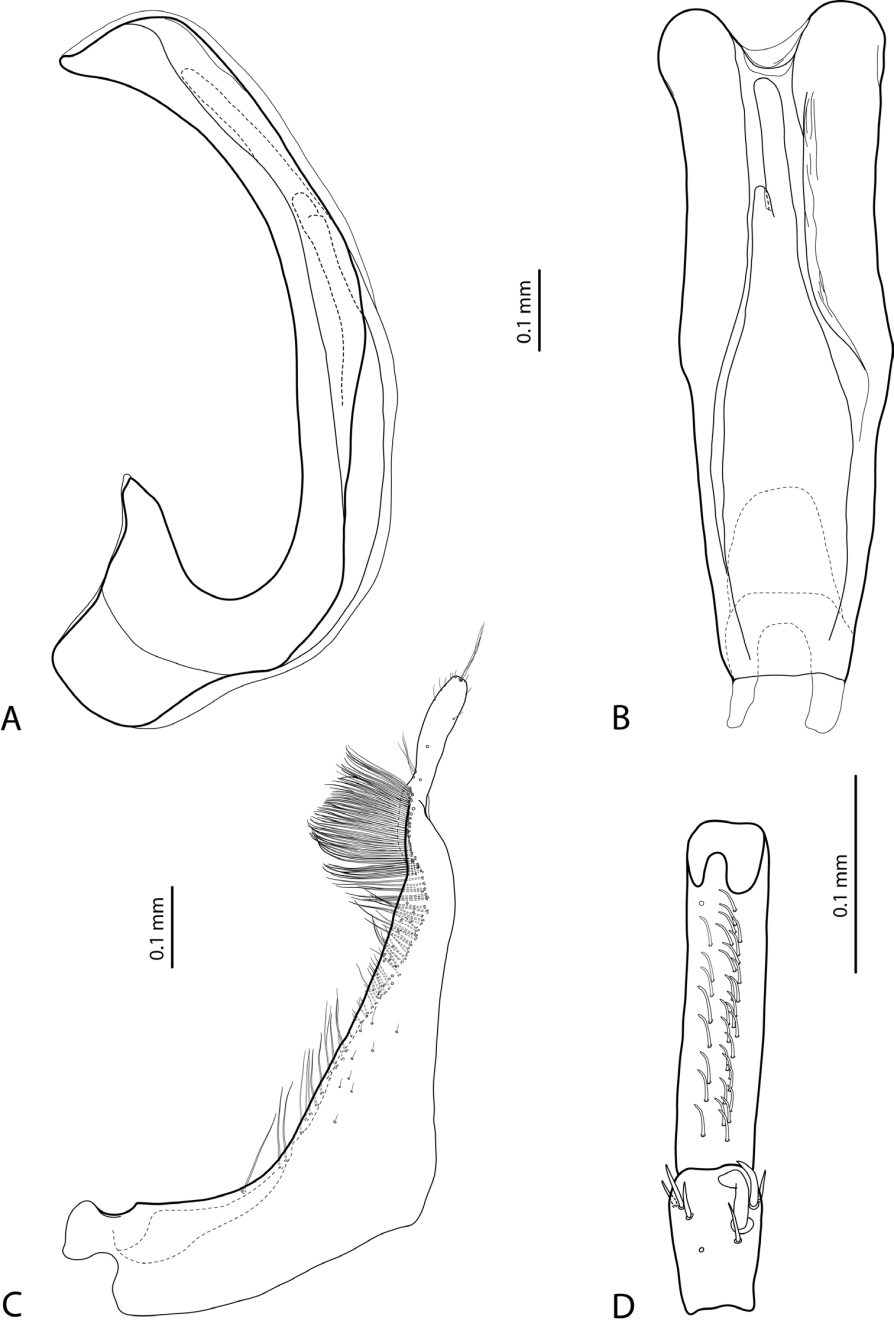
*Exocelina larsoni* (Balke, 1998) **A** median lobe in ventral view **B** median lobe in lateral view **C** paramere in external view **D** male protarsomeres 4–5 in ventral view.

For complete description, see Balke (1998: 332). Beetle small or medium-sized: TL-H 3.45–3.9 mm; oblong-oval, sometimes slightly more attenuated posteriorly; with characteristic dorsal colouration: dark (brown to piceous) elytra and pale (reddish to reddish brown pronotum, except for darker disc, and head; shiny, with very fine, on elytra often almost invisible punctation and weakly impressed microreticulation; pronotum with very narrow lateral bead (Fig. 35); male protarsomere 4 with medium-sized, thick, strongly curved anterolateral hook-like seta; male protarsomere 5 with anterior band of ca. 30 and posterior row of nine relatively long setae (Fig. 40D); median lobe robust, without lateral setae apically; in lateral view, with broadened, triangle apex; in ventral view, apex deeply concave; paramere very slightly concave on dorsal side and with long and dense subdistal and inconspicuous proximal setae (Fig. 40A–C).

###### Affinities.

In the area of its distribution, *E. larsoni* co-occurs with numerous species of the *E. ekari*, *E. ullrichi*, *E. broschii*, and *E. danae* groups. From all them, this characteristic species can be easily distinguished by its size, colouration, fine surface sculpture, simple male antennae, and mainly by the shape of the median lobe. Even females of the species differ from more similar in body form *E. brahminensis*Shaverdo et al., 2012 and *E. broschii* (Balke, 1998) in colouration (more uniform in two latter species) and narrow pronotal bead (absent *E. brahminensis* in and distinct in *E. broschii*). For affinities within the group, see the “Key”.

###### Distribution.

Papua New Guinea: Madang and Eastern Highlands Provinces. The species is known from numerous specimens from the central and wertern part of Madang and from northern part of Eastern Highlands (Fig. 45).

##### 15. Exocelina nomax

Taxon classificationAnimaliaColeopteraDytiscidae

(J. Balfour-Browne, 1939)

AC520018-98CC-5FFB-A6B8-FEAC44FACAC6

Figs 36 , 41

Copelatus nomax J. Balfour-Browne, 1939: 65–66; Guignot 1956: 55 (catalogue); Guéorguiev 1968: 34 (catalogue).
Copelatus nomax J. Balfour-Browne, 1939 sensu Guéorguiev 1978: 268–269 (key); Guéorguiev and Rocchi 1993: 148.
Copelatus (Papuadytes) nomax J. Balfour-Browne, 1939: Balke 1998: 334 (notes, diagnosis); Nilsson 2001: 77 (catalogue).
Papuadytes nomax (J. Balfour-Browne, 1939): Nilsson and Fery 2006: 56 (comb. nov.).
Exocelina nomax (J. Balfour-Browne, 1939): Nilsson 2007: 34 (comb. nov.).
Exocelina undescribed sp. MB3405: Toussaint et al. 2014: supplementary figs 1–4, tab. 2; Toussaint et al. 2015: supplementary figs S1, S2, tab. S3, and information S5, S6.

###### Type locality.

Papua New Guinea: Central Province, Mafulu, ca. 08°30'S, 147°00'E, ca. 1219 m a.s.l.

###### Type material.

*Holotype*: female “Type” [round, with red bead], “PAPUA: Mafulu. 4,000ft. i.1934. L.E.Cheesman. B.M.1934-321.”, “Copelatus nomax, ♀ Type nov.sp.” [hw, the word “type” with red ink], “Holotype” [red] (BMNH).

###### Additional material.

**Central**: 23 males, 32 females “Papua New Guinea: Central, Kokoda Trek, 320m, i.2008 [09°] 19.236S 147.31.791E, Posman (PNG 168)”, one male with an additional green label “DNA M.Balke 3405” (NHMW, ZSM). 4 males “Papua New Guinea: Central, Kokoda Trek, 980m, i.2008, [09°] 15.933S 147.36.590E, Posman (PNG 169)”, one of them with an additional green label “DNA M.Balke 3411” (ZSM). 1 female “Papua New Guinea: Central, Kokoda Trek, 980m, i.2008, [09°] 15.933S 147.36.590E, Posman (PNG 169)”, “DNA M.Balke 4117” [green] (ZSM). 10 males, 2 females “Papua New Guinea: Central, Kokoda Trek, 590m, i.2008, [09°] 14.339S 147.36.920E, Posman (PNG 170)” (NHMW, ZSM). 19 males, 31 females “Papua New Guinea: Central, Tapini, 1200m, 31.x.2007, 08.21.557S 146.58.712E, Kinibel (PNG 162)”, one of the males with an additional green label “DNA M.Balke 3306” (NHMW, ZSM). 2 males, 3 females “Papua New Guinea: Central, Moreguina [10°'57"S, 148°28'27"E], 16.viii.2008, Posman (PNG 183)”, males and one female with additional green labels “DNA M.Balke 3745”, “DNA M.Balke 3816”, “DNA M.Balke 3746” respectively (ZSM). 5 males, 9 females “Papua New Guinea: Central, Moroka area, Kailaki, 827 m, 26.x.2009, 9.24.134S 147.33.521E, Sagata (PNG225)” (NHMW, ZSM). 14 males, 8 females “Papua New Guinea: Central, Moroka, Kailaki, 827 m, 26.x.2009, 9.24.113S 147.33.524E, Sagata (PNG226)” (NHMW, ZSM). 1 female “Papua New Guinea Central, Moroka, Kailaki Wareaga, 760m, 27x2009 9.25.424S 147.31.068E Sagata (PNG227)” (ZSM). 27 males, 39 females “Papua New Guinea: Central, 755m, 28.x.2009 S9 25 47 5 E147 32 59.1, Sagata (PNG229)” (NHMW, ZSM). **National Capital District**: 2 males, 3 females “Papua New Guinea: National Capital District, Varirata NP, 600m, 16.xii.2007, 09.26.13S 147.22.09E, Balke & Sagata (PNG 159)”, males with additional green labels “DNA M.Balke 3304” and “DNA M.Balke 3305” (ZSM).

###### Redescription.

*Body size and form*: Beetle small: TL-H 3.0–3.8 mm, TL 3.45–4.2 mm, MW 1.7–2.15 mm (holotype: TL-H 3.5 mm, TL 3.95 mm, MW 1.95 mm), with oblong-oval habitus.

*Colouration*: Dark brown, with paler sides of pronotum and head. Head reddish to reddish brown, darker posterior to eyes. Pronotum brown to dark brown on disc and reddish to reddish brown on sides. Elytra uniformly dark brown. Head appendages and legs proximally yellowish, legs distally darker, reddish brown (Fig. 36). Teneral specimen paler.

*Surface sculpture*: Shiny dorsally, with inconspicuous punctation and weakly impressed microreticulation. Head with fine and sparse punctation (spaces between punctures 1–3 times size of punctures); diameter of punctures smaller than or equal to diameter of cells of microreticulation. Pronotum with finer and sparser punctation than on head. Punctation on elytra finer and sparser than on pronotum, inconspicuous, in some specimens invisible. Disc of pronotum and elytra with weakly impressed microreticulation; head and lateral sides of pronotum with microreticulation stronger. Metaventrite, metacoxae, and abdominal ventrites distinctly microreticulate. Metacoxal plates with longitudinal strioles and weak transverse wrinkles; abdominal ventrites with strioles. Punctation on venter inconspicuous, slightly stronger on two last abdominal ventrites.

*Structures*: Pronotum with narrow lateral bead. Base of prosternum and neck of prosternal process with distinct ridge, rounded anteriorly. Blade of prosternal process lanceolate, narrow, slightly convex, with distinct bead and few setae laterally. Abdominal ventrite 6 truncate.

**Figures 41, 42.**
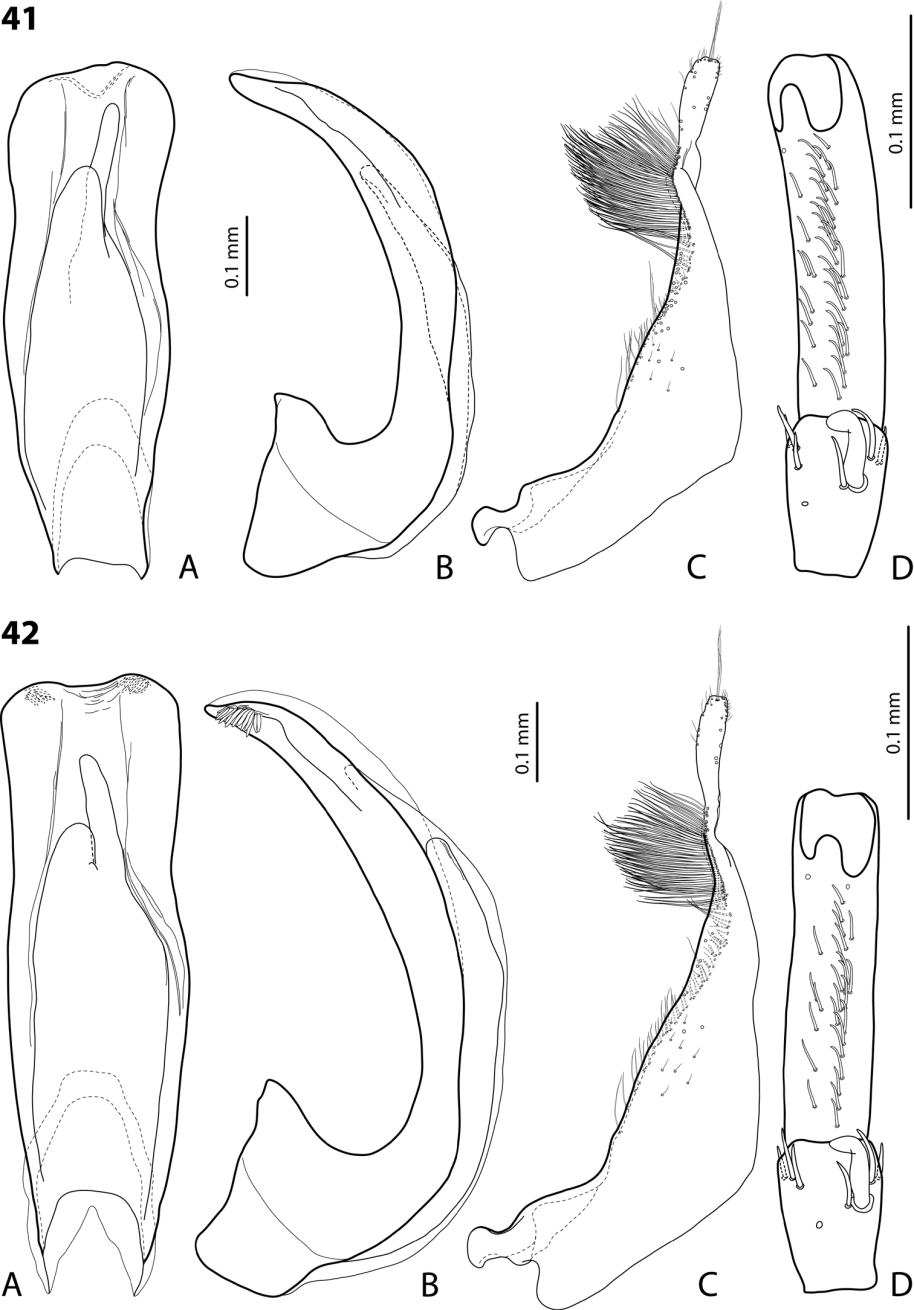
**41***Exocelina nomax* (J. Balfour-Browne, 1939) **42***E. warahulenensis* sp. nov. **A** median lobe in ventral view **B** median lobe in lateral view **C** paramere in external view **D** male protarsomeres 4–5 in ventral view.

*Male*: Protarsomere 4 with large, thick, strongly curved anterolateral hook-like seta. Protarsomere 5 ventrally with anterior band of ca. 30 and posterior row of nine relatively long setae (Fig. 41D). Abdominal ventrite 6 with 7–11 lateral striae on each side. Median lobe slender, without lateral setae apically; in lateral view, evenly curved to broadly pointed, elongate apex; in ventral view, apex slightly truncate, asymmetrical (Fig. 41A, B). Paramere very slightly concave on dorsal side, with long and dense subdistal and inconspicuous proximal setae (Fig. 41C).

*Female*: Without evident differences in external morphology from males, except for not modified protarsi and abdominal ventrite 6 without striae.

###### Notes on identity of the additional material with the holotype, affinities.

Balke (1998: 334) indicates *Exocelina nomax* as species minus cognitus. However, our study of the material collected from Tapini, village ca. 20 km north to Mafulu, allows us to consider with certain confidence that it belongs to *E. nomax*. It is the only *Exocelina* species collected from this area, and it was collected in abundant number (50 specimens) in Tapini. We assume this locality as the most northern distribution border of the species, which is very numerous in Kokoda and Kailaki areas. Morphologically, the specimens from Tapini, Kokoda, Kailaki and Varirata are identical to the holotype (Fig. 36A, B). Only three species occur close to Tapini-Mafulu area: *E. garaina*, *E. posmani* Shaverdo & Balke, 2016, and *E. woitapensis* Shaverdo & Balke, 2016 (the *E. danae* group). But they are larger (TL-H 3.6–4.5 mm), and the smallest of them, *E. woitapensis*, is matt dorsally. Moreover, *E. nomax* can be differentiated from them by its narrow pronotal bead, a very characteristic feature. Smaller size and narrow bead of the pronotum can be used to distinguish it from E. *jaseminae*, a species very similar to it in colour and surface structures. From the other species co-occurring in the Central and National Capital District Provinces (*E. bacchusi*, *E. pulchella* sp. nov., and species of the *E. danae* and *E. jaseminae* groups), *E. nomax* can be distinguished by its body size and colouration, dorsal punctation and microreticulation, narrow pronotal bead, and shape and setation of its median lobe and paramere. See also under *E. warahulenensis* sp. nov. and “Key”.

The specimen from Telefomin (Sandaun Province), 1 male “PAPUA, Selminumtem [Selminum Tem, 45 km SWS Telefomin, ca. 5°S; 141°15'E], W.Sepik d. P.Beron leg.”, “Copelatus nomax J.B.Br. det.V. Guéorguiev 1917” [partly hw] (NHMW), which was identified by Guéorguiev & Rocchi (1993: 148) as *E. nomax* and indicated by Balke (1998: 334, 338) as “sp. 4” has been recently described under the name *E. okbapensis* Shaverdo & Balke, 2017 (Shaverdo et al. 2017).

###### Distribution.

Papua New Guinea: Central and National Capital District Provinces (Fig. 45). The species is known from numerous specimens in the Central Province and a small population in the National Capital District.

##### 16. Exocelina warahulenensis

Taxon classificationAnimaliaColeopteraDytiscidae

Shaverdo & Balke sp. nov.

1F73681E-510A-532A-9888-0E378BB11F16

http://zoobank.org/C9CEE1CF-A8D3-4D90-967A-65BD803519F7

Figs 37 , 42

Exocelina undescribed sp. MB0265: Toussaint et al. 2014: supplementary figs 1–4, tab. 2; Toussaint et al. 2015: supplementary figs S1, S2, tab. S3, and information S5, S6.

###### Type locality.

Papua New Guinea: Simbu Province, Crater Mountain, Haia, ca. 06°39'39.9"S, 145°00'28.4"E, 700 m a.s.l.

###### Type material.

*Holotype*: male “Papua New Guinea: Crater Mountain, Haia, 700m, 11IX2002, Balke & Sagata, (PNG 001)” (ZSM). *Paratypes*: **Simbu**: 12 males, 7 females with the same label as the holotype (NHMW, ZSM). 2 males “PAPUA NEW GUINEA: Simbu / EHPr. Crater Mountain, Haia, 700m, 11IX2002, Balke & Sagata, (PNG 1)”, “258 DNA M Balke” [green], “259 DNA M Balke” [green] (ZSM). 2 males, 4 females “Papua New Guinea: Crater Mountain, trek Haia - Wara Sera, 500m, 12IX2002, Balke & Sagata, (PNG 004)” (ZSM). 5 males, 3 females “Papua New Guinea: Crater Mountain, trek Haia - Wara Sera, 500m, 12IX2002, Balke & Sagata, (PNG 005)” (NHMW, ZSM). **Simbu/Eastern Highlands**: 1 male “265 DNA M Balke” [green], “PNG Simbu / EHPr. Crater Mountain, Sera - Herowana, Wara Hulene, 1000 m, 16IX2002, Balke & Sagata (PNG 17)” (ZSM).

###### Description.

*Body size and form*: Beetle small to medium-sized: TL-H 3.4–3.9 mm, TL 3.7–4.3 mm, MW 1.8–2.2 mm (holotype: TL-H 3.9 mm, TL 4.3 mm, MW 2.1 mm), with oblong-oval habitus.

*Colouration*: Piceous, with reddish sides of pronotum and head. Head reddish to reddish brown, darker posterior to eyes. Pronotum brown to piceous on disc and reddish to reddish brown on sides. Elytra dark brown to piceous. Head appendages and legs proximally yellowish, legs distally darker, reddish brown (Fig. 37). Teneral specimen paler.

*Surface sculpture*: Shiny dorsally, with inconspicuous punctation and weakly impressed microreticulation. As in *E. nomax*.

*Structures*: Pronotum with narrow lateral bead. Base of prosternum and neck of prosternal process with distinct ridge, rounded anteriorly. Blade of prosternal process lanceolate, relatively broad, slightly convex, with distinct bead and few setae laterally. Abdominal ventrite 6 truncate or slightly concave.

*Male*: Protarsomere 4 with large, thick, strongly curved anterolateral hook-like seta. Protarsomere 5 ventrally with anterior row of 20 and posterior row of seven relatively long setae (Fig. 42D). Abdominal ventrite 6 with 9–12 lateral striae on each side. Median lobe slender, with lateral setae apically; in lateral view, evenly curved to broadly pointed, short apex; in ventral view, apex slightly concave (Fig. 42A, B). Paramere very slightly concave on dorsal side, with long and dense subdistal and inconspicuous proximal setae (Fig. 42C).

*Female*: Without evident differences in external morphology from males, except for not modified protarsi and abdominal ventrite 6 without striae.

###### Affinities.

*Exocellina warahulenensis* sp. nov. can be distinguished by body size, form, colouration, inconspicuous dorsal punctation, simple male antenna, and shape and setation of its median lobe and paramere from the species co-occurring in the same area (*E. damantiensis*, *E. hintelmannae*, and *E. ullrichi* (Balke, 1998)). In the dorsal colouration and surface sculpture, the new species is similar to *E. larsoni* but differs from it in shape and presence of setation of the median lobe. *Exocelina warahulenensis* sp. nov. is also very similar to *E. nomax* but is slightly larger and has darker colouration and longer median lobe, with lateral setae apically and shorter, broader apex in lateral view.

###### Distribution.

Papua New Guinea: Simbu and Eastern Highlands Provinces (Fig. 45). The species is known only from Crater Mountain area.

###### Etymology.

The species is named after Wara Hulene Village where one of the paratype was collected. The name is an adjective in the nominative singular.

##### Key to the species of *Exocelina larsoni* group

**Table:** 

1	Median lobe robust, lateral sides strongly thickened and apex much broader in lateral view and deeply concave in ventral view (Fig. 40A). Paramere as in Fig. 40C	***larsoni* (Balke, 1998)**
–	Median lobe slender, lateral sides more weakly thickened and apex thinner in lateral view and slightly concave in ventral view (e.g., Fig. 41). Paramere as in e.g., Fig. 41C	**2**
2	Beetle brownish, usually smaller, TL-H 3.0–3.8 mm (Fig. 36). Apex of median lobe elongate and thinner in lateral view, without lateral setae (Fig. 41A). Paramere as in Fig. 41C	***nomax* (J. Balfour-Browne, 1939)**
–	Beetle piceous, usually larger, TL-H 3.4–3.9 mm (Fig. 37). Apex of median lobe shorter and broader in lateral view, with lateral setae (Fig. 42A). Paramere as in Fig. 42C	***warahulenensis* sp. nov.**

#### *Exocelina takime* group

This group is characterised by more or less coarse and dense dorsal punctation; pronotum with narrow lateral bead; median lobe of aedeagus without setation, broad, robust, sides slightly thickened; in ventral view, it broadened medially or subdistally; apexes of ventral sclerites of median lobe almost equal; paramere with distinct dorsal notch and subdistal part well developed, with long and dense subdistal and inconspicuous proximal setae.

##### 17. Exocelina mianminensis

Taxon classificationAnimaliaColeopteraDytiscidae

Shaverdo & Balke sp. nov.

D5CB43EB-7D46-5FAB-8B2D-E29C5ED73336

http://zoobank.org/10F38B28-E1B6-46F2-B443-7C19A4A36D18

Figs 38 , 43

Exocelina undescribed sp. MB0688: Toussaint et al. 2014: supplementary figs 1–4, tab. 2; Toussaint et al. 2015: supplementary figs S1, S2, tab. S3, and information S5, S6.

###### Type locality.

Papua New Guinea: Sandaun Province, Mianmin, 04°52.86'S, 141°31.71'E, 700 m a.s.l.

###### Type material.

*Holotype*: male “Papua New Guinea: Sandaun, Mianmin (pool), 700m, 21.x.2008, 04.52.858S 141.31.706E, Ibalim (PNG 198), “DNA M.Balke 3749” [green] (ZSM). *Paratypes*: 16 males, 8 females with the same label as the holotype, one male with an additional green label “DNA M.Balke 3758” (NHMW, ZSM). 2 males, 3 females “Papua New Guinea: Sandaun, Mianmin (pool), 700m, 21.x.2008, 04.52.858S 141.31.706E, Ibalim (PNG 197) (ZSM). 1 male “Papua New Guinea: Sandaun, Mianmin (pool), 990m, 23.x.2008, 4.54.570S 141.35.490E, Ibalim (PNG 193) (ZSM). 4 males “Papua New Guinea: Sandaun, May River, 970m, 19.x.2003, 4.49.779S 141.38.174E, K. Sagata (WB43)”, one of them with an additional green label “DNA MB688” (ZSM).

###### Description.

*Body size and form*: Beetle medium-sized: TL-H 3.75–4.25 mm, TL 4.15–4.6 mm, MW 1.95–2.3 mm (holotype: TL-H 3.75 mm, TL 4.15 mm, MW 1.95 mm), with oblong habitus.

*Colouration*: Piceuos. Head piceous, with reddish brown anterior margin. Pronotum dark brown to piceous, with reddish brown to brown sides. Elytra uniformly piceous. Head appendages and legs proximally yellowish, legs distally darker, reddish brown (Fig. 38). Teneral specimen paler.

*Surface sculpture*: Submatt dorsally, with dense and coarse punctation and weakly impressed microreticulation. Head with very dense and coarse punctation (no spaces between punctures or spaces 1–2 times size of punctures); diameter of punctures equal to diameter of cells of microreticulation. Pronotum with distinct punctation, finer than on head. Punctation on elytra distinct, finer and sparser than on head. Elytra with weakly impressed microreticulation; head and pronotum with microreticulation stronger than on elytra. Metaventrite, metacoxae, and abdominal ventrites distinctly microreticulate. Metacoxal plates with longitudinal strioles and weak transverse wrinkles; abdominal ventrites 2–4 with few strioles, two last one without strioles but with very weak wrinkles. Punctation on venter fine but distinct.

*Structures*: Pronotum with narrow lateral bead. Base of prosternum and neck of prosternal process with distinct ridge, rounded anteriorly. Blade of prosternal process lanceolate, narrow, slightly convex, with distinct bead and few setae laterally. Abdominal ventrite 6 truncate or very slightly concave.

**Figures 43, 44.**
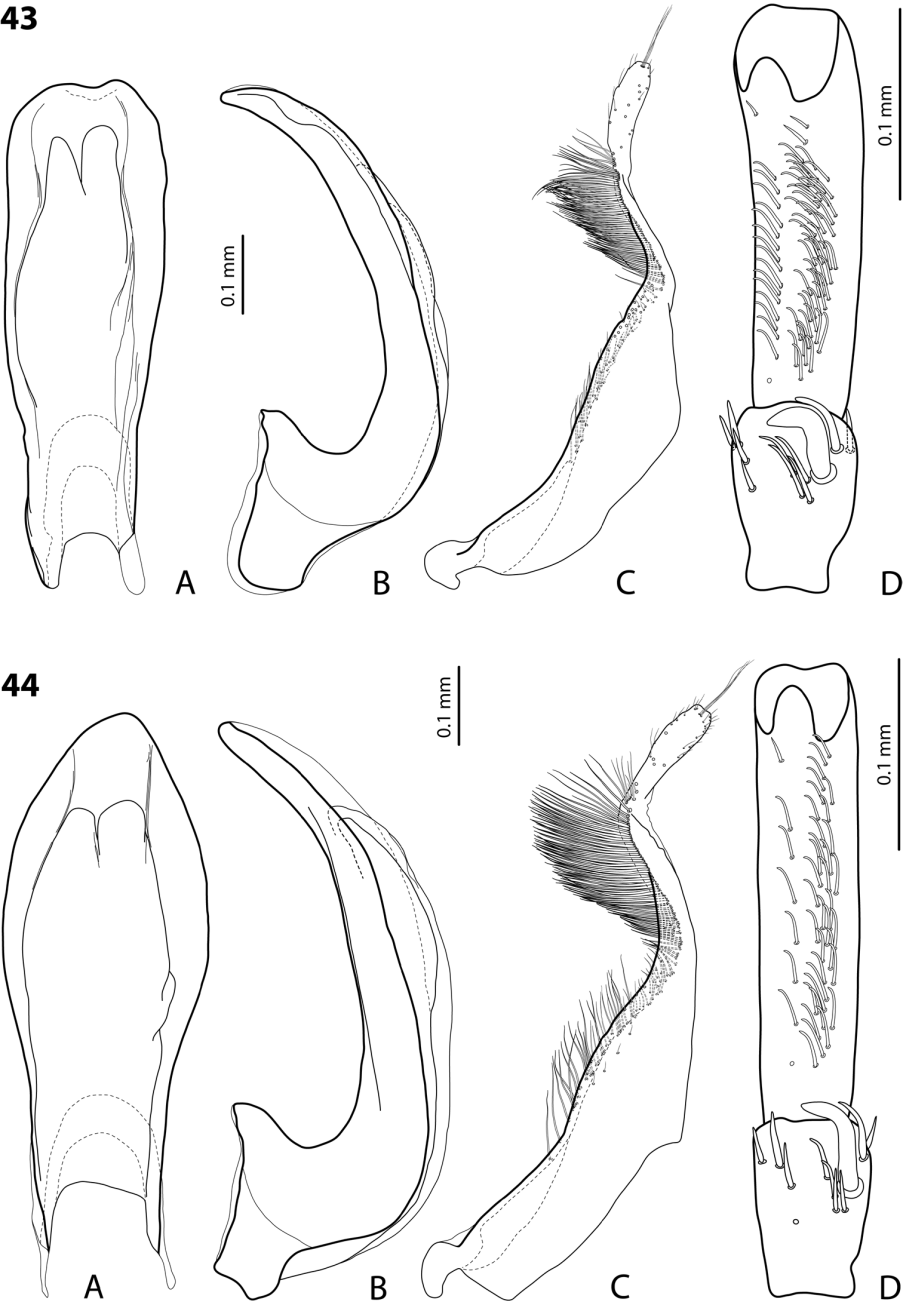
**43***Exocelina mianminensis* sp. nov. **44***E. takime* (Balke, 1998) **A** median lobe in ventral view **B** median lobe in lateral view **C** paramere in external view **D** male protarsomeres 4–5 in ventral view.

*Male*: Protarsomere 4 with large, thick, strongly curved anterolateral hook-like seta. Protarsomere 5 ventrally with anterior band of ca. 60 and posterior row of 17 relatively long setae (Fig. 43D). Abdominal ventrite 6 without lateral striae on each side, except one with setae. Median lobe slender, lateral sides slightly thickened; in lateral view, apex short, pointed, and curved downwards; in ventral view, lateral sides evenly expanded subdistally and apex slightly concave (Fig. 43A, B). Paramere with distinct dorsal notch and subdistal part well developed, with long and dense subdistal and inconspicuous proximal setae (Fig. 43C).

*Female*: Without evident differences in external morphology from males, except for not modified protarsi.

###### Affinities.

In the area of its distribution, *E. mianminensis* co-occurs with species of the *E. ekari*, *E. okbapensis*, *E. broschii*, *E. casuarina* and *E. danae* groups. From species of the *E. ekari* group, the species differs in larger size, presence of the pronotal bead, evidently stronger dorsal punctation, and the shape of the median lobe. From the other species, *E. mianminensis* sp. nov. can be distinguished by body size, form, and colouration, dorsal punctation, simple male antenna, and shape and setation of its median lobe and paramere. In the general appearance, the new species is more similar to *E. ibalimi*Shaverdo et al., 2018, but can be easily distinguished from it in presence of the pronotal bead. Male abdominal ventrite 6 without lateral striae was so far known only for *E. sima*Shaverdo et al., 2018 among New Guinea *Exocelina*. For affinities within the group, see the “Key”.

###### Distribution.

**Figure 45.**
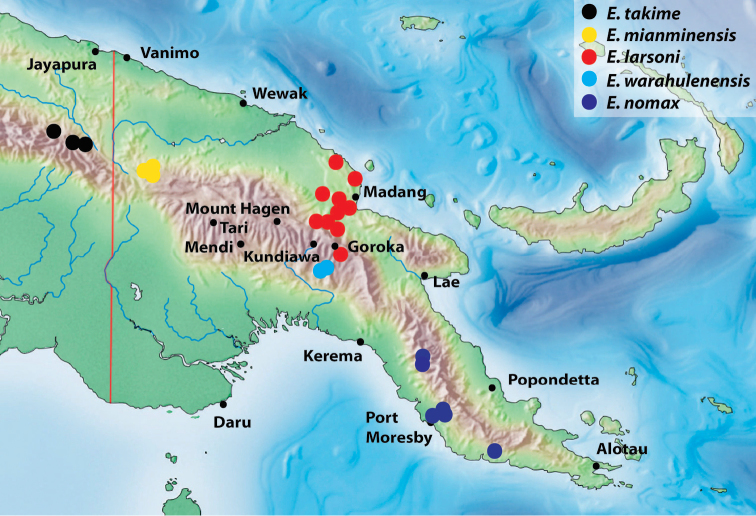
Map of the eastern part of New Guinea showing distribution of the species of the *E. larsoni* and *takime* groups.

Papua New Guinea: Sandaun Province (Fig. 45).

###### Etymology.

The species is named after Mianmin Village. The name is an adjective in the nominative singular.

##### 18. Exocelina takime

Taxon classificationAnimaliaColeopteraDytiscidae

(Balke, 1998)

733BAC0C-78D7-5CBF-BC8D-78BA8FBAC482

Figs 39 , 44

Copelatus (Papuadytes) takime Balke, 1998: 336; Nilsson 2001: 77 (catalogue).
Papuadytes takime (Balke, 1998): Nilsson and Fery 2006: 56 (comb. nov.).
Exocelina takime (Balke, 1998): Nilsson 2007: 34 (comb. nov.).

###### Type locality.

Indonesia: Papua Province: Pegunungan Bintang Regency, Bime, ca. 04°20'S, 140°12'E, 1400 m a.s.l.

###### Type material studied.

*Holotype*: male “IRIAN JAYA: 11.9.1993 Bime – Calab Gebiet, Bime, 1400m”, “leg. M. Balke (12) ca. 140°12'E 04°20'S”, “HOLOTYPUS” [red], “Copelatustakime Balke des. 1997” [red] (NHMW). *Paratypes*: 10 males, 14 females with the same label as the holotype, one female with an additional green label “DNA M.Balke 3291” (NHMW). 11 males, 2 females “IRIAN JAYA: 22.9.1993 Bime – Calab Gebiet, Bime, 1400m”, “ca. 140°12'E 04°20'S, leg. Balke (16)”, one of the males with an additional green label “DNA M.Balke 3290” (NHMW). 3 males, 1 female “IRIAN JAYA Zentralmassiv 140°25'E 04°24'S”, “Kali Takime, 900m 18.8.1992 leg. Balke (17)” (NHMW). 7 males, 6 females “IRIAN JAYA: 29.9.1993 Eme Gebiet Emdoman, 800m”, “ca. 139°55'E 04°14'S, leg. M. Balke (24)” (NHMW). All these specimens are with red paratype labels “PARATYPUS Copelatustakime Balke des. 1997” or “Paratypus Copelatustakime Balke des. 1997” [red]. Note: in the original description (Balke 1998), 8 males and 4 females are reported from the locality 16; there are 11 males, 2 females in the NHMW, therefore, some mistakes were made in presenting the type material in the original description or/and during labeling the material.

###### Additional material.

2 females “IRIAN JAYA: 11.9.1993 Bime – Calab Gebiet, Bime, 1400m”, “leg. M. Balke (12) ca. 140°12'E 04°20'S” (NHMW).

###### Diagnosis.

For complete description, see Balke (1998: 336). Beetle medium-sized: TL-H 4.1–4.5 mm; oblong-oval; dark brown to piceous, with brownish pronotal sides and head anteriorly; shiny, but with distinct dorsal punctation and weakly impressed microreticulation; pronotum with narrow lateral bead (Fig. 39); male protarsomere 4 with large, thick, strongly curved anterolateral hook-like seta; male protarsomere 5 with anterior band of ca. 40 and posterior row of eight relatively long setae (Fig. 44D); median lobe robust, in lateral view, evenly curved to narrowly rounded, not curved downwards apex; in ventral view, with more strongly thickened lateral sides, distinctly expanded in middle and narrowing to broadly pointed apex; paramere with distinct dorsal notch and subdistal part well developed, with long and dense subdistal and inconspicuous proximal setae (Fig. 44A–C).

###### Affinities.

In the area of its distribution, *E. takime* co-occurs with *E. aipomek*, *E. ascendens* and species of the *E. bacchusi*, *E. ekari*, *E. aipo*, *E. okbapensis*, *E. casuarina*, and *E. danae* groups. From species of the *E. ekari* group, the species differs in larger size, evidently stronger dorsal punctation, and the shape of the median lobe. In the latter character, *E. takime* differs also from the species of the remaining groups. For separating it from some of these species, also presence of the pronotal bead and simple male antennae, and shape and setation of the paramere can be used. For affinities within the group, see the “Key”.

###### Distribution.

Indonesia: Papua Province: Pegunungan Bintang Regency. The species is known only from the type material, i.e., Borme – Bime – Emdoman area (Fig. 45).

##### Key to the species of *Exocelina takime* group

**Table:** 

1	Beetle smaller, TL-H 3.75–4.25 mm. Dorsal punctation coarser (Fig. 38). Median lobe slender, with lateral sides slightly thickened; in ventral view, evenly expanded subdistally, apex slightly concave; in lateral view, apex short, pointed, and curved downwards (Fig. 43). Paramere as in Fig. 43C	***mianminensis* sp. nov.**
–	Beetle larger, TL-H 4.1–4.5 mm. Dorsal punctation finer (Fig. 39). Median lobe with strongly thickened lateral sides; in ventral view, distinctly expanded in middle and narrowing to broadly pointed apex; in lateral view, evenly curved to narrowly rounded, not curved downwards apex (Fig. 44). Paramere as in Fig. 44C	***takime* (Balke, 1998)**

#### *Exocelina warasera* group

This group is characterised by extremely fine and sparse dorsal punctation; pronotum with distinct lateral bead; median lobe of aedeagus simple; in lateral view, slightly or more strongly curved, apex slightly curved downwards and bluntly pointed; in ventral view, apex bluntly pointed and often twisted sidewards; apexes of ventral sclerites of median lobe almost equal; paramere slightly concave on dorsal side, subdistal setae strong and dense, proximal setae usually inconspicuous.

##### 19. Exocelina haia

Taxon classificationAnimaliaColeopteraDytiscidae

Shaverdo & Balke sp. nov.

4DF0A0EF-01DF-5152-AF14-6ABE6886DD34

http://zoobank.org/D1562E8F-F21A-4588-9F23-23E6142A5272

Figs 49 , 53

###### Type locality.

Papua New Guinea: Simbu Province, between Supa and Haia Villages (Airstrips), ca. 6°39'39.9"S, 145°00'28.4"E, 1032 m.

###### Type material.

*Holotype*: male “Papua New Guinea: Supa Haia, 1023m, 10.ix.2002, K. Sagata (WB1)” (ZSM). *Paratypes*: 2 males with the same label as the holotype (NHMW, ZSM). 1 male “Papua New Guinea: Crater Mountain, trek Haia - Wara Sera, 500m, 12IX2002, Balke & Sagata, (PNG 005)” (ZSM).

###### Females of doubtful identity.

See under *E. bacchusi*.

###### Description.

*Body size and form*: Beetle small: TL-H 3.4–3.45 mm, TL 3.7–3.75 mm, MW 1.8 mm (holotype: TL-H 3.4 mm, TL 3.7 mm, MW 1.8 mm), with oblong-oval habitus.

*Colouration*: Dark brown, with paler sides of pronotum and head anteriorly. As in *E. warasera* sp. nov. (Fig. 49).

*Surface sculpture*: Shiny dorsally, with extremely fine and sparse punctation and weakly impressed microreticulation. As in *E. warasera* sp. nov.

*Structures*: Pronotum with lateral bead. Base of prosternum and neck of prosternal process with distinct ridge, slightly rounded anteriorly. Blade of prosternal process lanceolate, relatively narrow, slightly convex, with distinct bead and few setae laterally. Abdominal ventrite 6 very slightly concave.

*Male*: Protarsomere 4 with anterolateral seta thin and evenly curved, smaller than more laterally situated large seta. Protarsomere 5 ventrally with anterior band of ca. 80 and posterior row of two relatively long setae (Fig. 53D). Abdominal ventrite 6 with 6–8 lateral striae on each side. Median lobe short, curved, with broadly pointed apex in lateral view, and evenly tapering to pointed apex in ventral view (Fig. 53A, B). Paramere concave on dorsal side, with dorsal setae distinct: subdistal setae only slightly stronger and denser than proximal (Fig. 53C).

*Female*: Unknown.

###### Affinities.

*Exocellina haia* sp. nov. can be distinguished by the shape and setation of its median lobe and paramere and/or by its size and colouration from the species co-occurring in the same area (*E. bacchusi*, *E. craterensis* Shaverdo & Balke, 2014, *E. damantiensis*, *E. hintelmannae* (Shaverdo, Sagata & Balke, 2005), *E. warasera* sp. nov.). For affinities within the group, see the “Key”.

###### Distribution.

Papua New Guinea: Simbu Province, Crater Mountain area (Fig. 54). The species is named after Haia Village. The name is a noun in the nominative singular standing in apposition.

##### 20. Exocelina kobau

Taxon classificationAnimaliaColeopteraDytiscidae

Shaverdo & Balke sp. nov.

110D3B4B-1C1C-5EC9-9F6E-781AEF4783D0

http://zoobank.org/6773EB08-F9C3-4CD8-A4C6-25B49D19AD5C

Figs 46 , 50

###### Type locality.

Papua New Guinea: Morobe Province, E Pindiu, Kobau, ca. 6°25'10.1"S, 147°32'06.6"E, 1400 m a.s.l.

###### Type material.

*Holotype*: male “PAPUA N.G.: Morobe Prov. E Pindiu, Kobau 24.4.1998, 1400 m, leg. A. Riedel”, “SEM 13” (NHMW).

###### Description.

*Body size and form*: Beetle medium-sized: TL-H 4.25 mm, TL 4.75 mm, MW 2.2 mm, with oblong-oval habitus.

**Figures 46–49.**
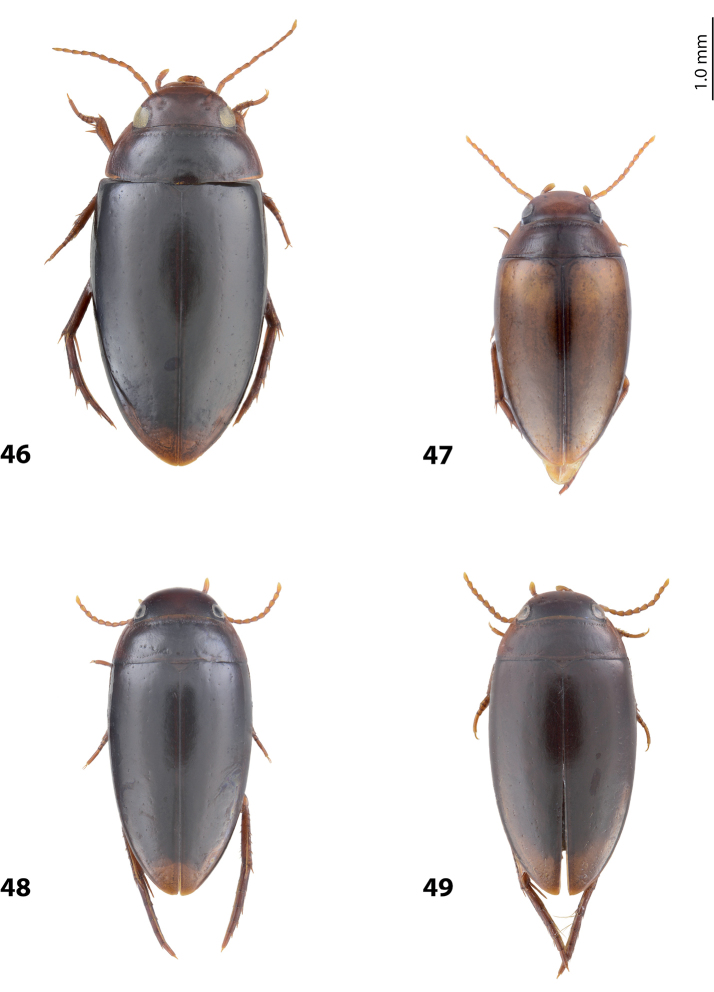
Habitus and colouration **46***Exocelina kobau* sp. nov. **47***E. pulchella* sp. nov. **48***E. warasera* sp. nov. **49***E. haia* sp. nov.

*Colouration*: Piceous, with paler sides of pronotum and head anteriorly. Head dark brown, paler anteriorly. Pronotum dark brown, with brown sides. Elytra piceous, with weakly indicated reddish sutural lines. Head appendages and legs proximally reddish, legs distally darker, reddish brown (Fig. 46).

*Surface sculpture*: Shiny dorsally, with extremely fine and sparse punctation and weakly impressed microreticulation. Head with fine and sparse punctation (spaces between punctures 2–3 times size of punctures); diameter of punctures equal to or smaller than diameter of cells of microreticulation. Pronotum with much finer and sparser punctation than on head, very inconspicuous. Punctation on elytra invisible. Pronotum and elytra with weakly impressed microreticulation; head with microreticulation slightly stronger. Metaventrite, metacoxae, and abdominal ventrites distinctly microreticulate. Metacoxal plates with longitudinal strioles and weak transverse wrinkles; abdominal ventrites with strioles. Punctation on venter invisible; inconspicuous on two last abdominal ventrites.

*Structures*: Pronotum with lateral bead. Base of prosternum and neck of prosternal process with distinct ridge, slightly rounded anteriorly. Blade of prosternal process lanceolate, relatively narrow, slightly convex, with distinct bead and few setae laterally. Abdominal ventrite 6 truncate.

**Figures 50, 51.**
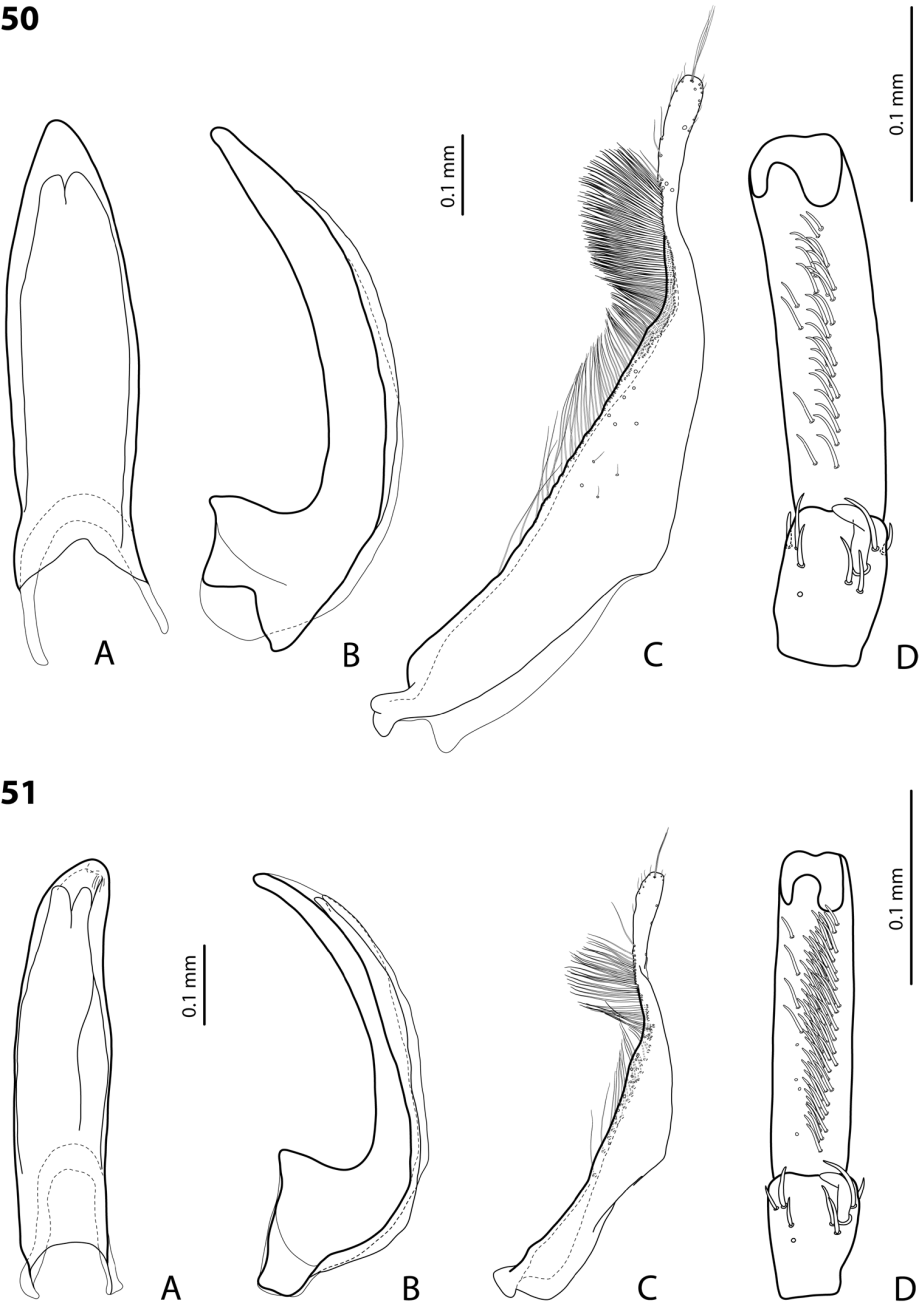
**50***Exocelina kobau* sp. nov. **51***E. pulchella* sp. nov. **A** median lobe in ventral view **B** median lobe in lateral view **C** paramere in external view **D** male protarsomeres 4–5 in ventral view.

*Male*: Protarsomere 4 with large, thick, strongly curved anterolateral hook-like seta. Protarsomere 5 ventrally with anterior band of 25 and posterior row of five relatively long setae (Fig. 50D). Abdominal ventrite 6 with 3–4 lateral striae on each side. Median lobe short, robust, evenly tapering to broadly pointed apex in lateral and ventral views (Fig. 50A, B). Paramere slightly concave on dorsal side, subdistal setae strong and dense, proximal setae inconspicuous (Fig. 50C).

*Female*: Unknown.

###### Affinities.

*Exocellina kobau* sp. nov. can be distinguished by its size, dorsal punctation, shape and setation of its median lobe and paramere, and large anterolateral hook-like seta of the male protarsomere 4 from the species co-occurring in the same area (*E. damantiensis*, *E. kabwumensis* Shaverdo & Balke, 2016, and *E. bacchusi*). For the affinities within the group, see the “Key”.

###### Distribution.

Papua New Guinea: Morobe Province (Fig. 54).

###### Etymology.

The species is named after Kobau Village. The name is a noun in the nominative singular standing in apposition.

##### 21. Exocelina pulchella

Taxon classificationAnimaliaColeopteraDytiscidae

Shaverdo & Balke sp. nov.

FA0E68D4-F22A-587F-9675-ABD615E5B6FB

http://zoobank.org/48FF81EF-7022-493E-9B4E-719933174A43

Figs 47 , 51

Exocelina undescribed sp. MB3408: Toussaint et al. 2014: supplementary figs 1–4, tab. 2; Toussaint et al. 2015: supplementary figs S1, S2, tab. S3, and information S5, S6.

###### Type locality.

Papua New Guinea: Central Province, Moroka, Kailaki, Wareaga, 09°25.42'S, 147°31.07'E, 760 m a.s.l.

###### Type material.

*Holotype*: male “Papua New Guinea Central, Moroka, Kailaki Wareaga, 760m, 27x2009 9.25.424S 147.31.068E Sagata (PNG227)” (ZSM). *Paratypes*: 24 males, 43 females with the same label as the holotype, one male with an additional green label “DNA M.Balke 3832” (NHMW, ZSM). 1 male “Papua New Guinea: Central, Moroka area, Kailaki, 827 m, 26.x.2009, 9.24.134S 147.33.521E, Sagata (PNG225)” (ZSM). 5 males, 9 females “Papua New Guinea: Central, 755m, 28.x.2009 S9 25 47 5 E147 32 59.1, Sagata (PNG229)”, one male with an additional green label “DNA M.Balke 3831” (NHMW, ZSM). 2 males, 1 female “Papua New Guinea: Central, Kokoda Trek, 320m, i.2008 09 19.236S 147.31.791E, Posman (PNG 168)”, one male with a green label “DNA M.Balke 3403” (ZSM). 2 males, 2 females “Papua New Guinea: Central, Kokoda Trek, 980m, i.2008, 09 15.933S 147.36.590E, Posman (PNG 169)” (NHMW, ZSM). 1 female “Papua New Guinea: Central, Kokoda Trek, 590m, i.2008, 09 14.339S 147.36.920E, Posman (PNG 170)” (ZSM). 3 males “Papua New Guinea: Central, Myola, 1110m, i.2008, 09 12.630S 147.31.880E, Posman (PNG 177)”, one of them with an additional green label “DNA M.Balke 3408” (ZSM).

###### Description.

*Body size and form*: Beetle small: TL-H 2.85–3.3 mm, TL 3.15–3.7 mm, MW 1.6–1.8 mm (holotype: TL-H 3.05 mm, TL 3.4 mm, MW 1.75 mm), with oblong-oval habitus.

*Colouration*: Reddish head and bicoloured elytra: yellowish at shoulders and brownish distally. Head reddish, reddish brown posterior eyes. Pronotum reddish brown to brown on disc (broader or narrower) and yellowish to yellowish reddish on sides. Elytra bicoloured: yellowish in proximal 1/4 to 1/3 (rarely to 1/2) and yellowish brown to brown distally, proximal yellowish colouration sometimes more distinctly boarded as shoulder spots slightly elongated along sutural lines, but mostly fuzzy, not boarded. Head appendages and legs proximally yellowish, legs distally darker, reddish to reddish brown (Fig. 47). Teneral specimens paler.

*Surface sculpture*: Shiny dorsally, with extremely fine and sparse punctation and weakly impressed microreticulation. Head with fine and sparse punctation (spaces between punctures 2–3 times size of punctures); diameter of punctures smaller than diameter of cells of microreticulation. Pronotum with much finer and sparser punctation than on head, very inconspicuous. Punctation on elytra invisible. Pronotum and elytra with weakly impressed microreticulation; head with microreticulation slightly stronger. Metaventrite, metacoxae, and abdominal ventrites distinctly microreticulate. Metacoxal plates with longitudinal strioles and weak transverse wrinkles; abdominal ventrites with strioles. Punctation on venter invisible; inconspicuous on two last abdominal ventrites.

*Structures*: Pronotum with lateral bead. Base of prosternum and neck of prosternal process with distinct ridge, slightly rounded anteriorly. Blade of prosternal process lanceolate, relatively narrow, slightly convex, with distinct bead and few setae laterally. Abdominal ventrite 6 broadly rounded or slightly truncate.

*Male*: Protarsomere 4 with large, thick, strongly curved anterolateral hook-like seta. Protarsomere 5 ventrally with anterior band ca. 60 and posterior row of eight relatively long setae (Fig. 51D). Abdominal ventrite 6 with 4–7 lateral striae on each side. Median lobe simple, short, slightly curved, with broadly pointed apex in lateral view, and evenly tapering to broadly pointed apex in ventral view (Fig. 51A, B). Paramere slightly concave on dorsal side, with strong, long, dense subdistal setae, proximal setae inconspicuous (Fig. 51C).

*Female*: Without evident differences in external morphology from males, except for not modified pro- and mesotarsi and abdominal ventrite 6 without striae.

###### Affinities.

From species of the *E. danae*, *E. bacchusi* and *E. jaseminae* groups (Shaverdo et al. 2016d) known from Central Province, *E. pulchella* sp. nov. can be easily distinguished by its small size, characteristic colouration, extremely fine dorsal punctation, and shape of the median lobe. For the affinities within the group, see the “Key”.

###### Distribution.

Papua New Guinea: Central Province (Fig. 54).

###### Etymology.

The species name derives from Latin *pulchellus*, a diminutive of *pulcher* (beautiful), to express the small size and nice colouration of the beetles; this species is the most colourful of all known New Guinea *Exocelina*. The species name is an adjective in the nominative singular.

##### 22. Exocelina warasera

Taxon classificationAnimaliaColeopteraDytiscidae

Shaverdo & Balke sp. nov.

FE62B5E8-DBFA-5779-BE0D-26BE7C8E920A

http://zoobank.org/DC3A4143-1103-4192-8401-C40780202ED8

Figs 48 , 52

Exocelina undescribed sp. MB0261: Toussaint et al. 2014: supplementary figs 1–4, tab. 2; Toussaint et al. 2015: supplementary figs S1, S2, tab. S3, and information S5, S6.

###### Type locality.

Papua New Guinea: Simbu Province, between Supa and Haia Villages (Airstrips), ca. 6°39'39.9"S, 145°00'28.4"E, 1032 m.

###### Type material.

*Holotype*: male “Papua New Guinea: Supa Haia, 1023m, 10.ix.2002, K.Sagata (WB1)” (ZSM). *Paratypes*: **Simbu**: 5 males with the same label as the holotype (NHMW, ZSM). 3 males, 3 females “Papua New Guinea: Simbu/EHPr. Crater Mountain, trek Haia - Wara Sera, 750 m, 12IX2002, Balke & Sagata, (PNG 2)” (NHMW, ZSM). 4 males “Papua New Guinea: Crater Mountain, trek Haia - Wara Sera, 600m, 12IX2002, Balke & Sagata, (PNG 003)” (NHMW, ZSM). 2 males, 1 female “Papua New Guinea: Crater Mountain, trek Haia - Wara Sera, 500m, 12IX2002, Balke & Sagata, (PNG 004)” (ZSM). 2 males “Papua New Guinea: Crater Mountain, trek Haia - Wara Sera, 500m, 12IX2002, Balke & Sagata, (PNG 005)” (ZSM). 1 male, 3 females “Papua New Guinea Simbu/EHPr. Crater Mountain, WaraSera Station, 820 m, 14IX2002, Balke & Sagata (PNG 8)” (ZSM). **Simbu/Eastern Highlands**: 4 males “Papua New Guinea: Simbu/EHPr. Crater Mountain, Sera - Herowana, Wara Pima, 900 m, 15IX2002, Balke & Sagata (PNG 011)” (NHMW, ZSM). 1 male “261 DNA M Balke” [green], “PNG Simbu/EHP, Crater Mountain, Sera - Herowana, upper Oh River, 1200 m, 15IX2002, Balke & Sagata (PNG 012)”, “sp.21 SEM 19” (ZSM). 1 male “Papua New Guinea: Crater Mountain, Sera - Herowana, upper Oh River, 1200 m, 15IX2002, Balke & Sagata (PNG 012)” (ZSM). 3 males “Papua New Guinea: Simbu/EHPr. Crater Mountain, Sera - Herowana, Jau river, 1000 m, 15IX2002, Balke & Sagata (PNG 015)” (NHMW, ZSM). 1 female “266 DNA M Balke” [green], “PNG Simbu / EHPr. Crater Mountain, Sera - Herowana, Wara Hulene, 1000 m, 16IX2002, Balke & Sagata (PNG 17)” (ZSM). 2 females “Papua New Guinea: Simbu / EHPr. Crater Mountain, Sera - Herowana, Hulene river, 1000m, 16IX2002, Balke & Sagata (PNG 017)” (ZSM).

###### Females of doubtful identity.

See for *E. bacchusi*.

###### Description.

*Body size and form*: Beetle small: TL-H 3.15–3.8 mm, TL 3.5–4.15 mm, MW 1.65–2.05 mm (holotype: TL-H 3.4 mm, TL 3.8 mm, MW 1.8 mm), with oblong-oval habitus.

*Colouration*: Dark brown, with paler sides of pronotum and head anteriorly. Head dark brown, paler anteriorly. Pronotum dark brown, with brown sides. Elytra uniformly dark brown. Head appendages and legs proximally reddish, legs distally darker, reddish brown (Fig. 48). Teneral specimen paler, brown to reddish brown with yellowish pronotal sides and head anteriorly.

*Surface sculpture*: Shiny dorsally, with extremely fine and sparse punctation and weakly impressed microreticulation. Head with fine and sparse punctation (spaces between punctures 2–3 times size of punctures); diameter of punctures smaller than diameter of cells of microreticulation. Pronotum with much finer and sparser punctation than on head, very inconspicuous. Punctation on elytra invisible. Pronotum and elytra with weakly impressed microreticulation; head with microreticulation slightly stronger. Metaventrite, metacoxae, and abdominal ventrites distinctly microreticulate. Metacoxal plates with longitudinal strioles and weak transverse wrinkles; abdominal ventrites with strioles. Punctation on venter invisible; inconspicuous on two last abdominal ventrites.

*Structures*: Pronotum with lateral bead. Base of prosternum and neck of prosternal process with distinct ridge, slightly rounded anteriorly. Blade of prosternal process lanceolate, relatively narrow, slightly convex, with distinct bead and few setae laterally. Abdominal ventrite 6 truncate or very slightly concave.

**Figures 52, 53.**
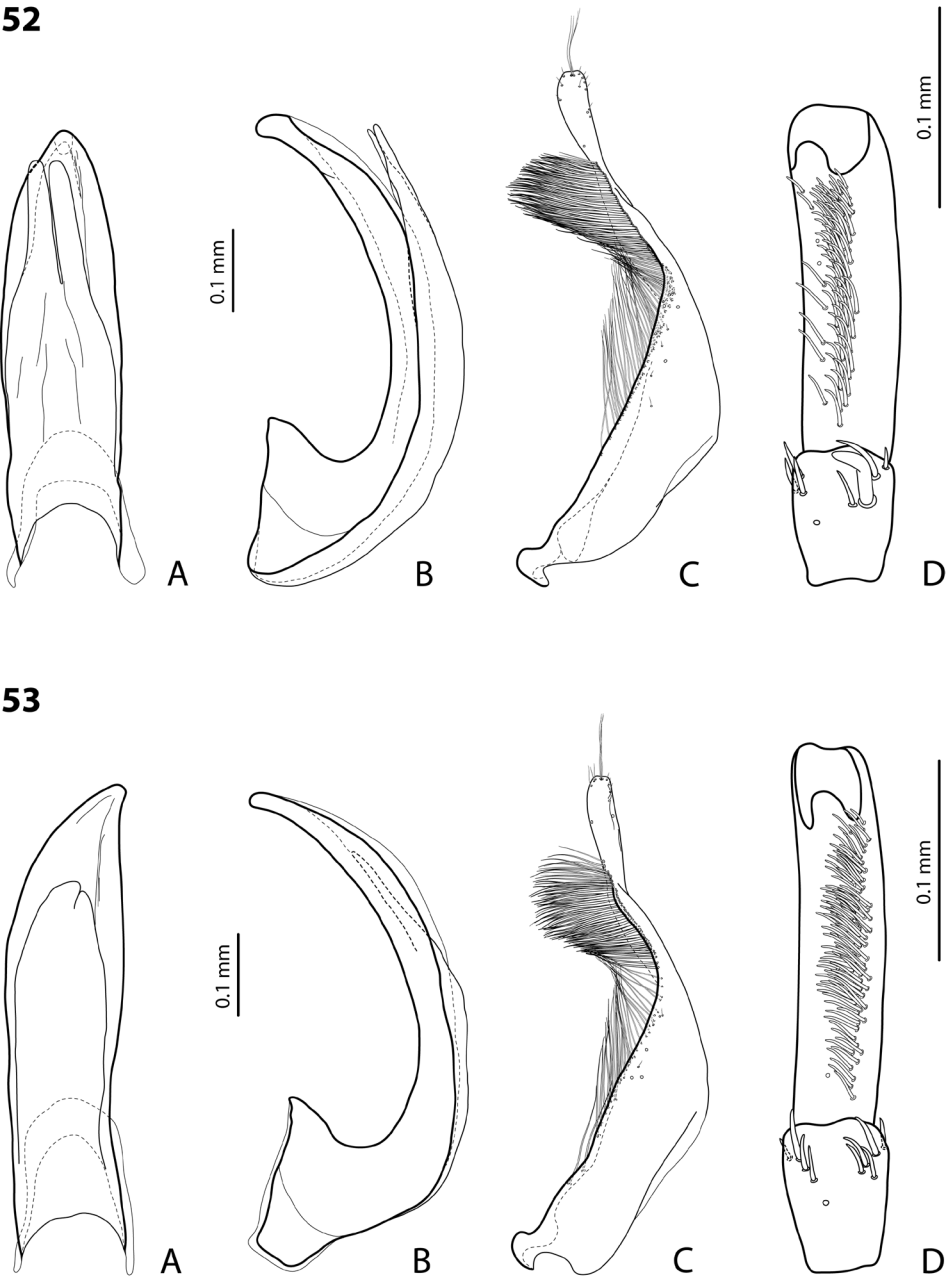
**52***Exocelina warasera* sp. nov. **53***E. haia* sp. nov. **A** median lobe in ventral view **B** median lobe in lateral view **C** paramere in external view **D** male protarsomeres 4–5 in ventral view.

*Male*: Protarsomere 4 with large, thick, strongly curved anterolateral hook-like seta. Protarsomere 5 ventrally with anterior band of ca. 50 and posterior row of eight relatively long setae (Fig. 52D). Abdominal ventrite 6 with 2–4 lateral striae on each side. Median lobe short, curved, with broadly pointed apex in lateral view, and evenly tapering to pointed apex in ventral view; its right lateral margin slightly concave at apex (Fig. 52A, B). Paramere slightly concave on dorsal side, its subdistal part with numerous, dense, very strong setae, proximal setae long, but weaker, less distinct (Fig. 52C).

*Female*: Without evident differences in external morphology from males, except for not modified protarsi and abdominal ventrite 6 without striae.

###### Affinities.

From the species co-occurring in the same area (*E. bacchusi*, *E. craterensis*, *E. damantiensis*, *E. hintelmannae*, and *E. haia* sp. nov.), *E. warasera* sp. nov. can be distinguished by the shape and setation of its median lobe and paramere and/or by its size and colouration. For the affinities within the group, see the “Key”.

###### Distribution.

**Figure 54.**
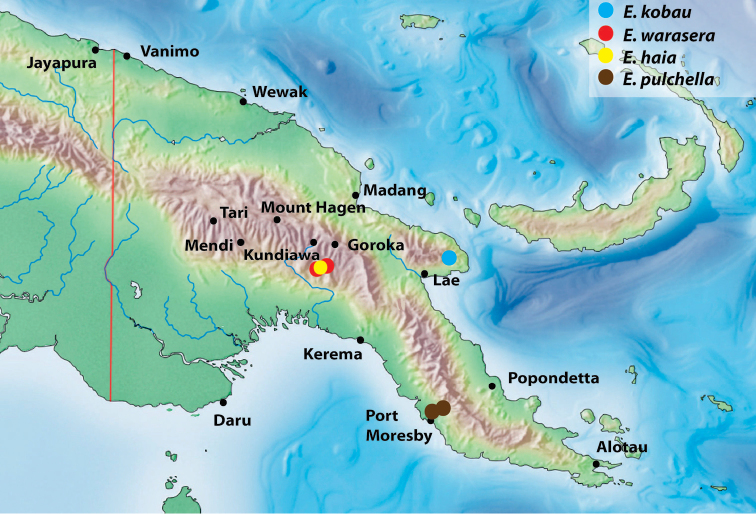
Map of the eastern part of New Guinea showing distribution of the species of the *E. warasera* group.

Papua New Guinea: Simbu and Eastern Highlands Provinces, Crater Mountain area (Fig. 54).

###### Etymology.

The species is named after Haia Village. The name is a noun in the nominative singular standing in apposition.

##### Key to species of *Exocelina warasera* group

**Table:** 

1	Beetle larger, TL-H 4.25 mm (Fig. 46)	***kobau* sp. nov.**
–	Beetle smaller, TL-H 2.85–3.8 mm (e.g., Fig. 47)	**2**
2	Beetle colourful, with reddish head and bicoloured elytra: yellowish at shoulders and brownish distally; smaller, TL-H 2.85–3.3 mm (Fig. 47)	***pulchella* sp. nov.**
–	Beetle dark brown, with paler sides of pronotum and head anteriorly; larger, TL-H 3.15–3.8 mm (Figs 48, 49)	**3**
3	Apex of median lobe shorter and thicker, with right lateral margin slightly concave (Fig. 52)	***warasera* sp. nov.**
–	Apex of median lobe longer and thinner, its lateral margins straight (Fig. 53)	***haia* sp. nov.**

## Supplementary Material

XML Treatment for Exocelina aipomek

XML Treatment for Exocelina koroba

XML Treatment for Exocelina mekilensis

XML Treatment for Exocelina morobensis

XML Treatment for Exocelina akameku

XML Treatment for Exocelina bacchusi

XML Treatment for Exocelina bacchusi herzogensis

XML Treatment for Exocelina erteldi

XML Treatment for Exocelina oiwa

XML Treatment for Exocelina oksibilensis

XML Treatment for Exocelina aseki

XML Treatment for Exocelina jaseminae

XML Treatment for Exocelina kailaki

XML Treatment for Exocelina pseudojaseminae

XML Treatment for Exocelina larsoni

XML Treatment for Exocelina nomax

XML Treatment for Exocelina warahulenensis

XML Treatment for Exocelina mianminensis

XML Treatment for Exocelina takime

XML Treatment for Exocelina haia

XML Treatment for Exocelina kobau

XML Treatment for Exocelina pulchella

XML Treatment for Exocelina warasera
